# Supplementary figures and images for: Retinoic acid-induced protein 14 controls dendritic spine dynamics associated with depressive-like behaviors
Source: eLife. 2022 Apr 25;11:e77755. doi: 10.7554/eLife.77755 (PMC9068211; doi:10.7554/eLife.77755)

**E**

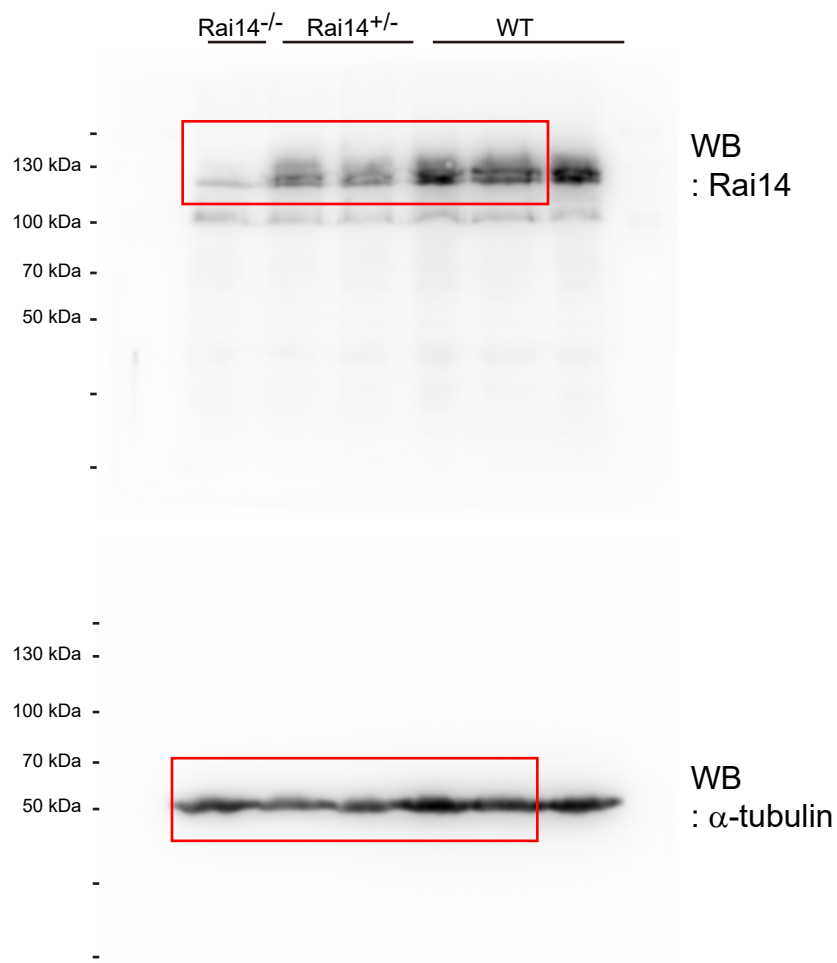

**Figure1–figure supplement 1**

Supplement: Figure 1—figure supplement 1—source data 1. [file elife-77755-fig1-figsupp1-data1.pdf]

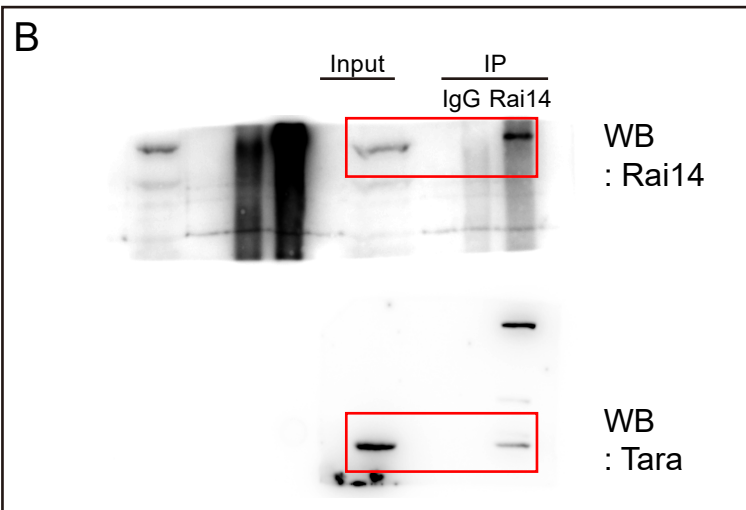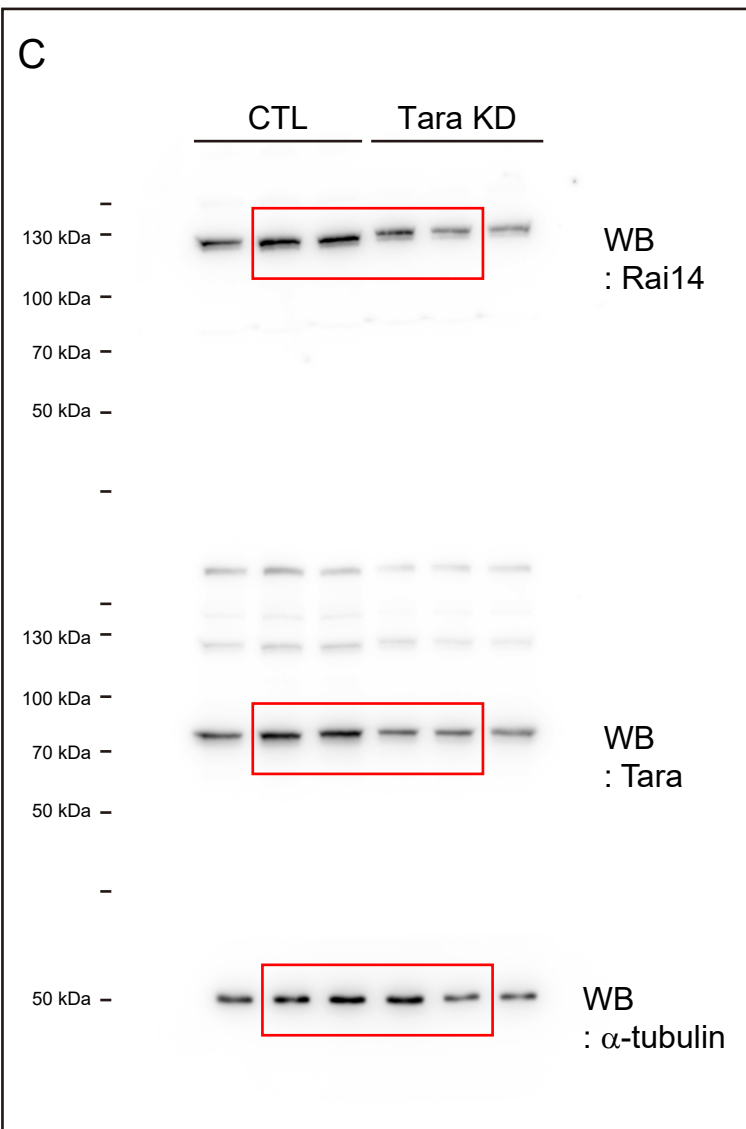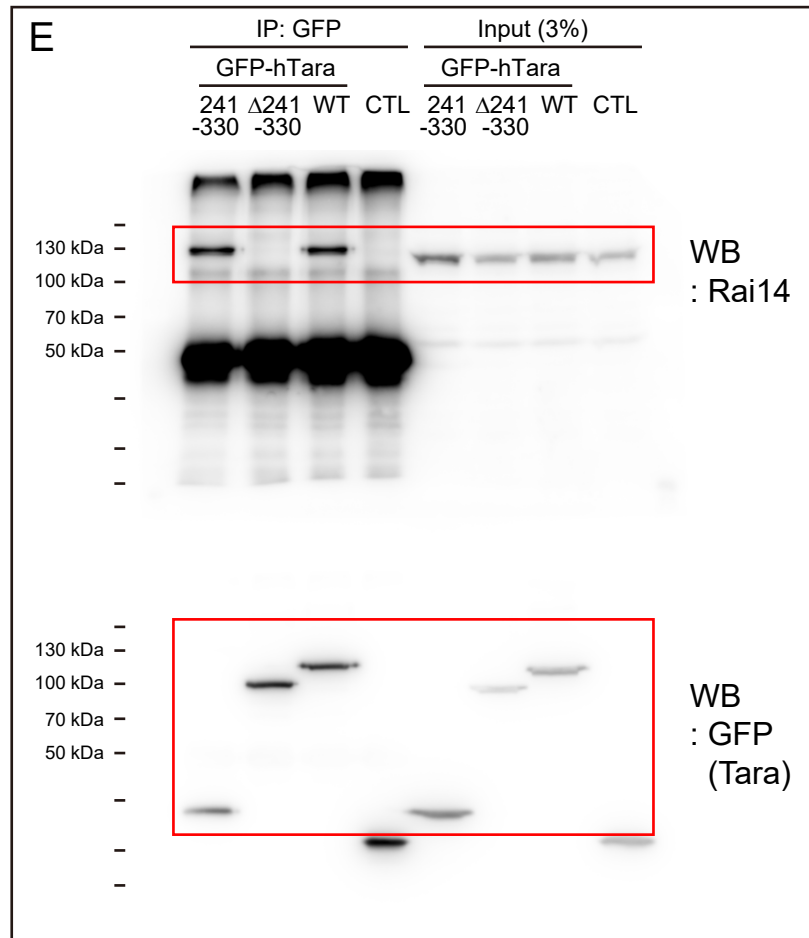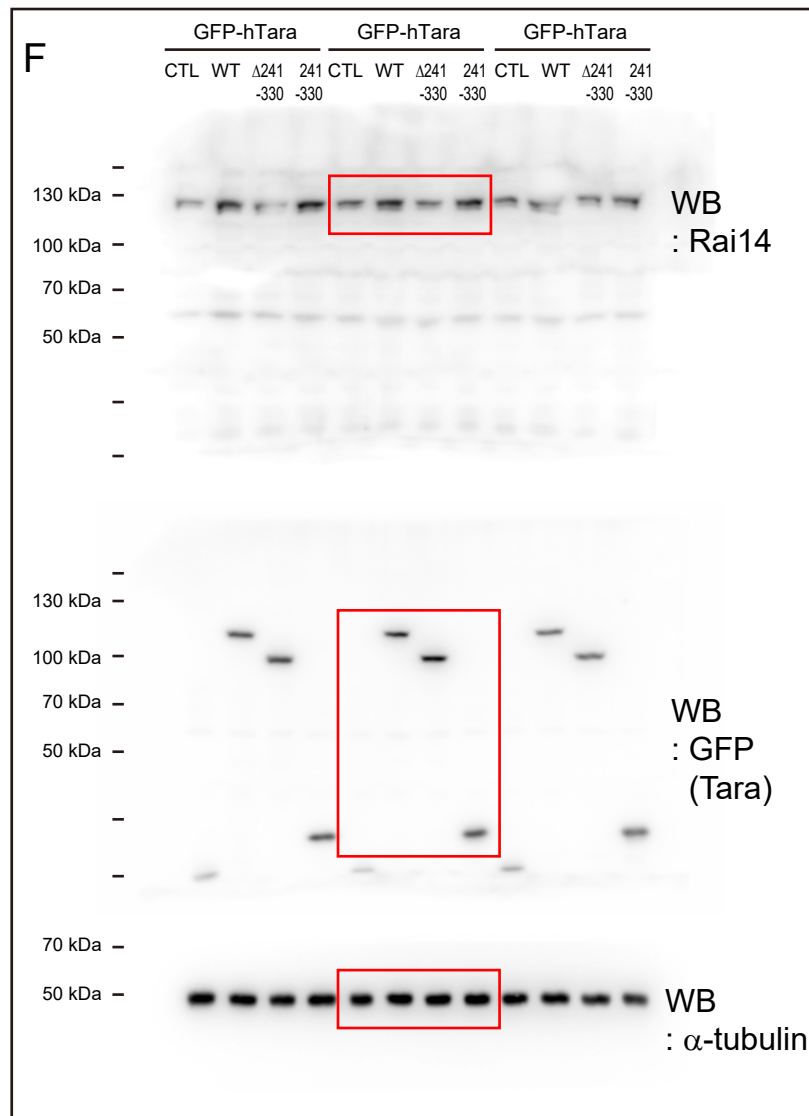

G

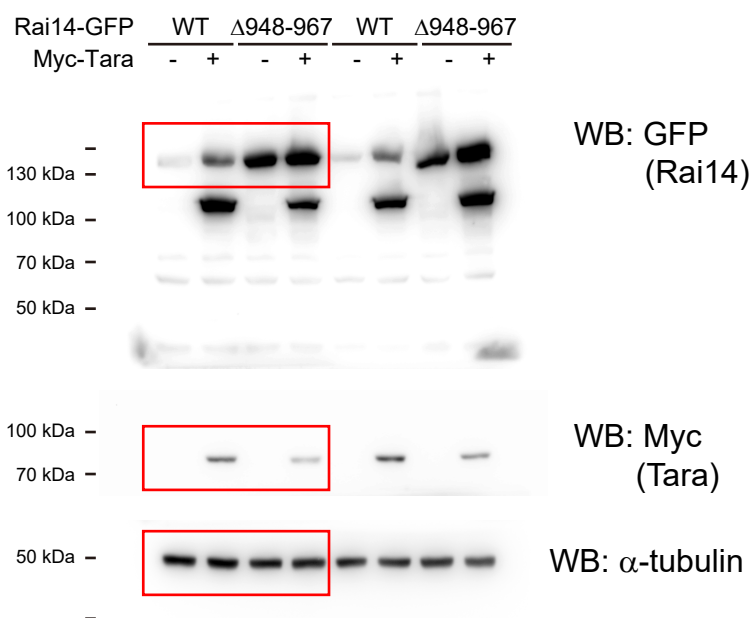

Supplement: Figure 2—source data 2. [file elife-77755-fig2-data2.pdf]

**B**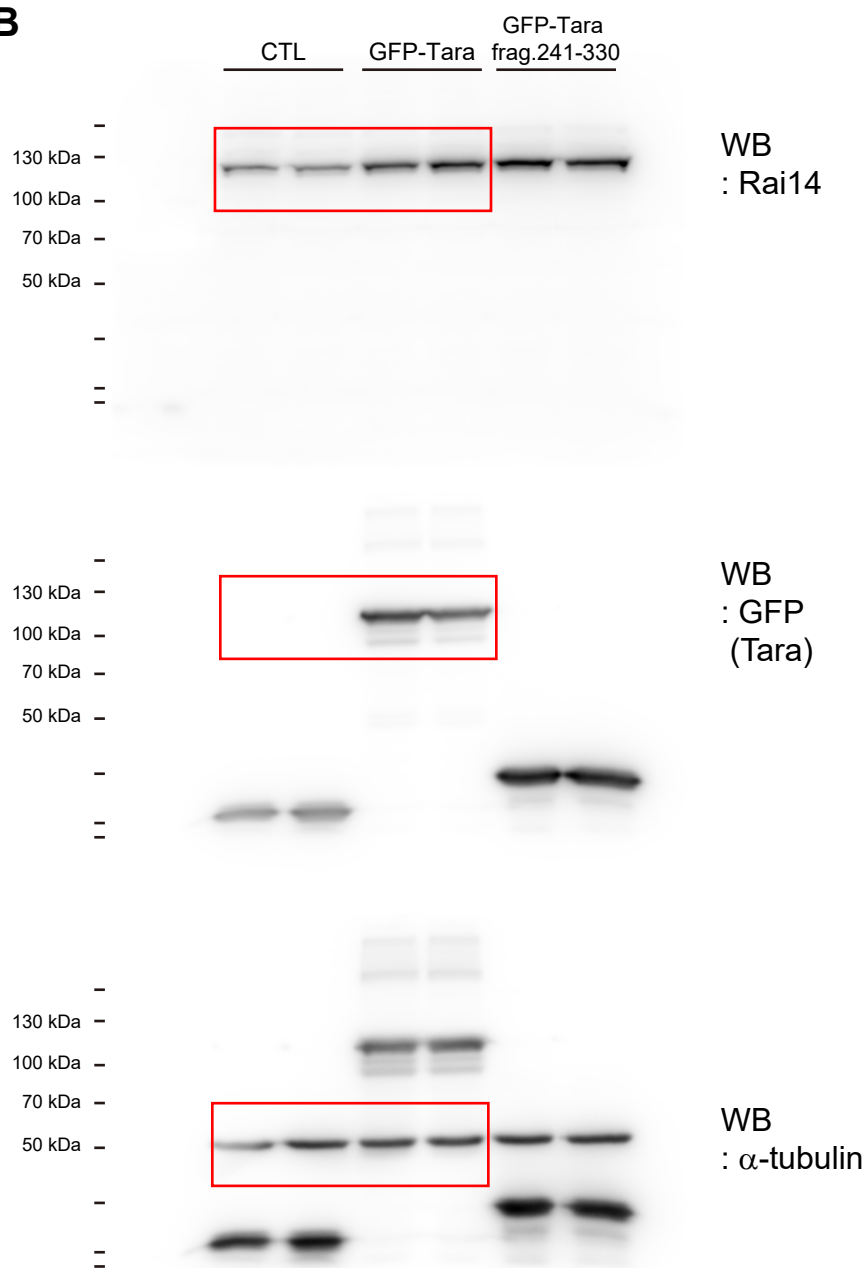

Supplement: Figure 2—figure supplement 1—source data 1. [file elife-77755-fig2-figsupp1-data1.pdf]

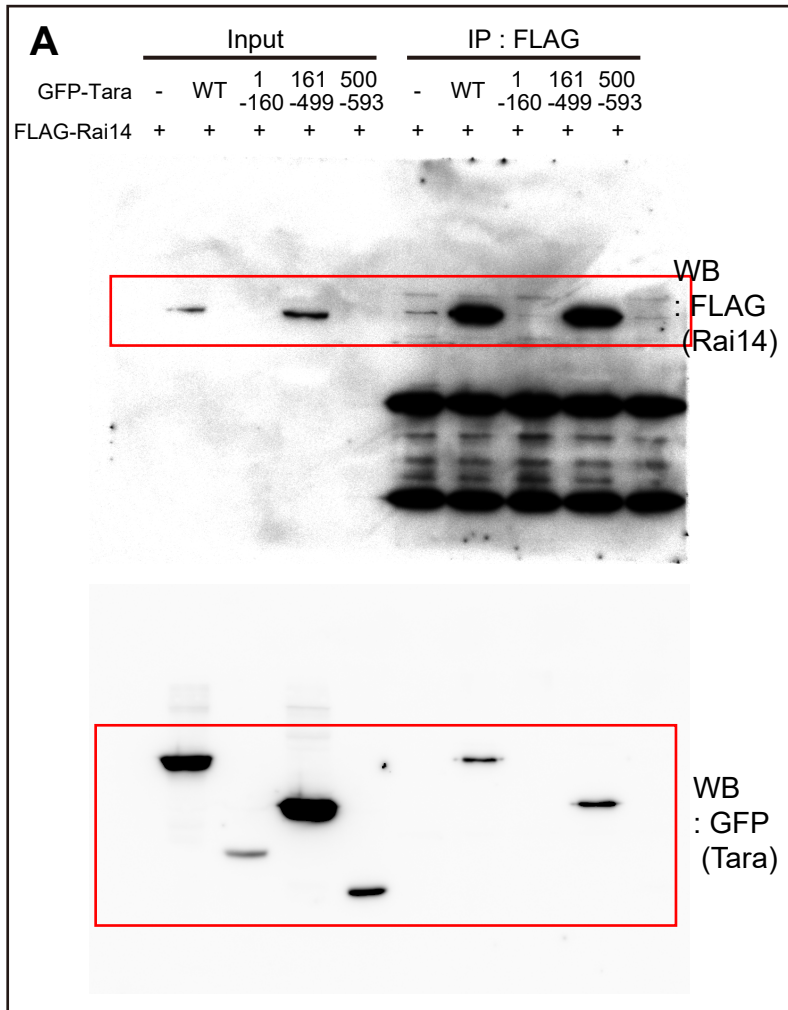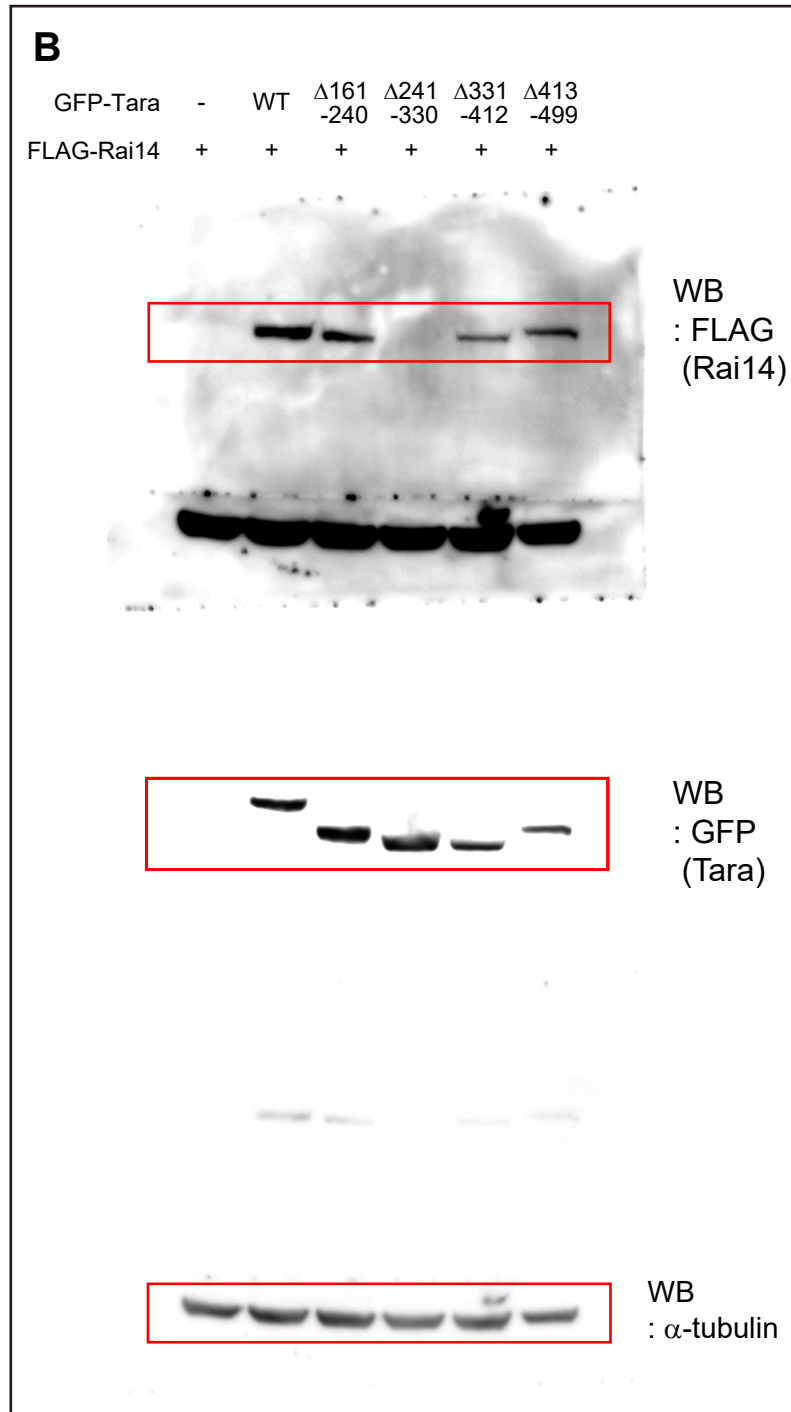

Figure2-figure supplement 2

Supplement: Figure 2—figure supplement 2—source data 1. [file elife-77755-fig2-figsupp2-data1.pdf]

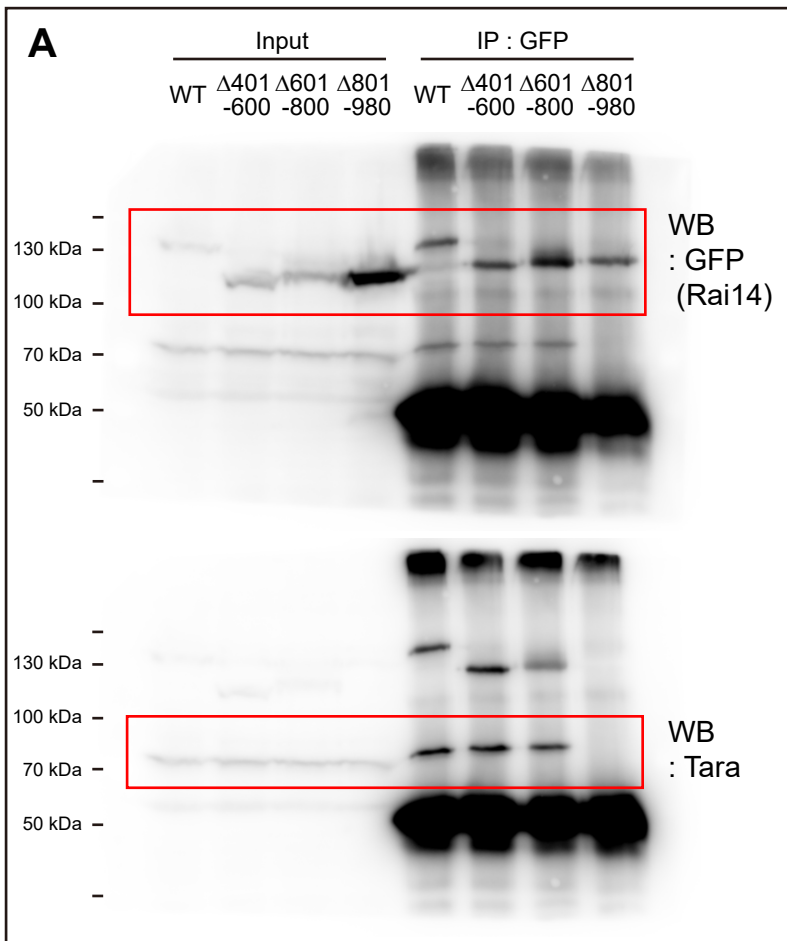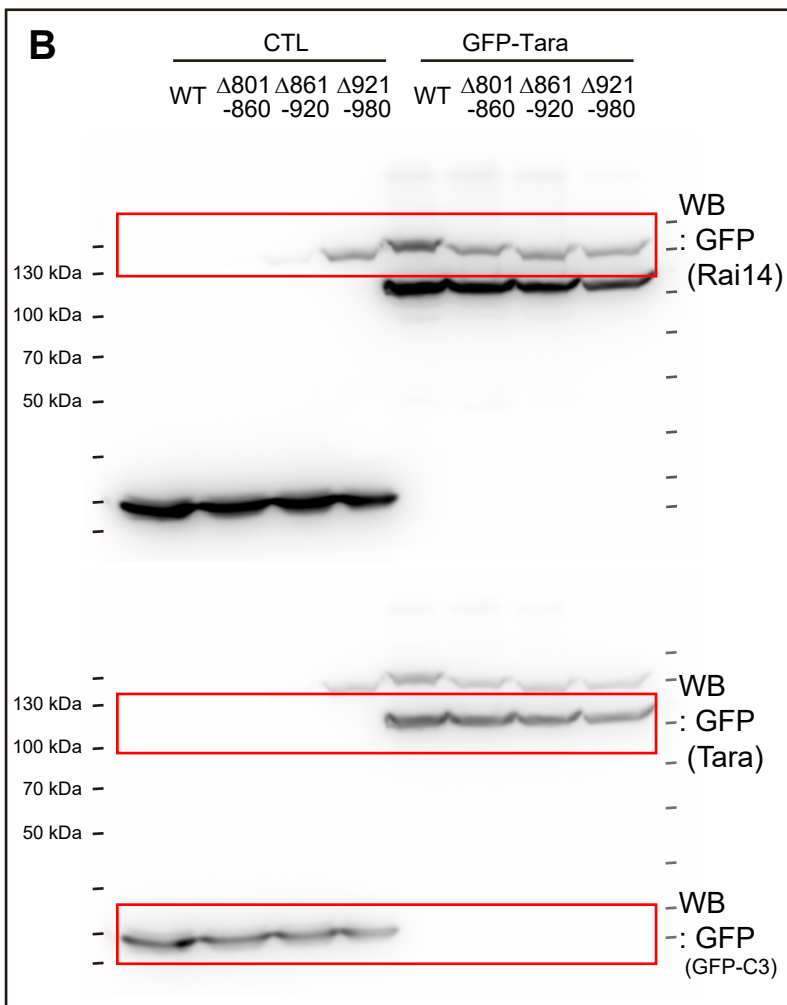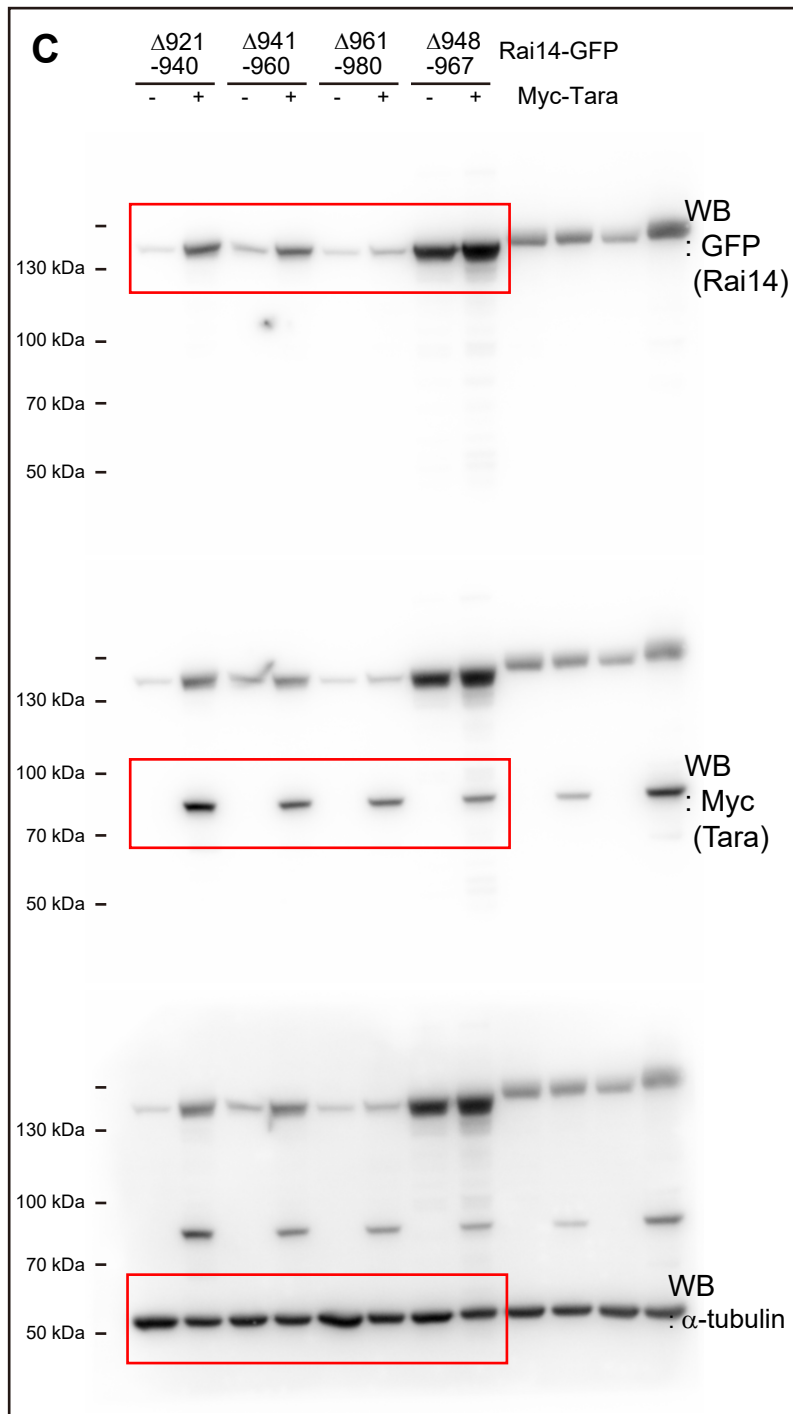

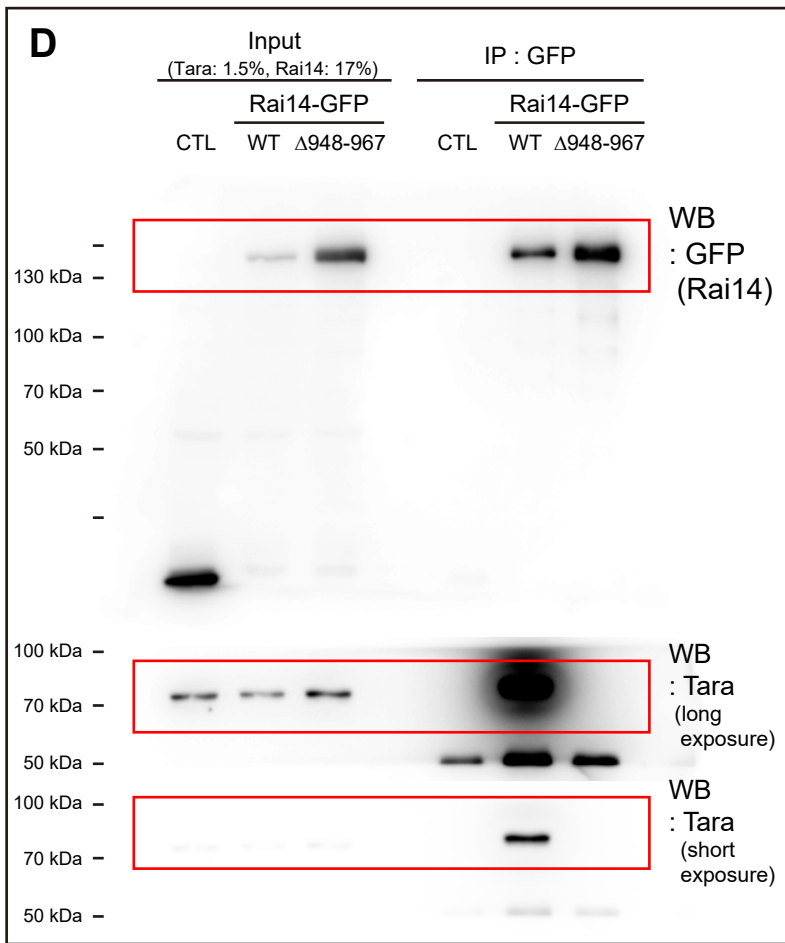

Supplement: Figure 2—figure supplement 3—source data 1. [file elife-77755-fig2-figsupp3-data1.pdf]

K

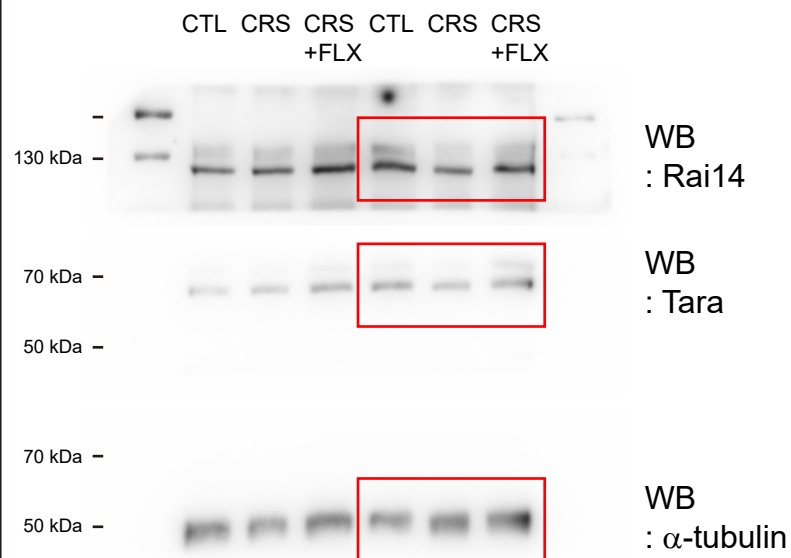

Figure 5

Supplement: Figure 5—source data 2. [file elife-77755-fig5-data2.pdf]

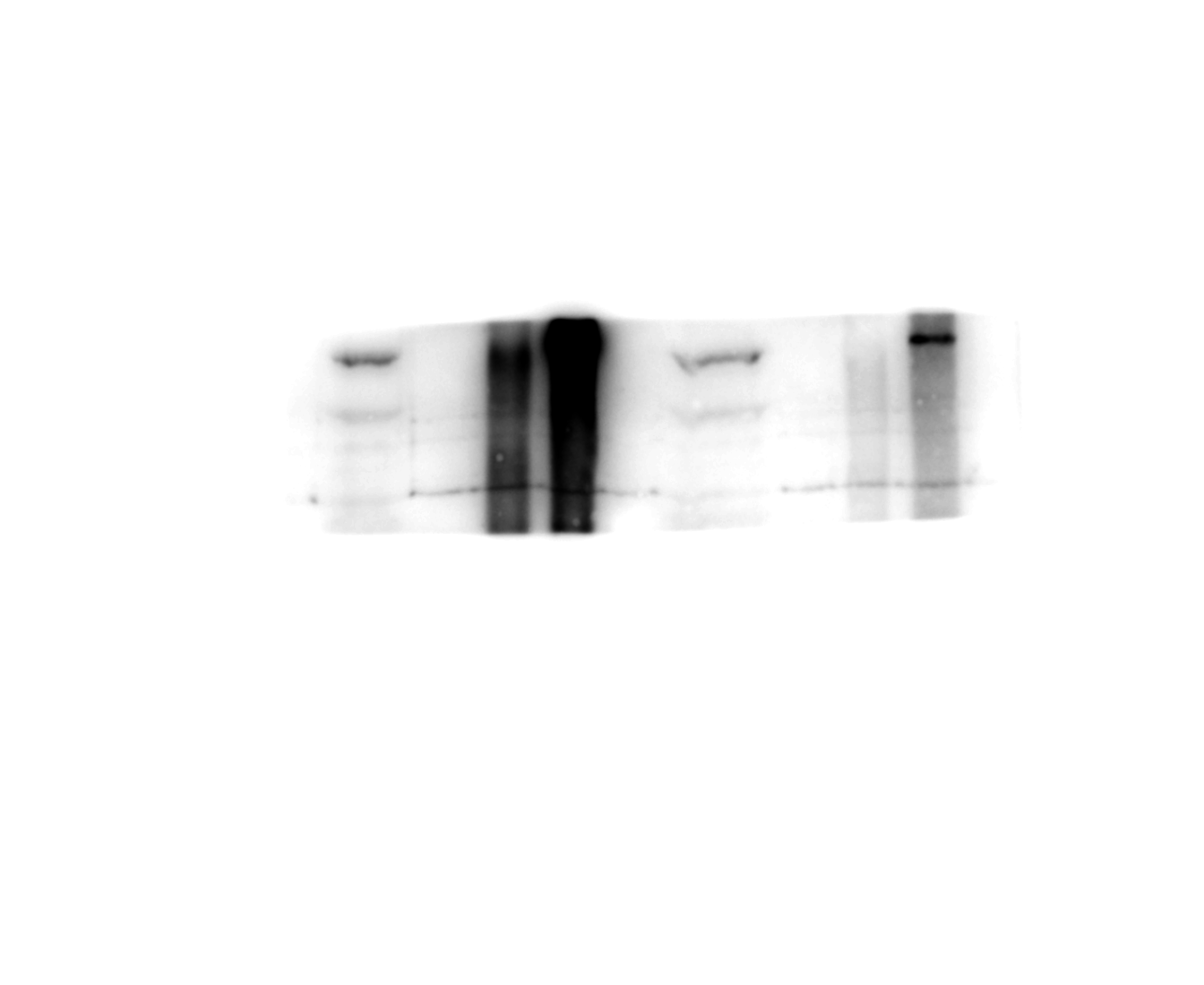

Supplement: Source data 1. [file elife-77755-data1.zip › Figure 2/Figure 2B Rai14.tif]

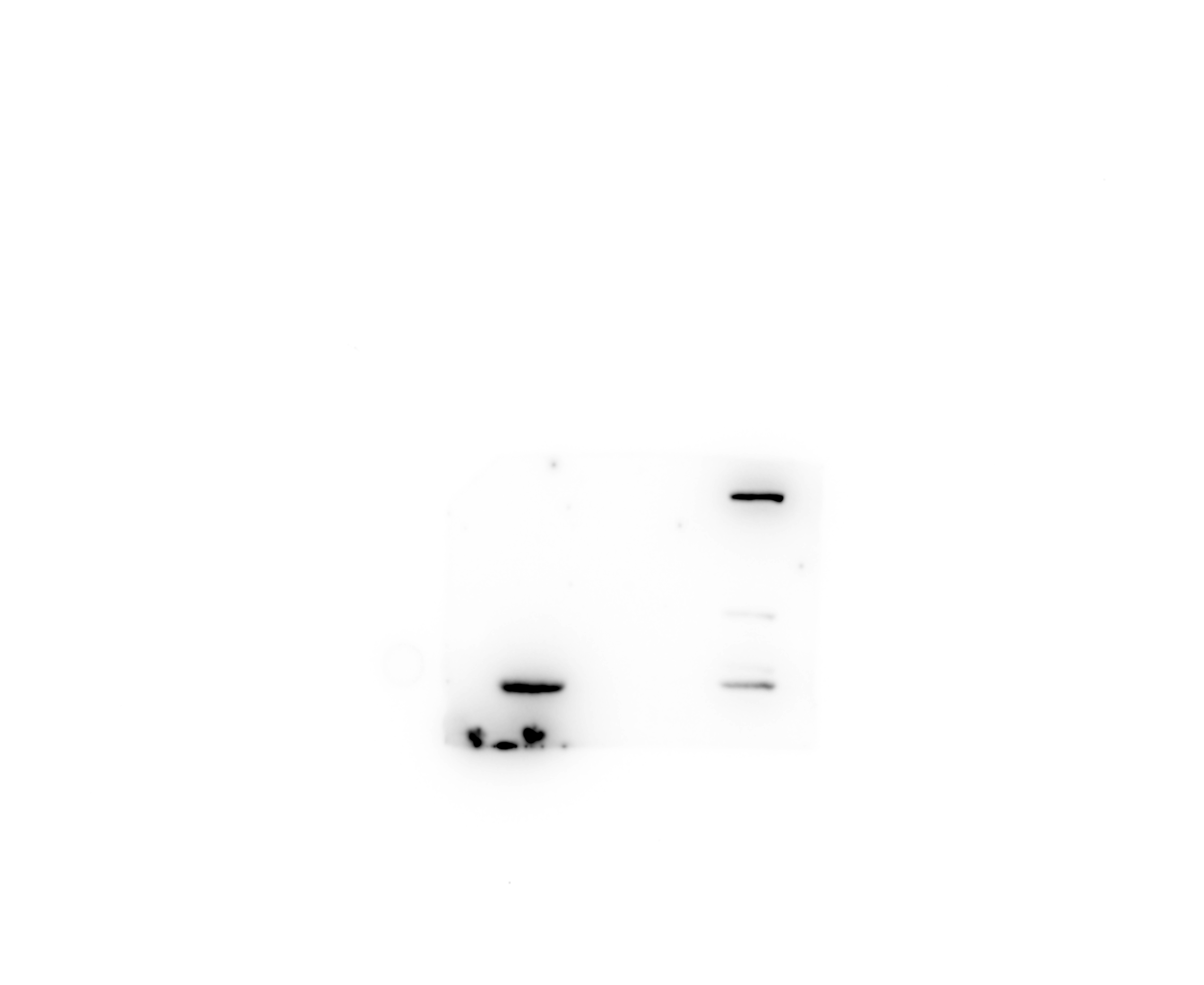

Supplement: Source data 1. [file elife-77755-data1.zip › Figure 2/Figure 2B Tara.tif]

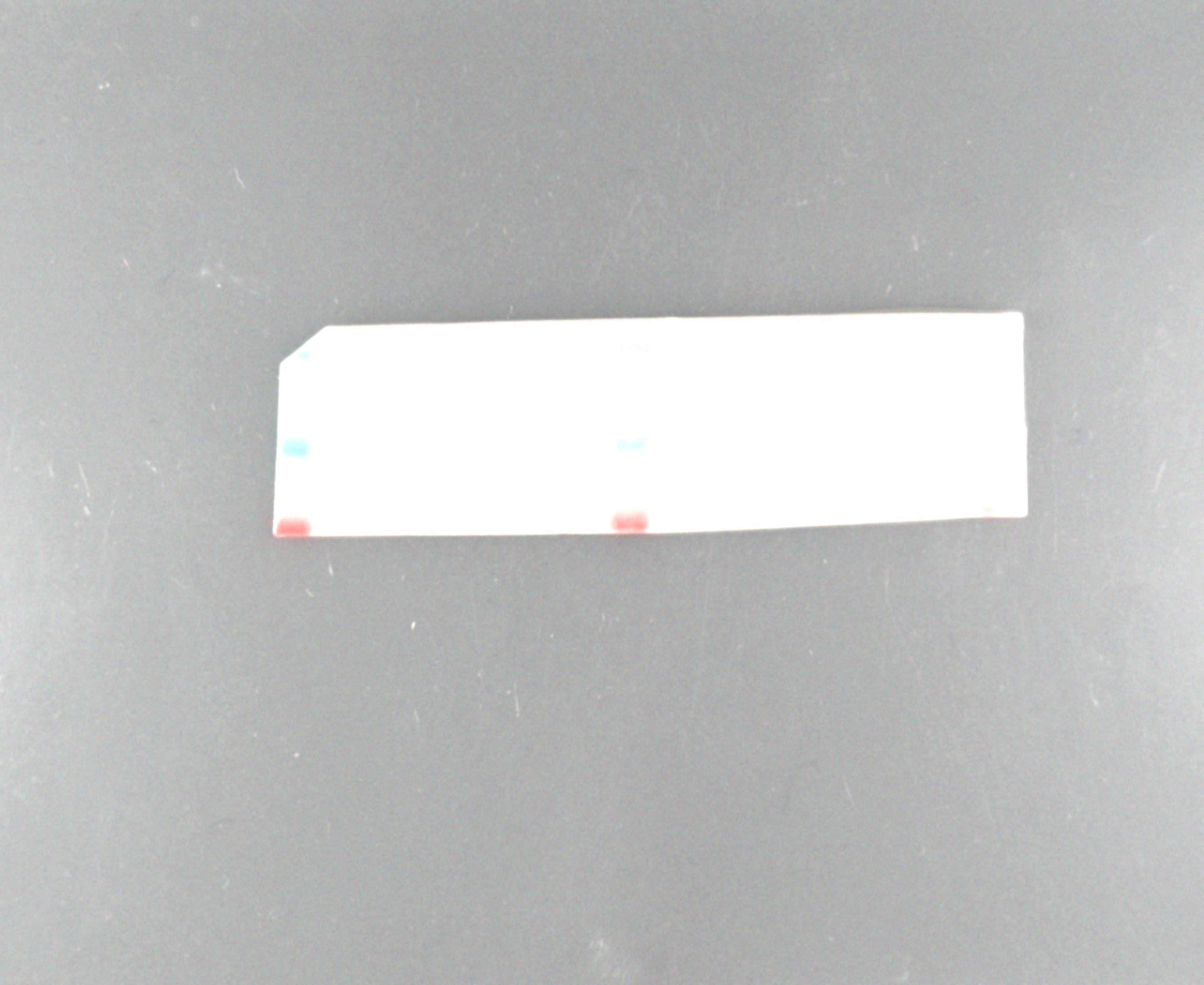

Supplement: Source data 1. [file elife-77755-data1.zip › Figure 2/Figure 2B-size marker for Rai14.tif]

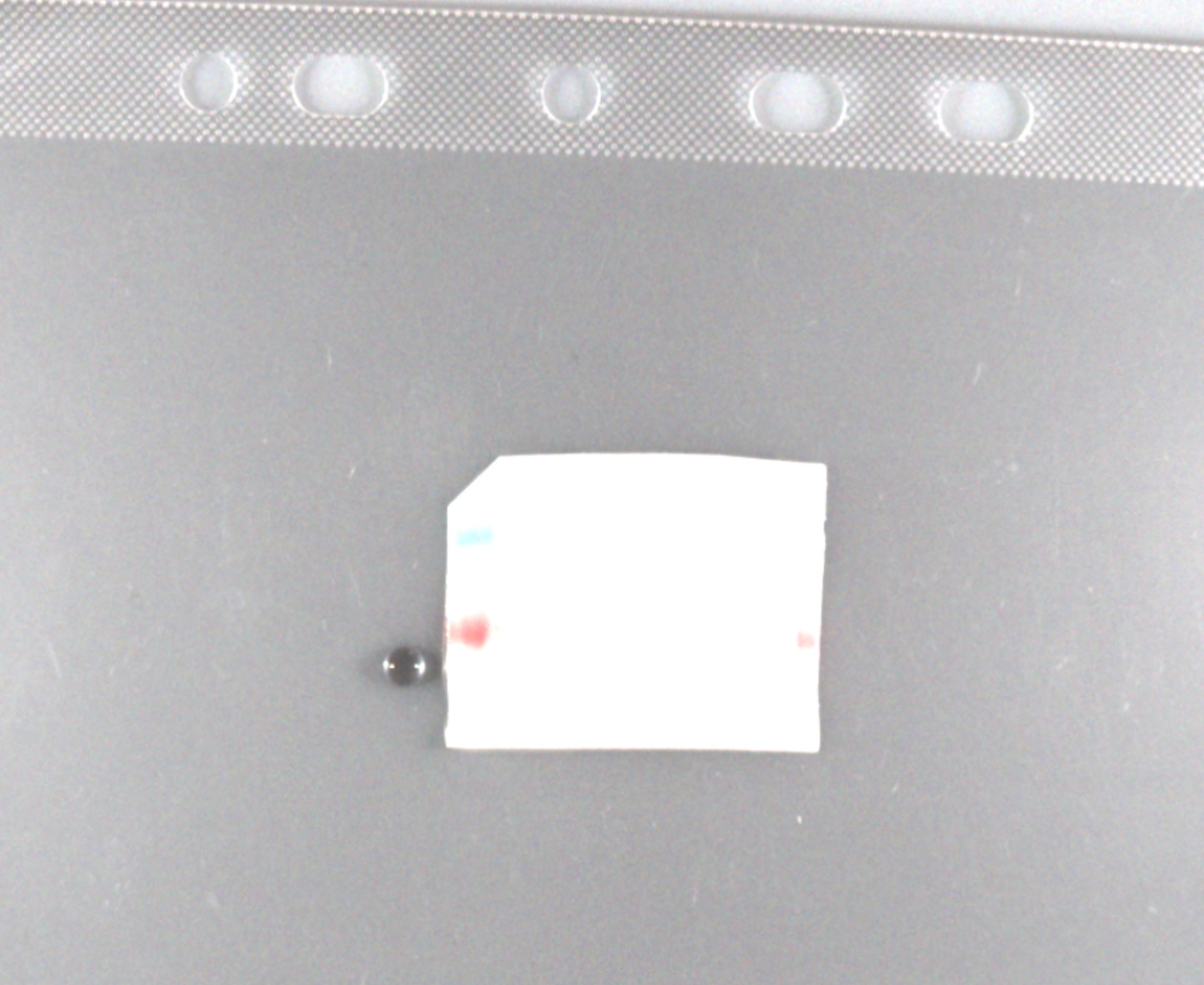

Supplement: Source data 1. [file elife-77755-data1.zip › Figure 2/Figure 2B-size marker for Tara.tif]

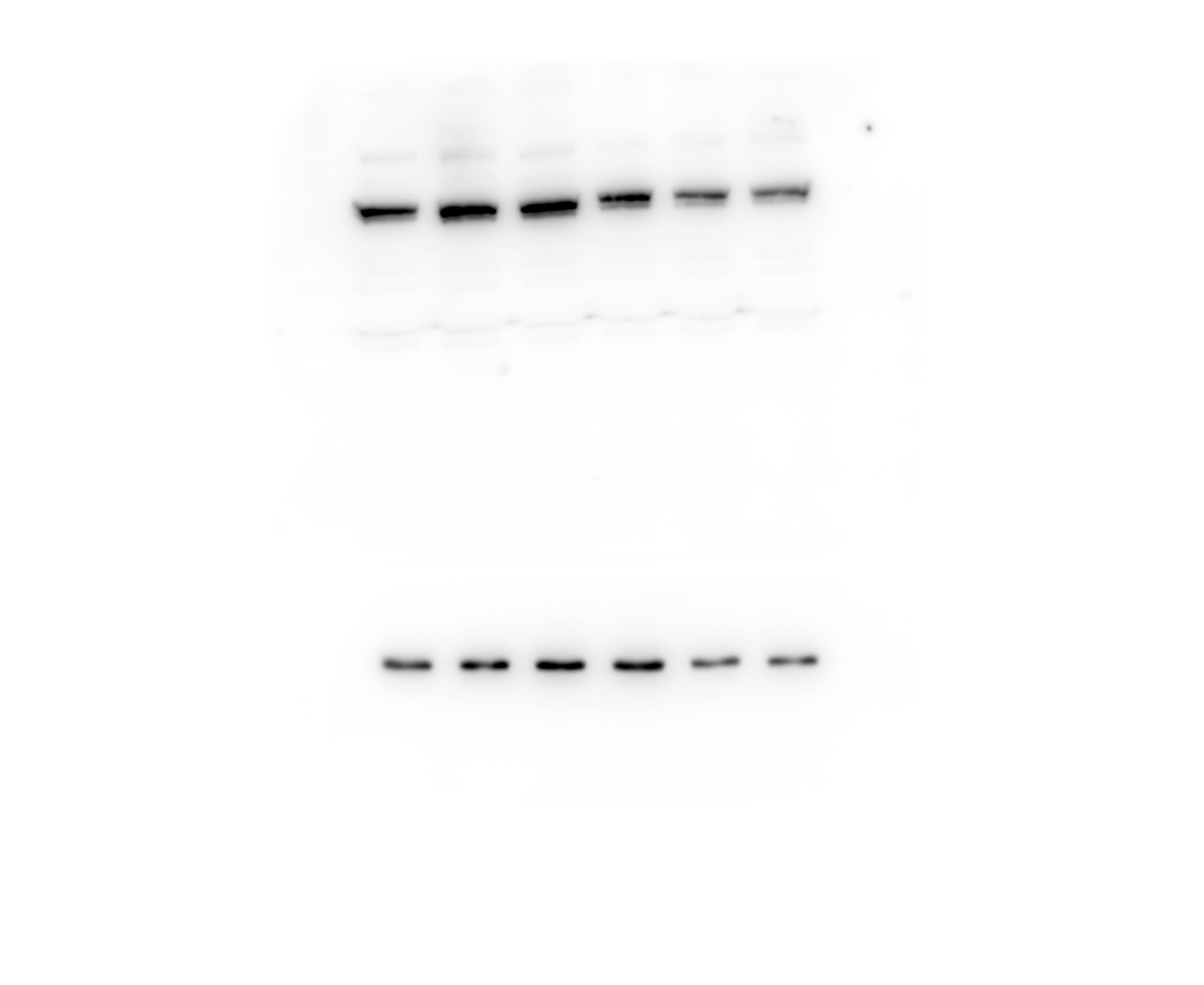

Supplement: Source data 1. [file elife-77755-data1.zip › Figure 2/Figure 2C alpha-tubulin.tif]

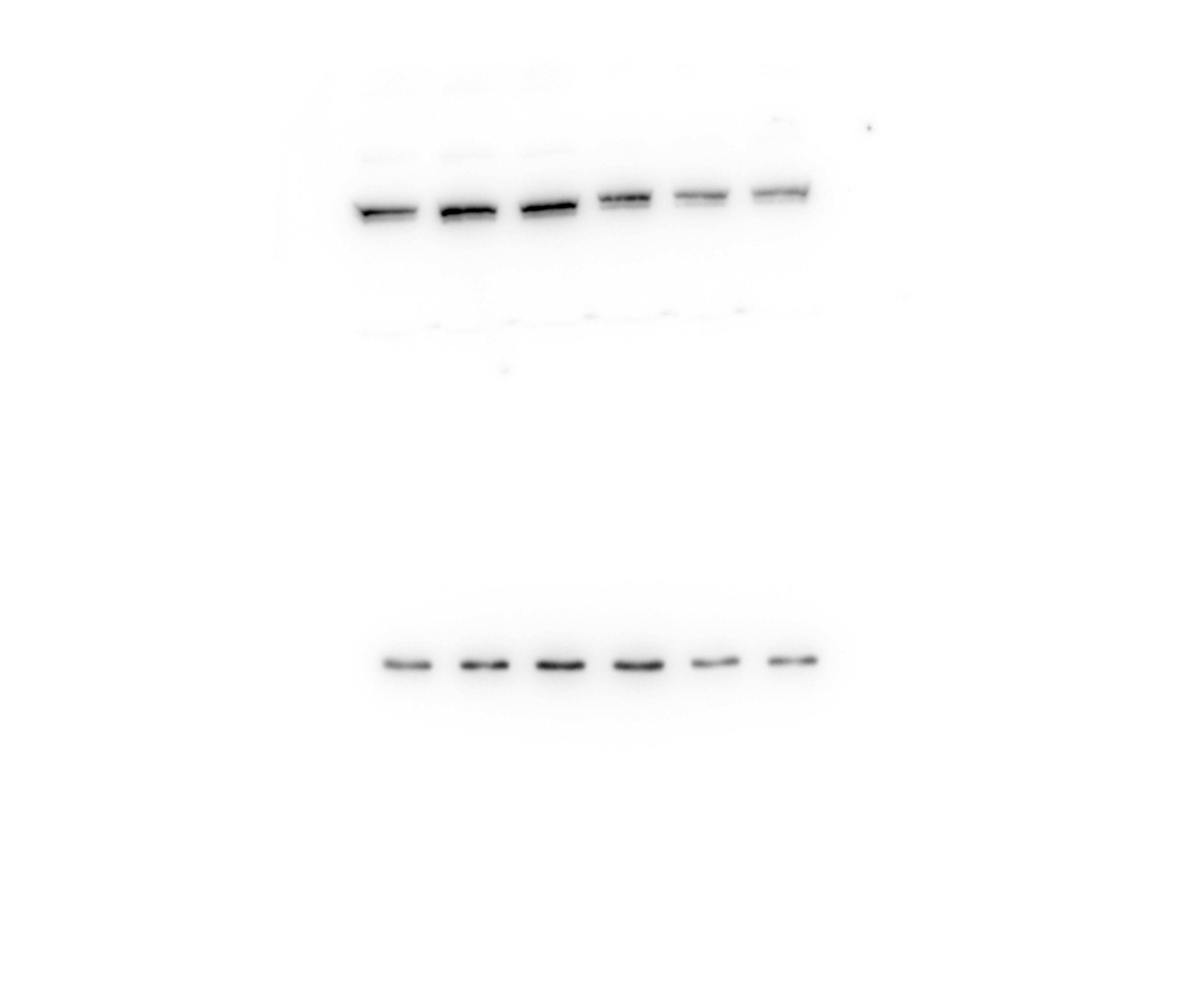

Supplement: Source data 1. [file elife-77755-data1.zip › Figure 2/Figure 2C Rai14.tif]

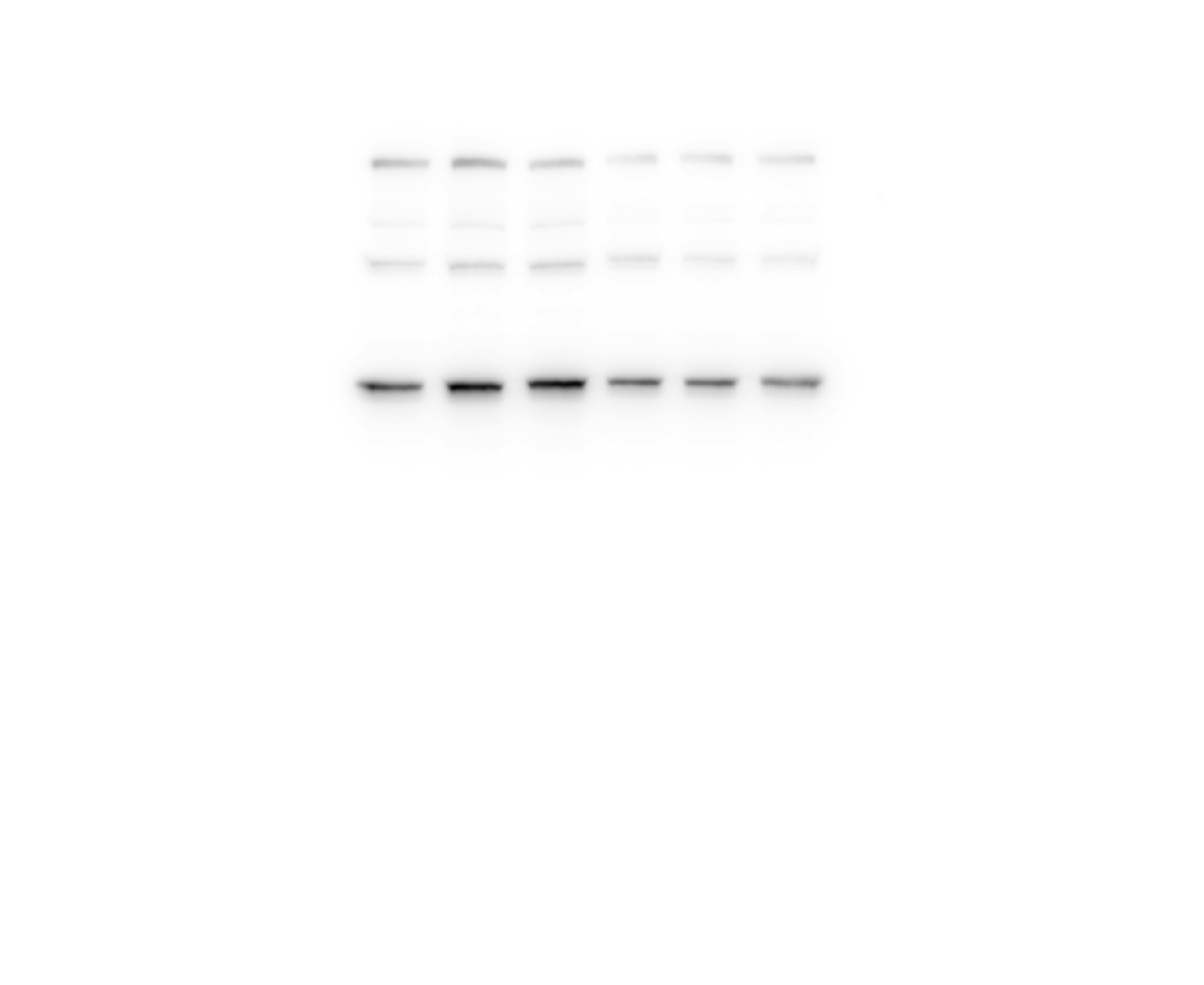

Supplement: Source data 1. [file elife-77755-data1.zip › Figure 2/Figure 2C Tara.tif]

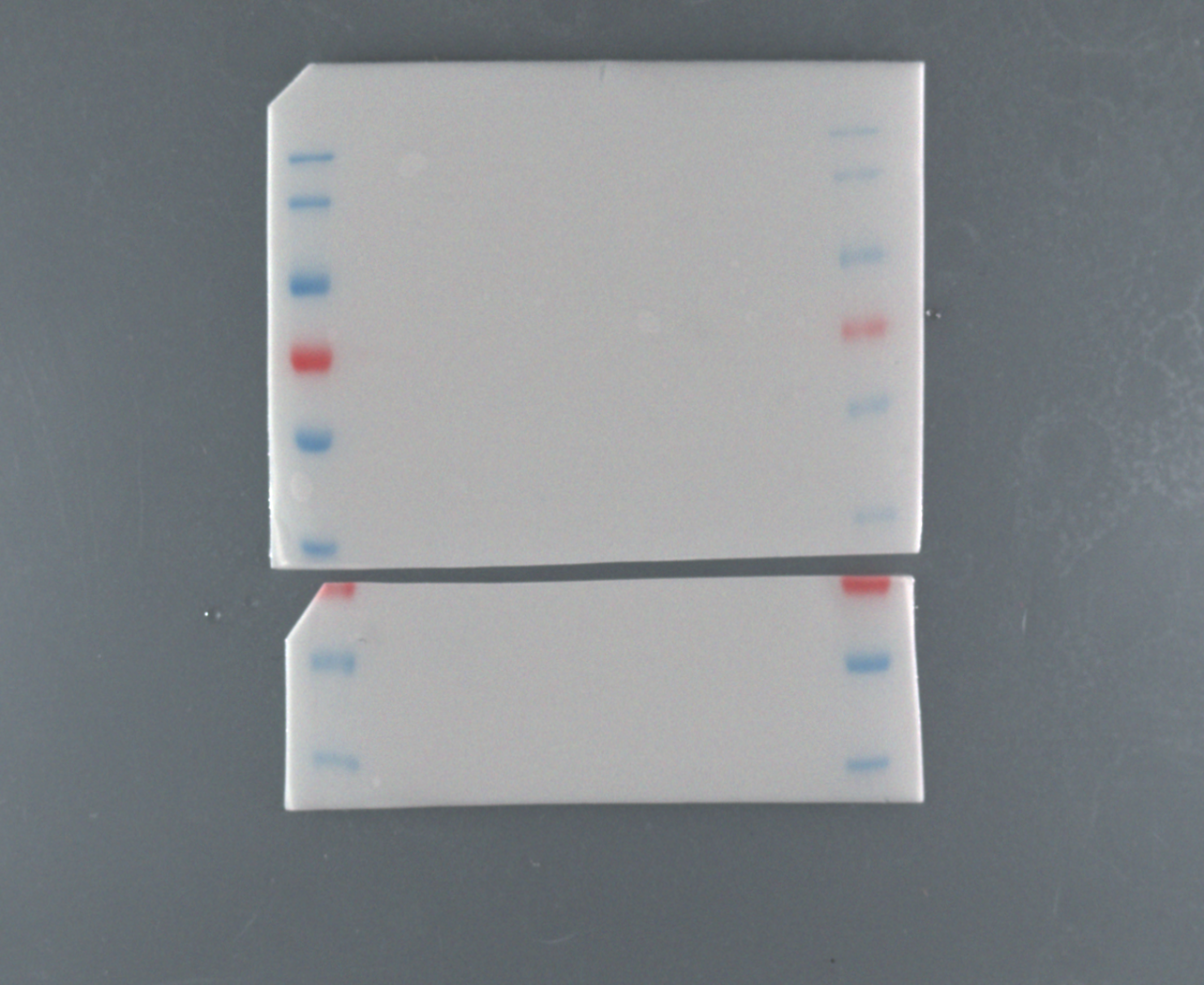

Supplement: Source data 1. [file elife-77755-data1.zip › Figure 2/Figure 2C-size marker for Rai14 and alpha-tubulin.tif]

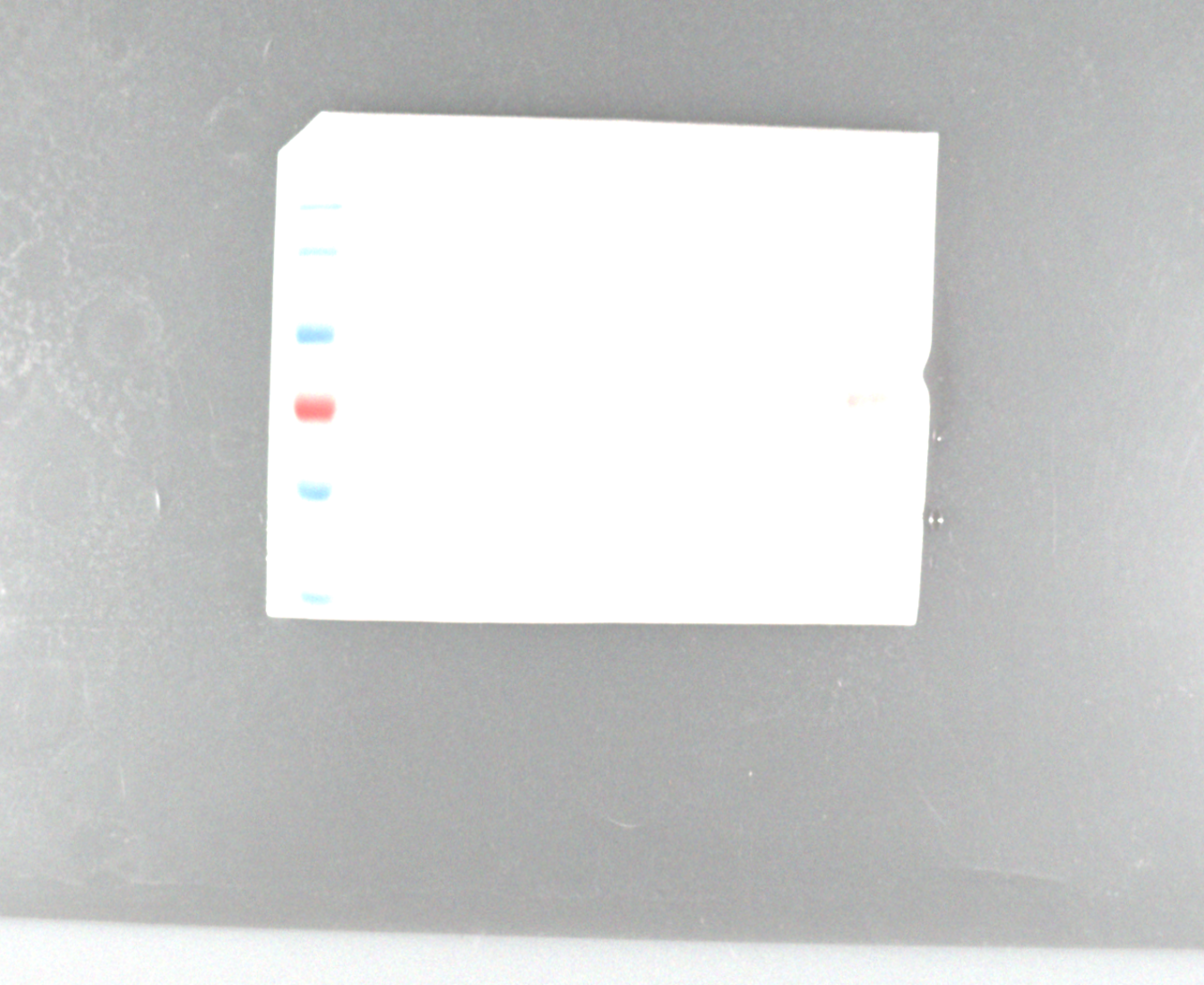

Supplement: Source data 1. [file elife-77755-data1.zip › Figure 2/Figure 2C-size marker for Tara.tif]

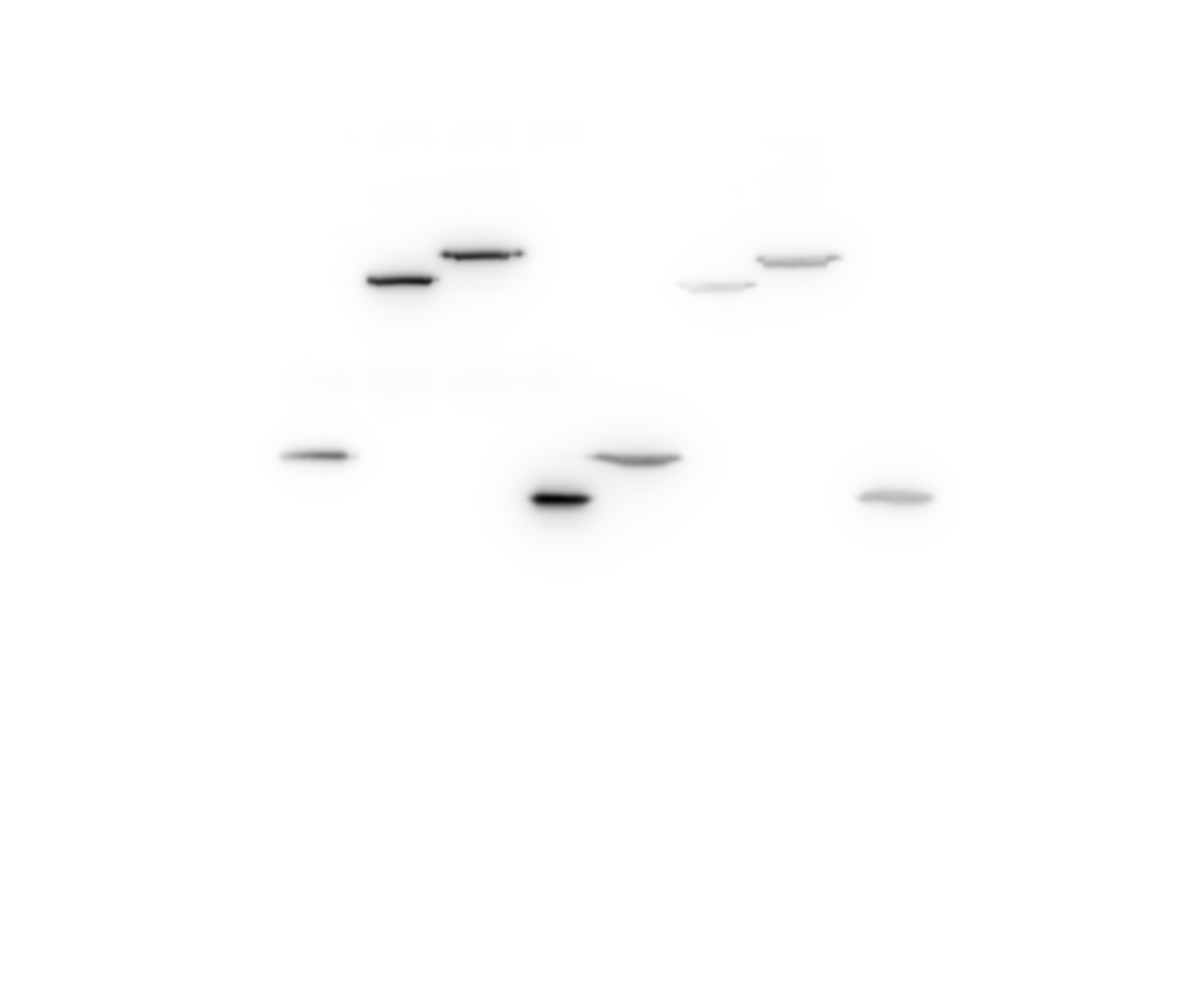

Supplement: Source data 1. [file elife-77755-data1.zip › Figure 2/Figure 2E GFP (Tara).tif]

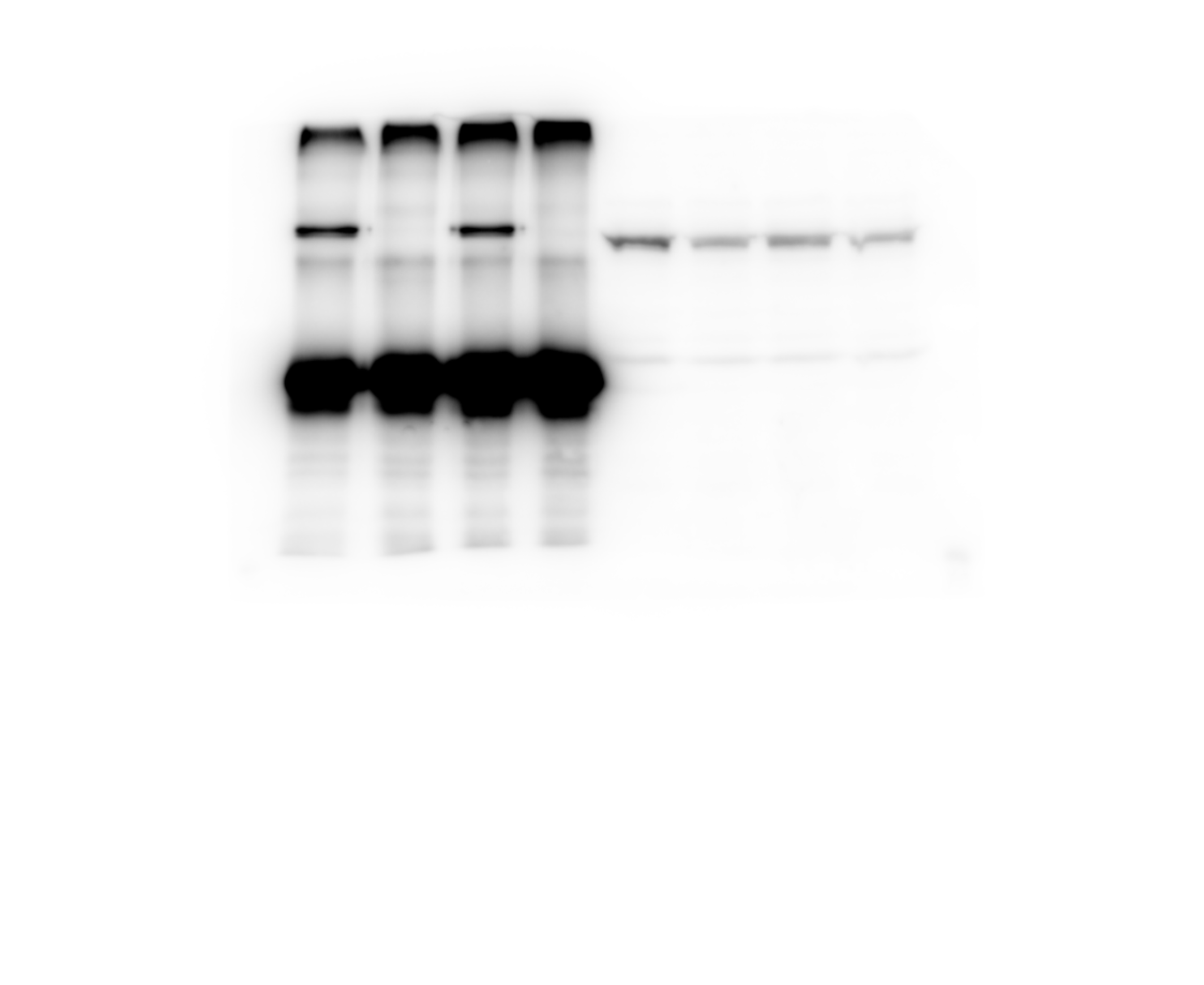

Supplement: Source data 1. [file elife-77755-data1.zip › Figure 2/Figure 2E Rai14.tif]

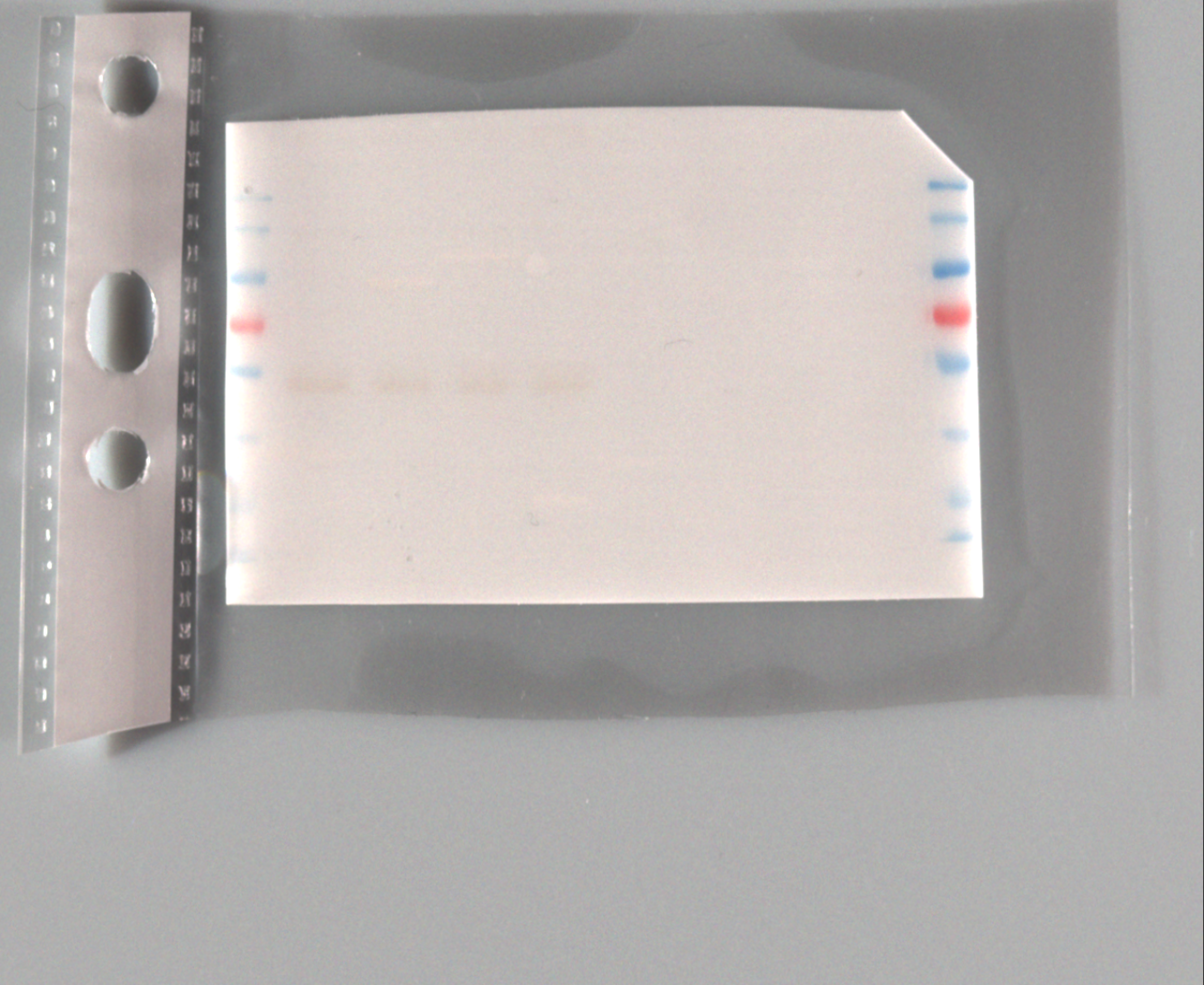

Supplement: Source data 1. [file elife-77755-data1.zip › Figure 2/Figure 2E-size marker for GFP (Tara).tif]

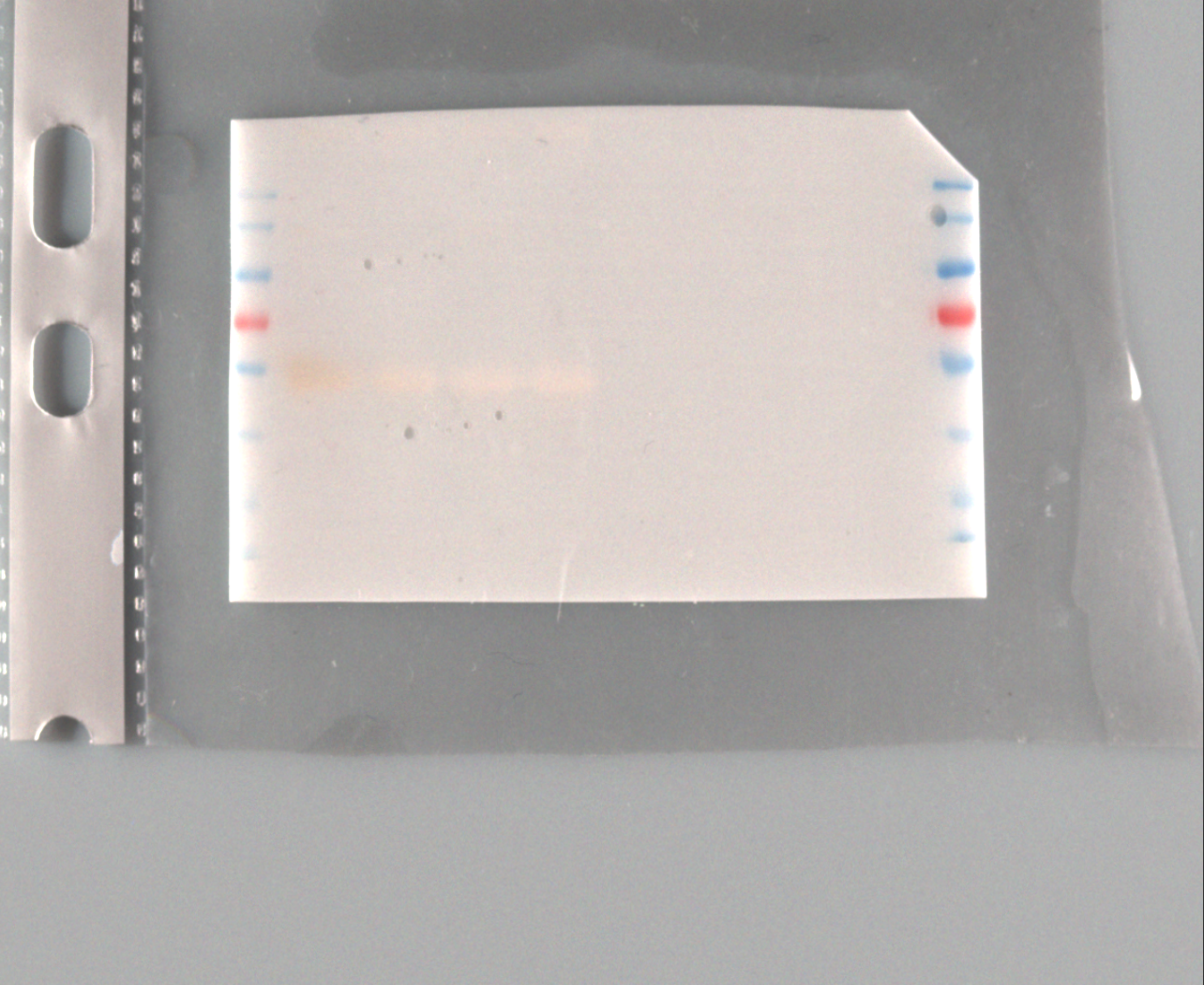

Supplement: Source data 1. [file elife-77755-data1.zip › Figure 2/Figure 2E-size marker for Rai14.tif]

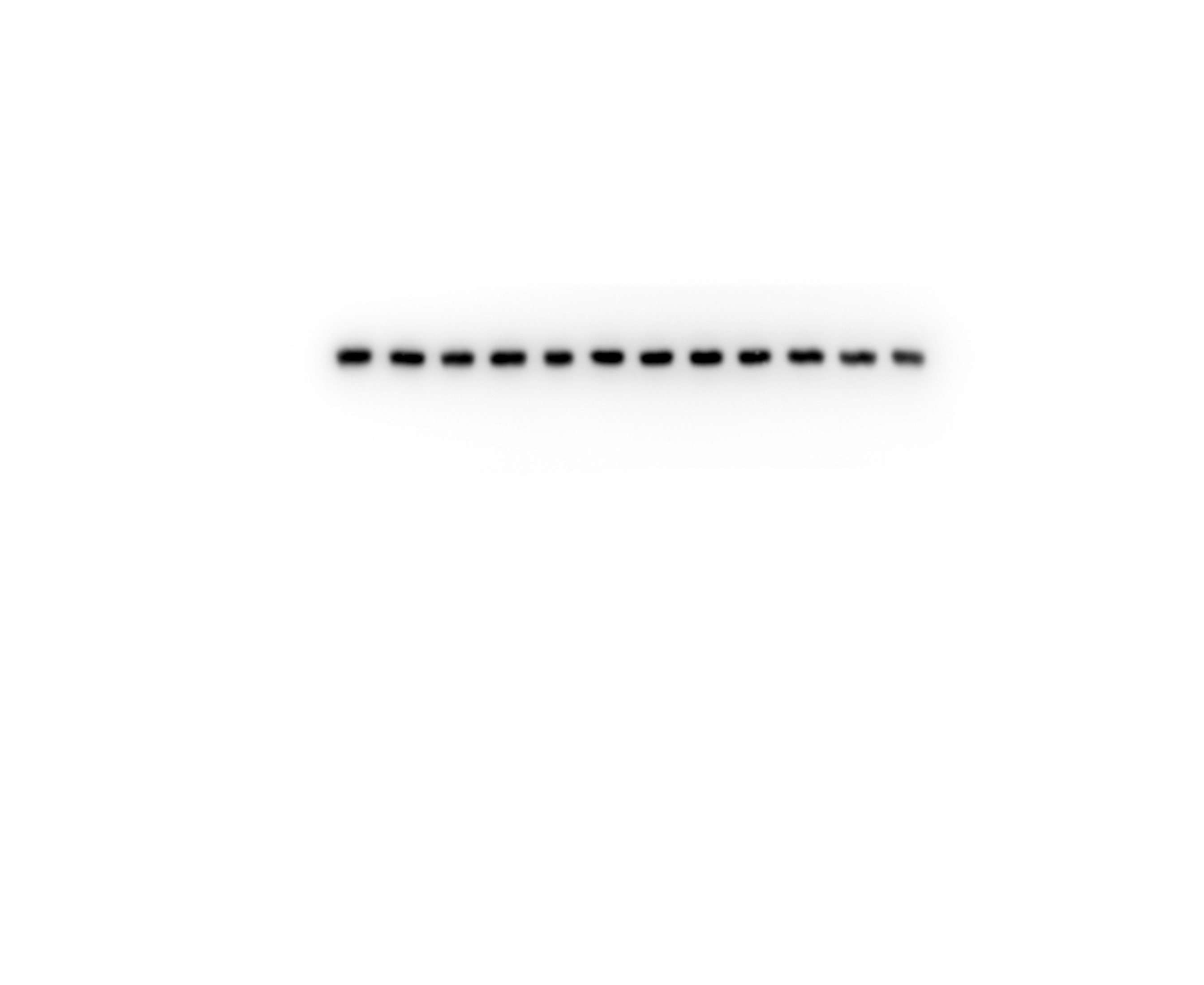

Supplement: Source data 1. [file elife-77755-data1.zip › Figure 2/Figure 2F alpha-tubulin.tif]

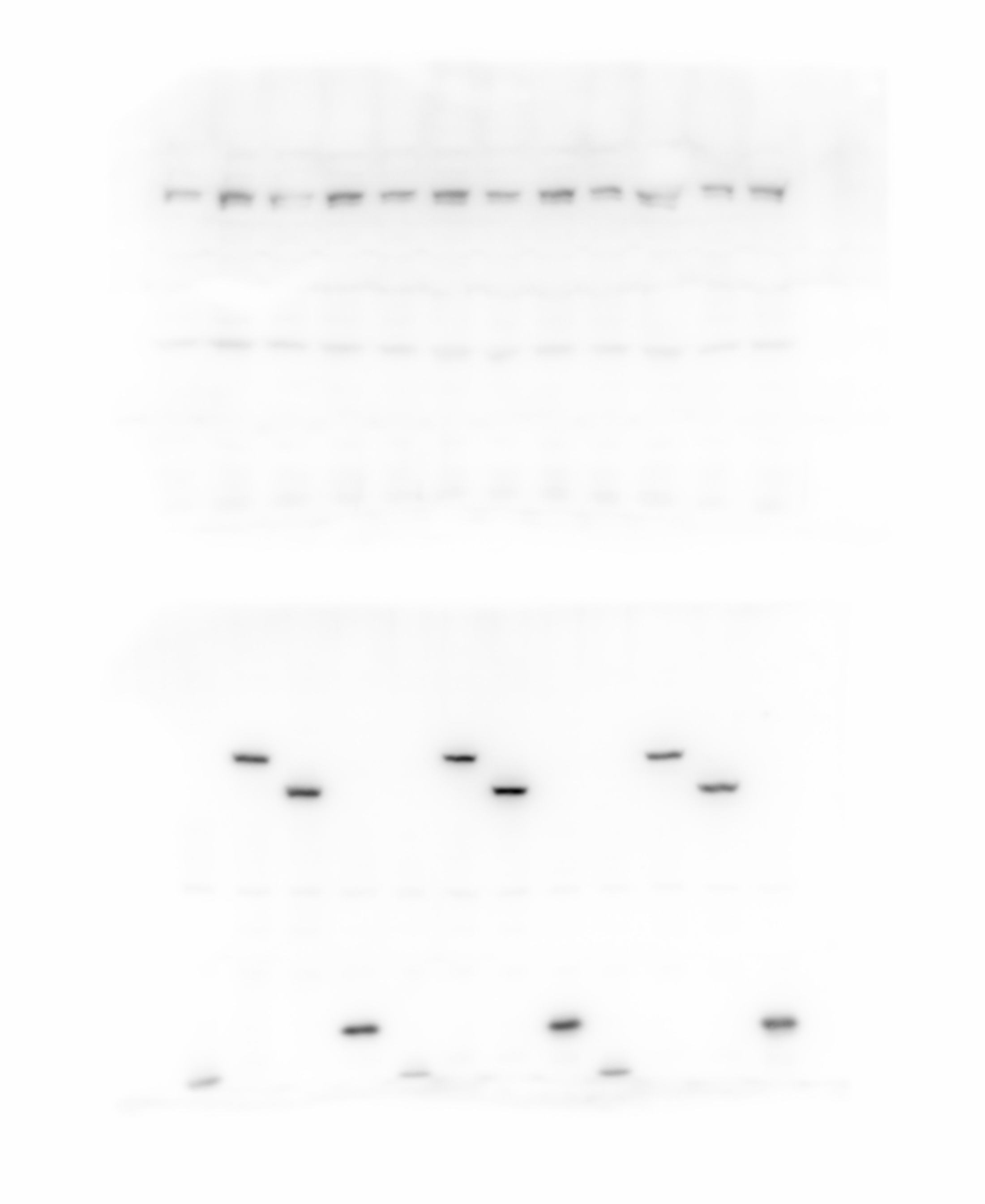

Supplement: Source data 1. [file elife-77755-data1.zip › Figure 2/Figure 2F GFP (Tara).tif]

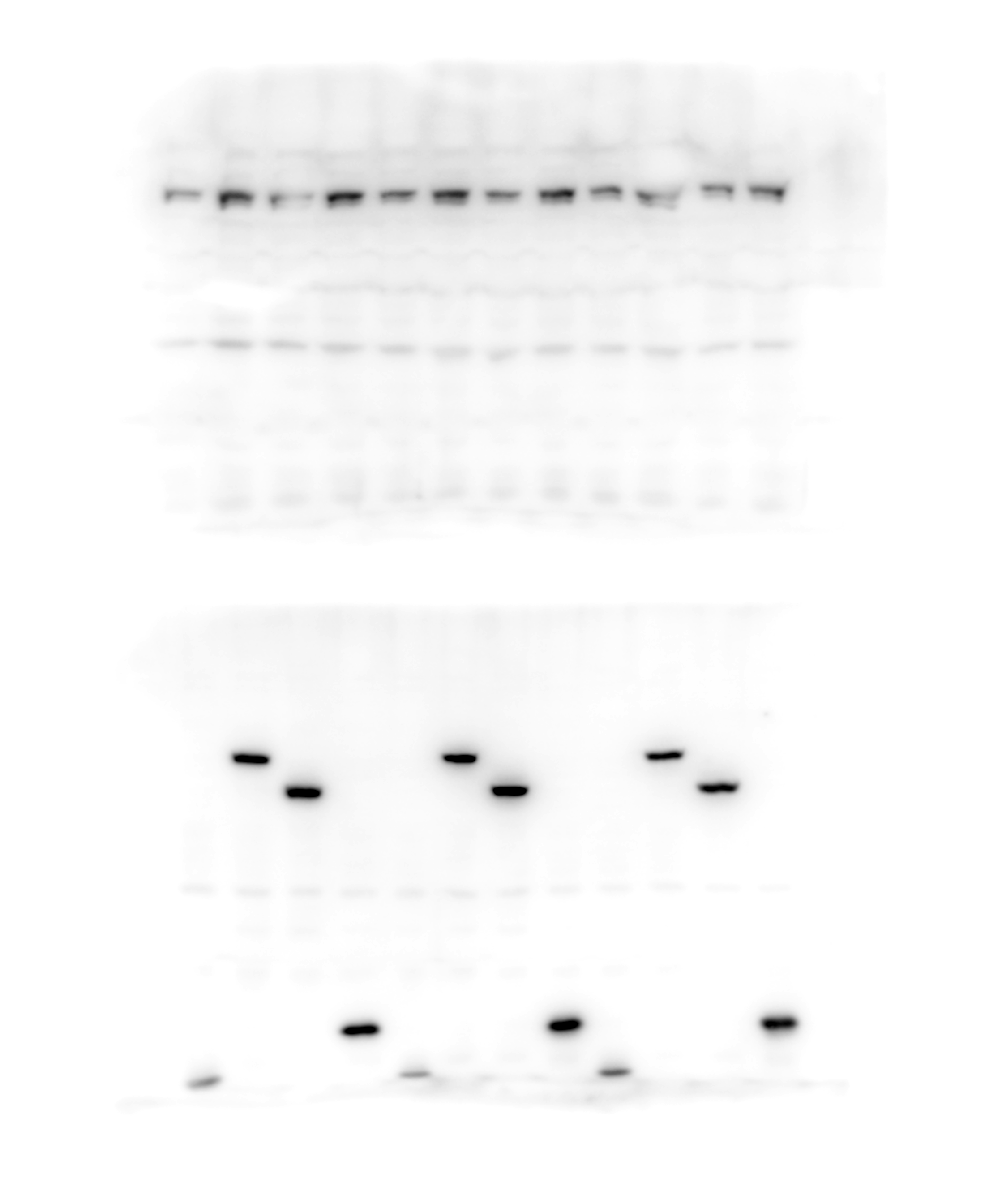

Supplement: Source data 1. [file elife-77755-data1.zip › Figure 2/Figure 2F Rai14.tif]

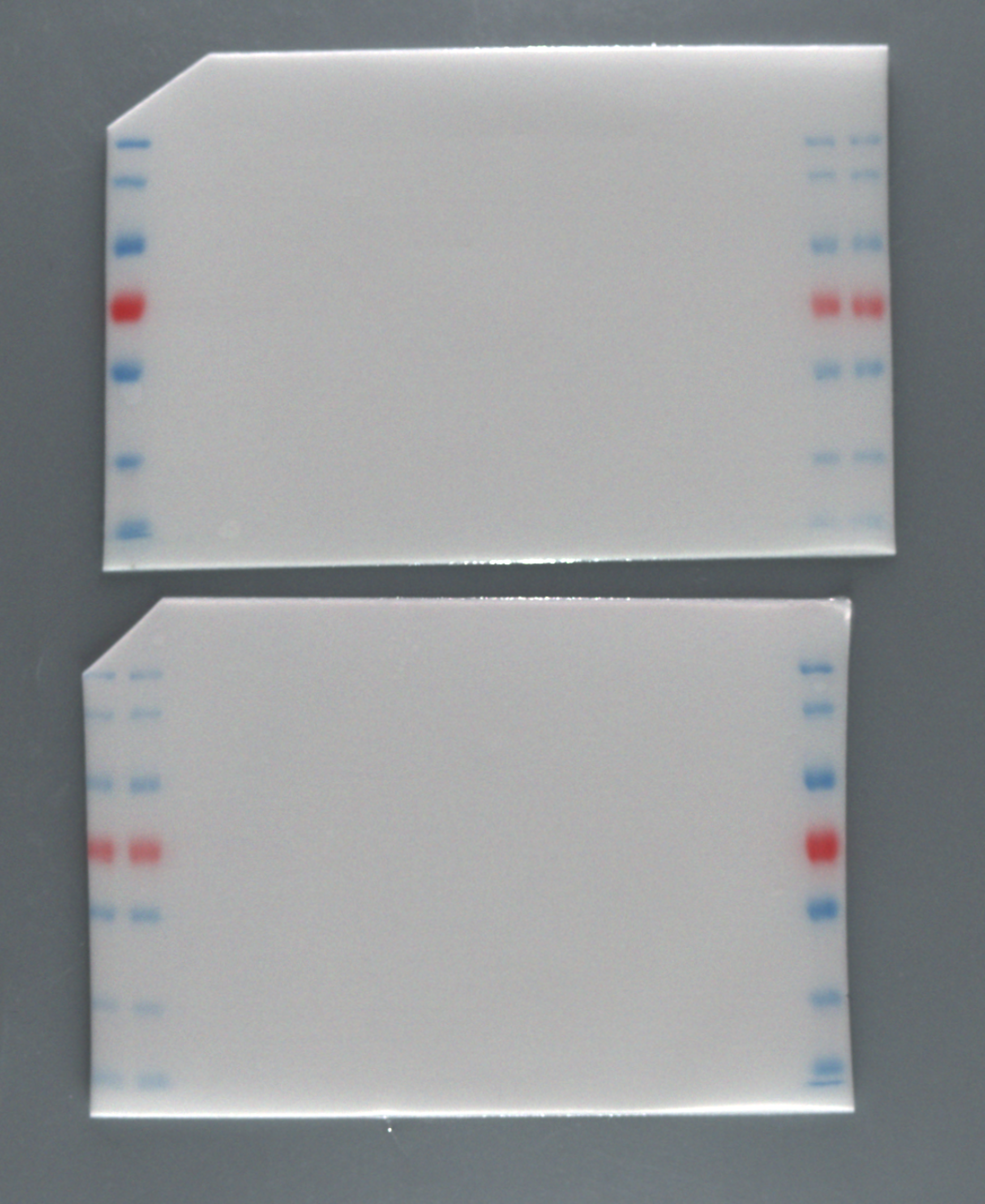

Supplement: Source data 1. [file elife-77755-data1.zip › Figure 2/Figure 2F-size marker for Rai14, GFP (Tara) and alpha-tubulin.tif.tif]

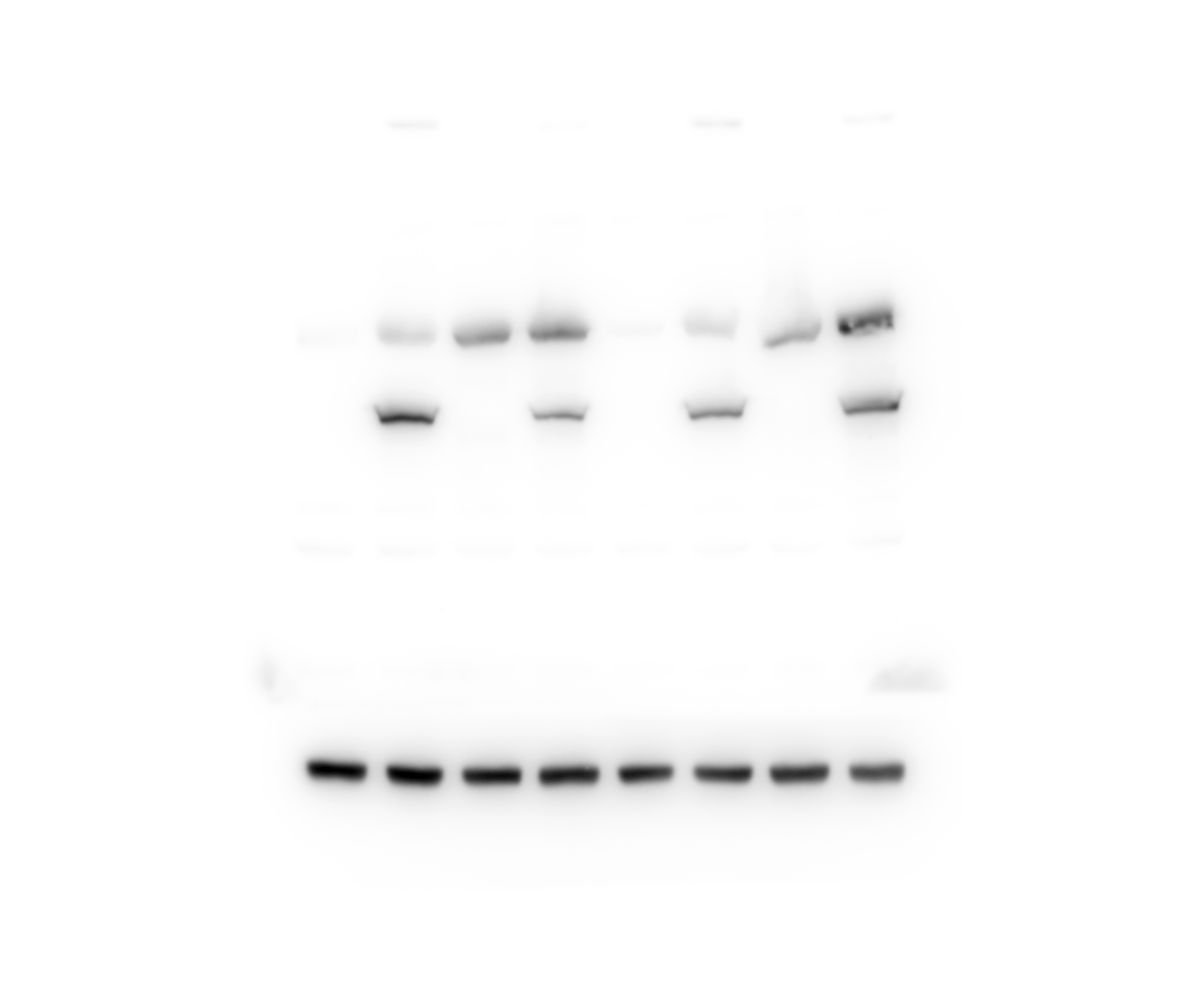

Supplement: Source data 1. [file elife-77755-data1.zip › Figure 2/Figure 2G alpha-tubulin.tif]

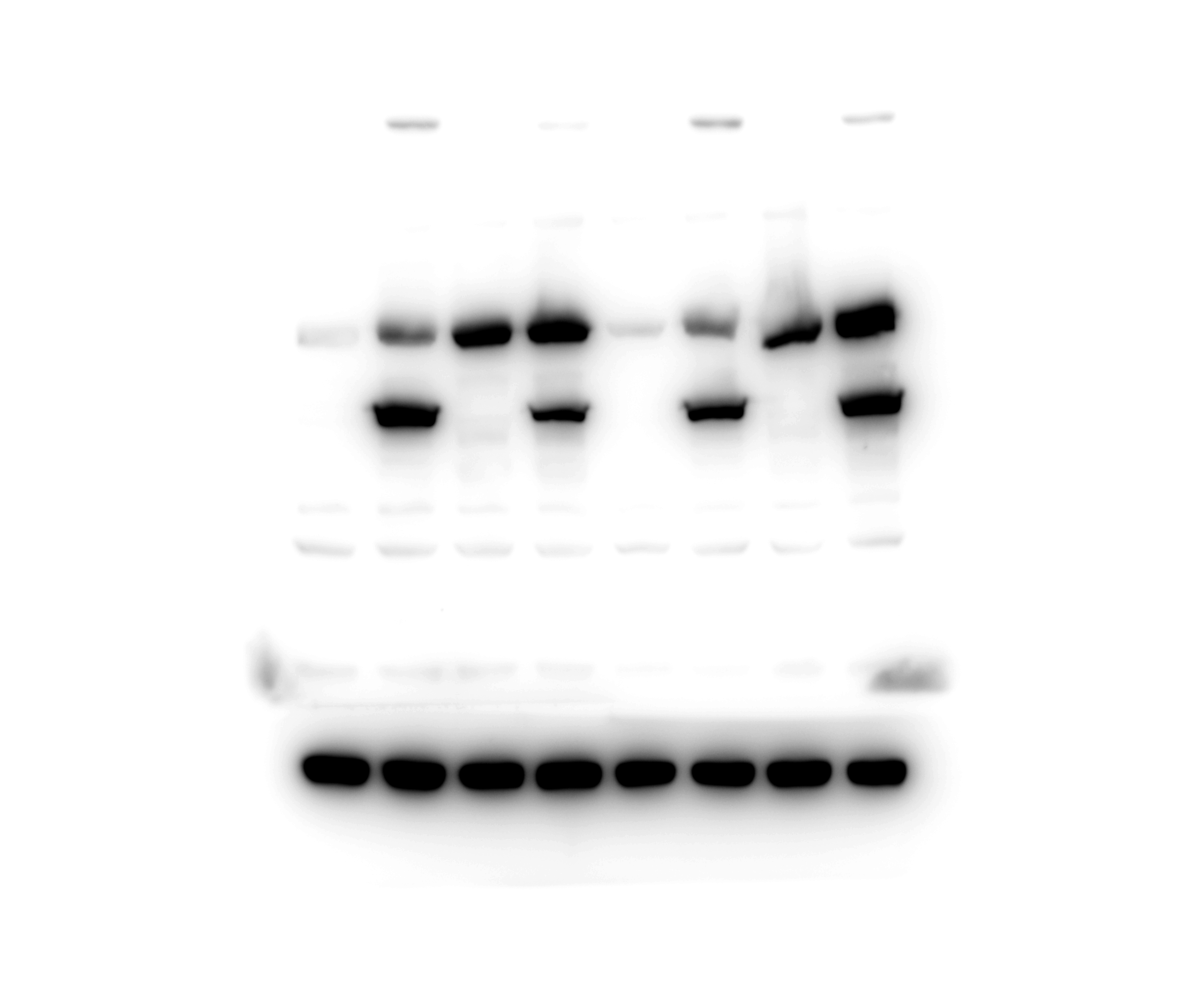

Supplement: Source data 1. [file elife-77755-data1.zip › Figure 2/Figure 2G GFP (Rai14).tif]

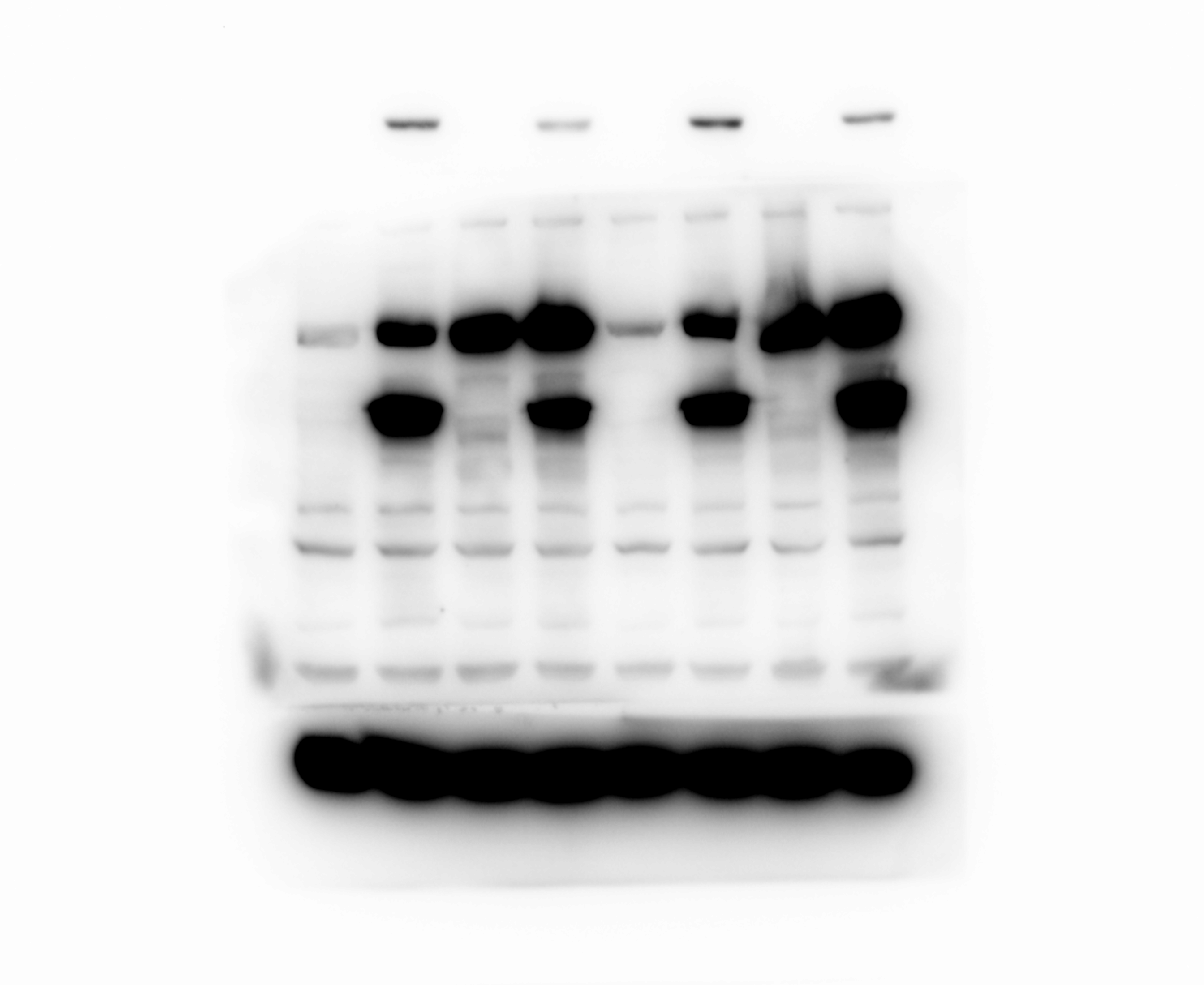

Supplement: Source data 1. [file elife-77755-data1.zip › Figure 2/Figure 2G Myc (Tara).tif]

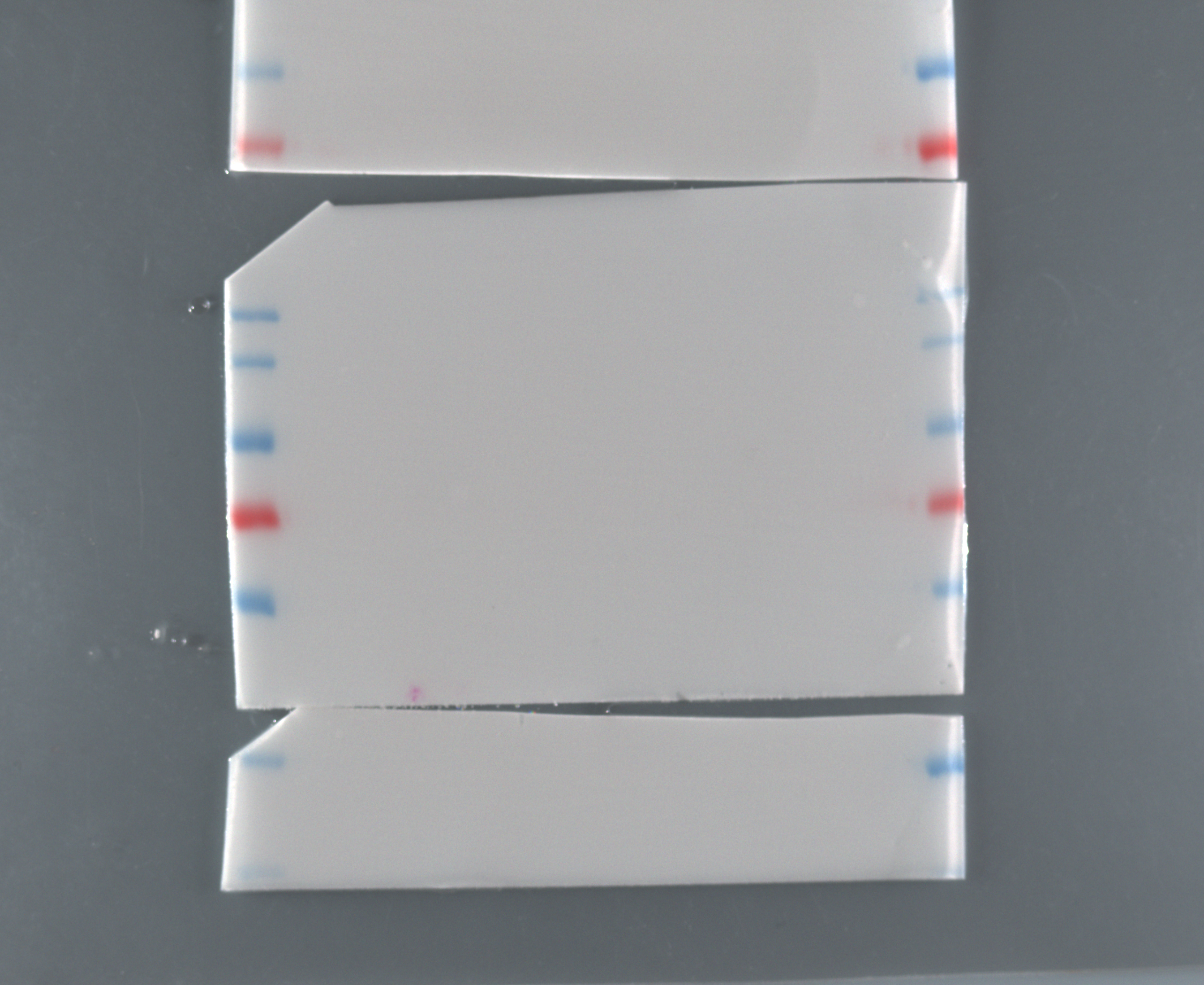

Supplement: Source data 1. [file elife-77755-data1.zip › Figure 2/Figure 2G-size marker for GFP (Rai14), Myc (Tara) and alpha-tubulin.tif]

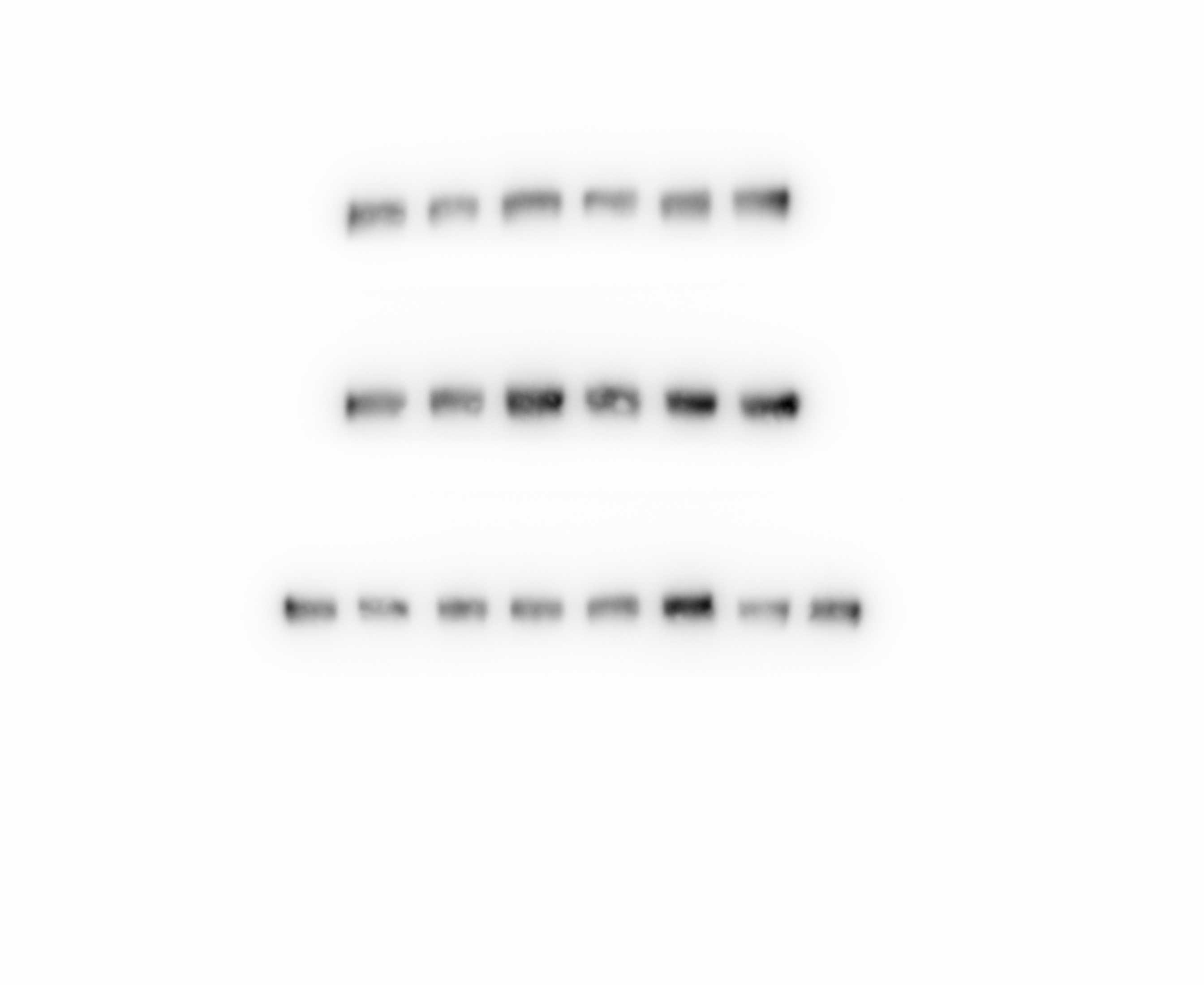

Supplement: Source data 1. [file elife-77755-data1.zip › Figure 5/Figure 5K alpha-tubulin.tif]

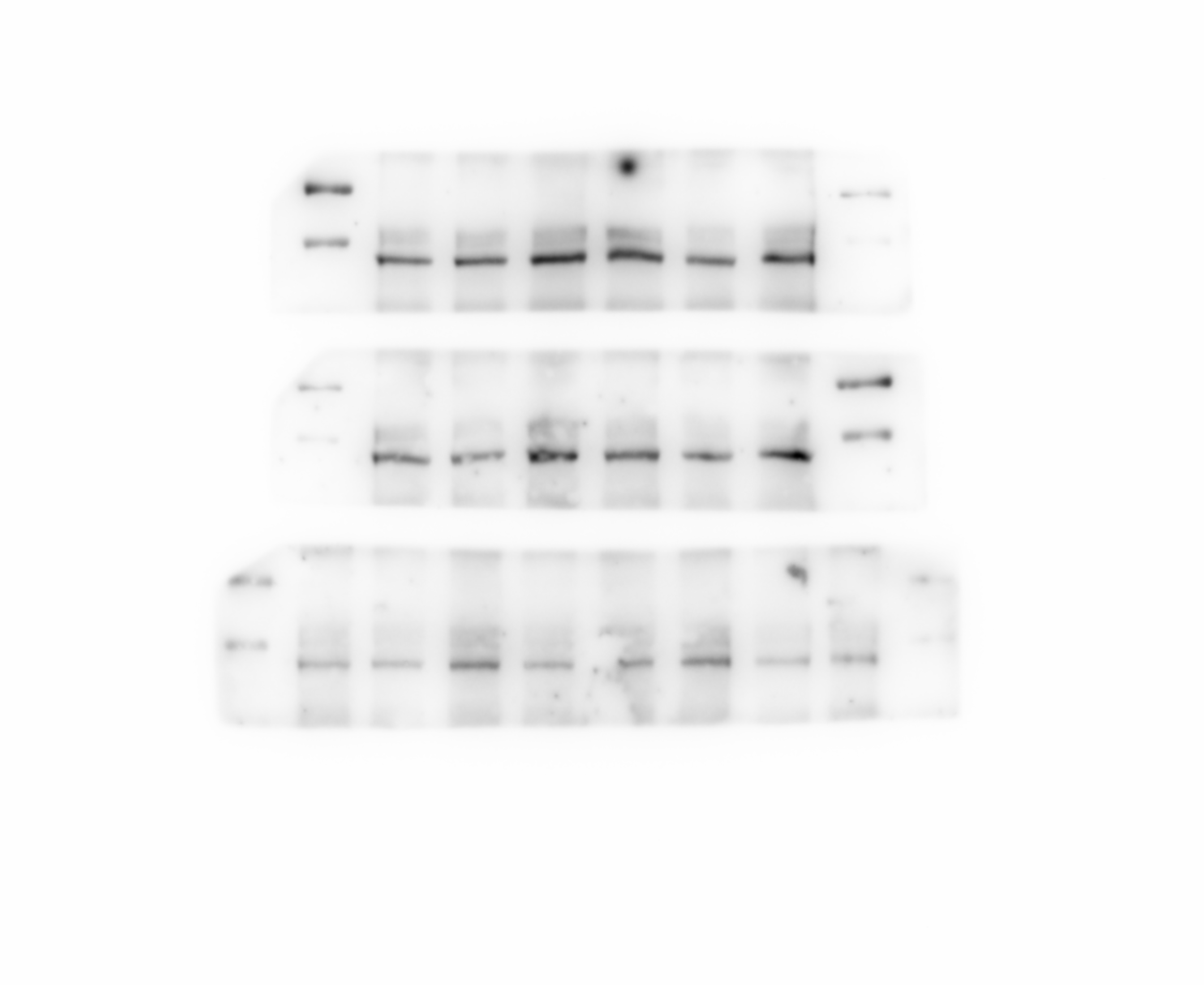

Supplement: Source data 1. [file elife-77755-data1.zip › Figure 5/Figure 5K Rai14.tif]

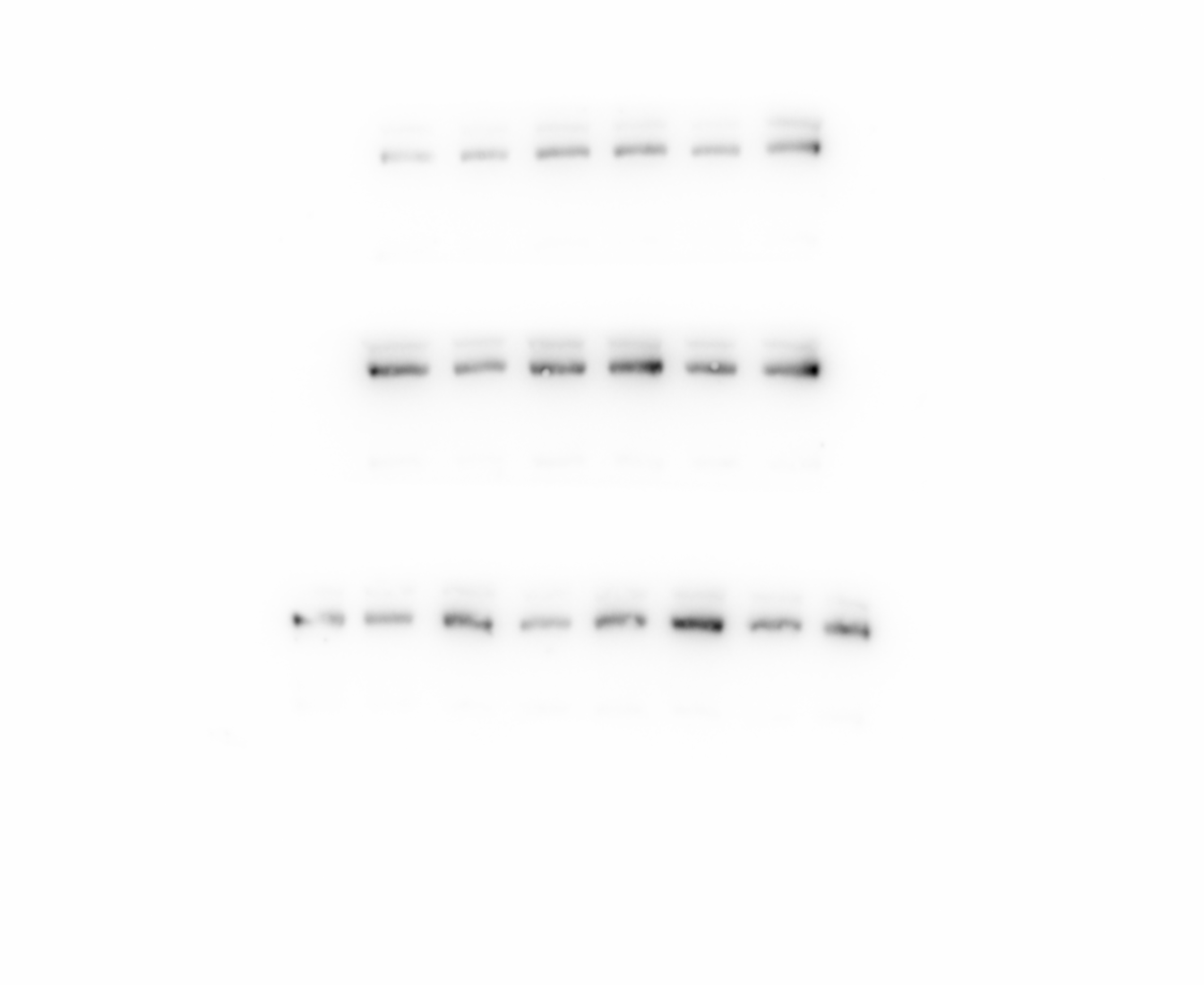

Supplement: Source data 1. [file elife-77755-data1.zip › Figure 5/Figure 5K Tara.tif]

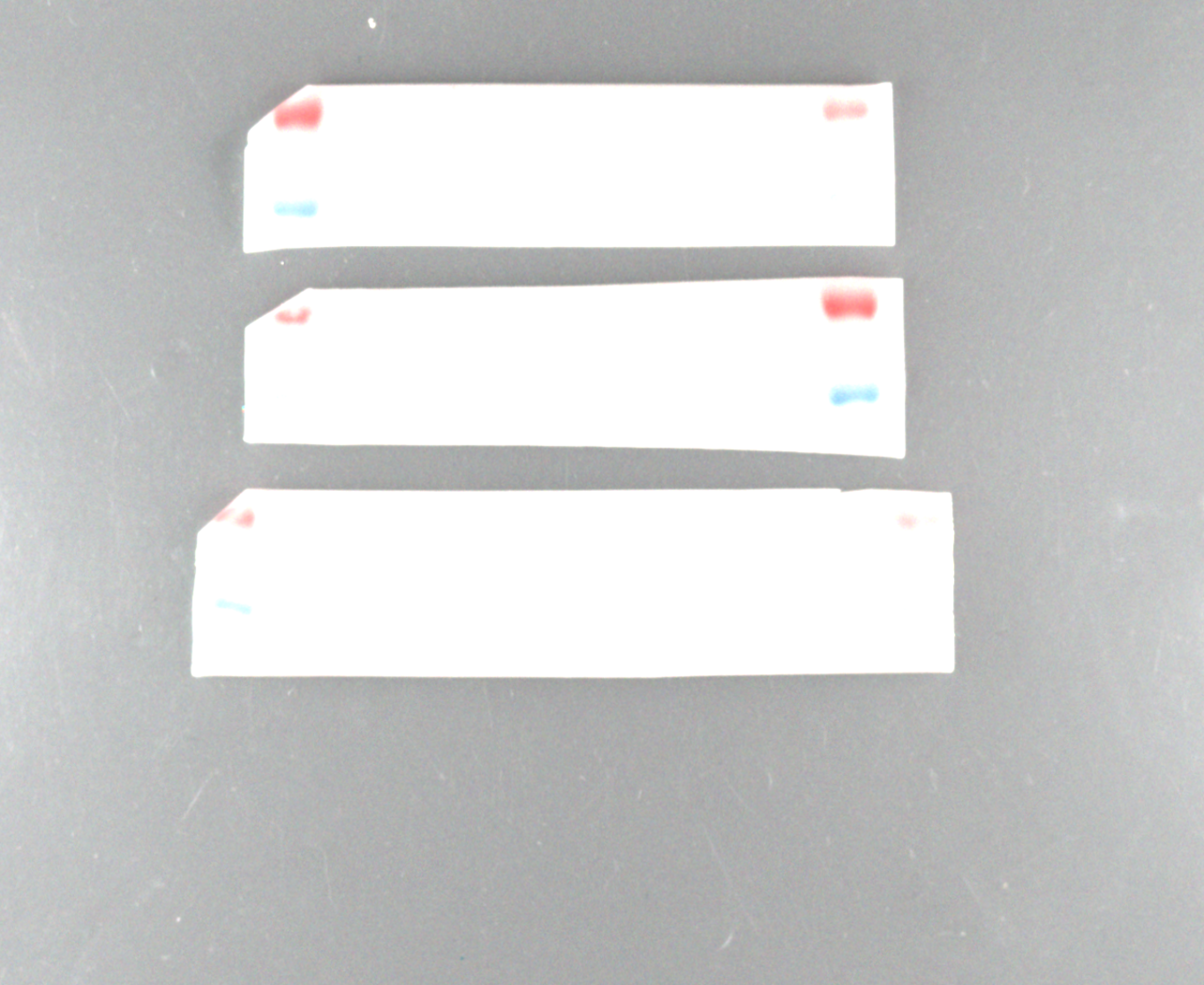

Supplement: Source data 1. [file elife-77755-data1.zip › Figure 5/Figure 5K-size marker for alpha-tubulin.tif]

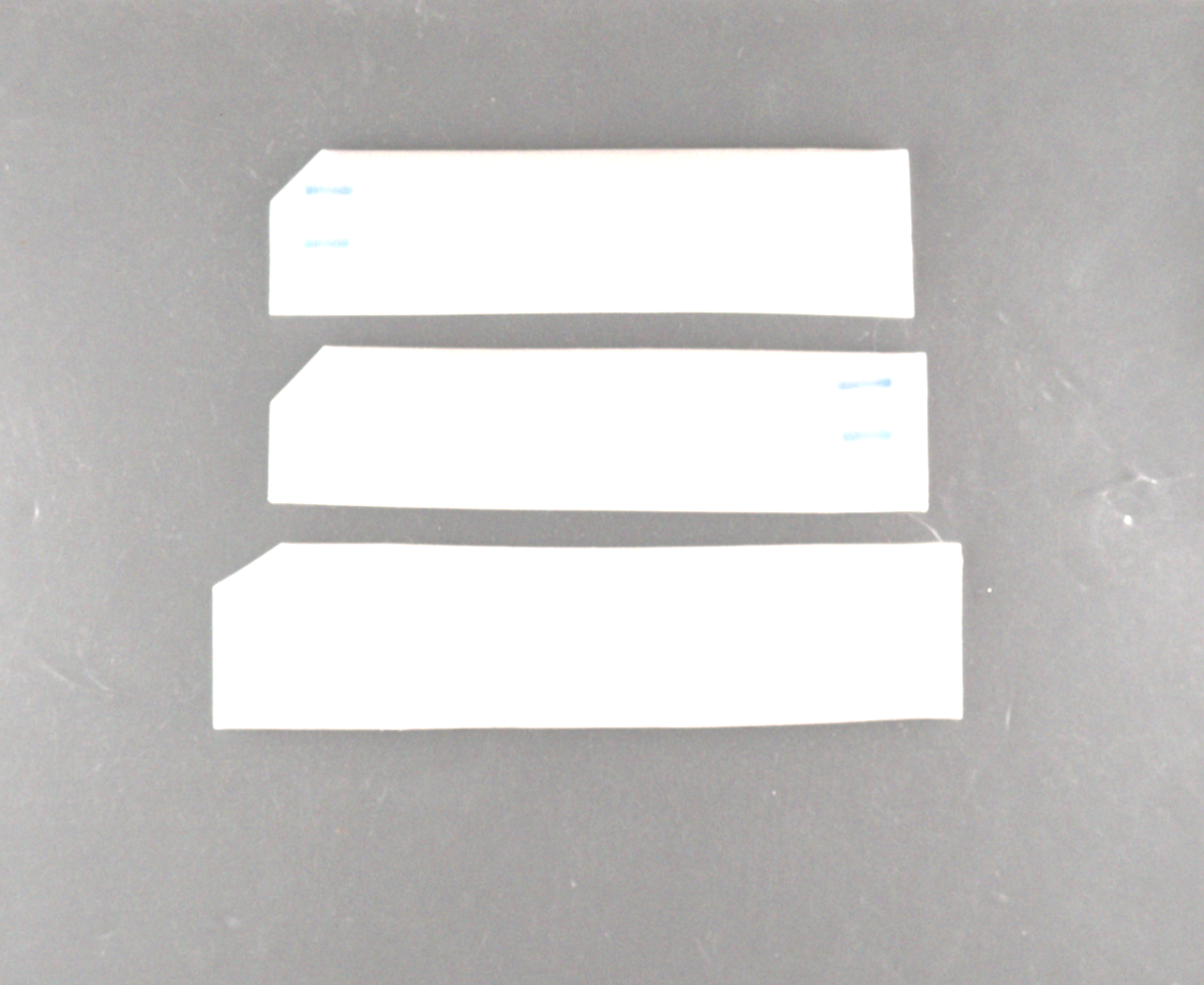

Supplement: Source data 1. [file elife-77755-data1.zip › Figure 5/Figure 5K-size marker for Rai14.tif]

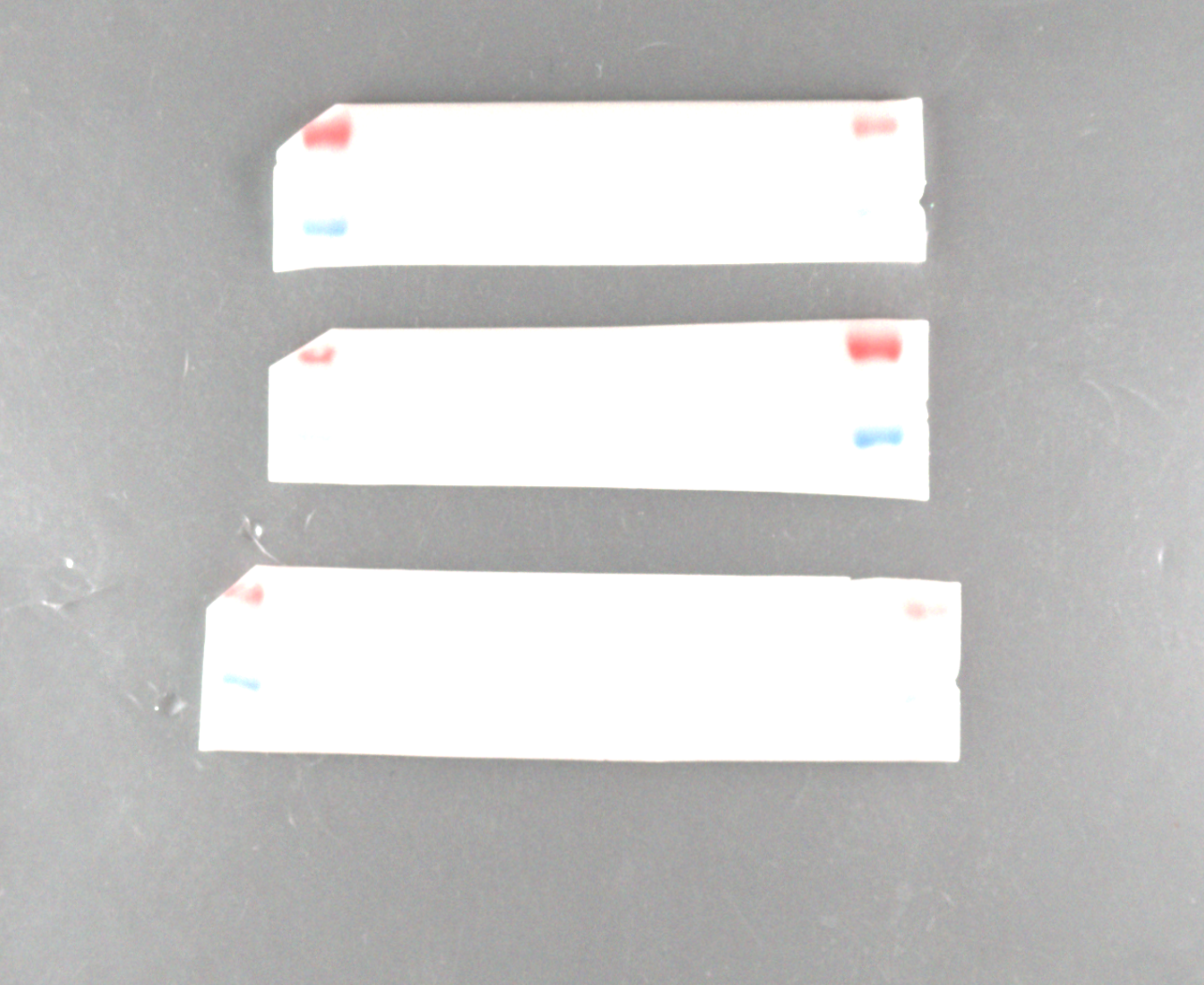

Supplement: Source data 1. [file elife-77755-data1.zip › Figure 5/Figure 5K-size marker for Tara.tif]

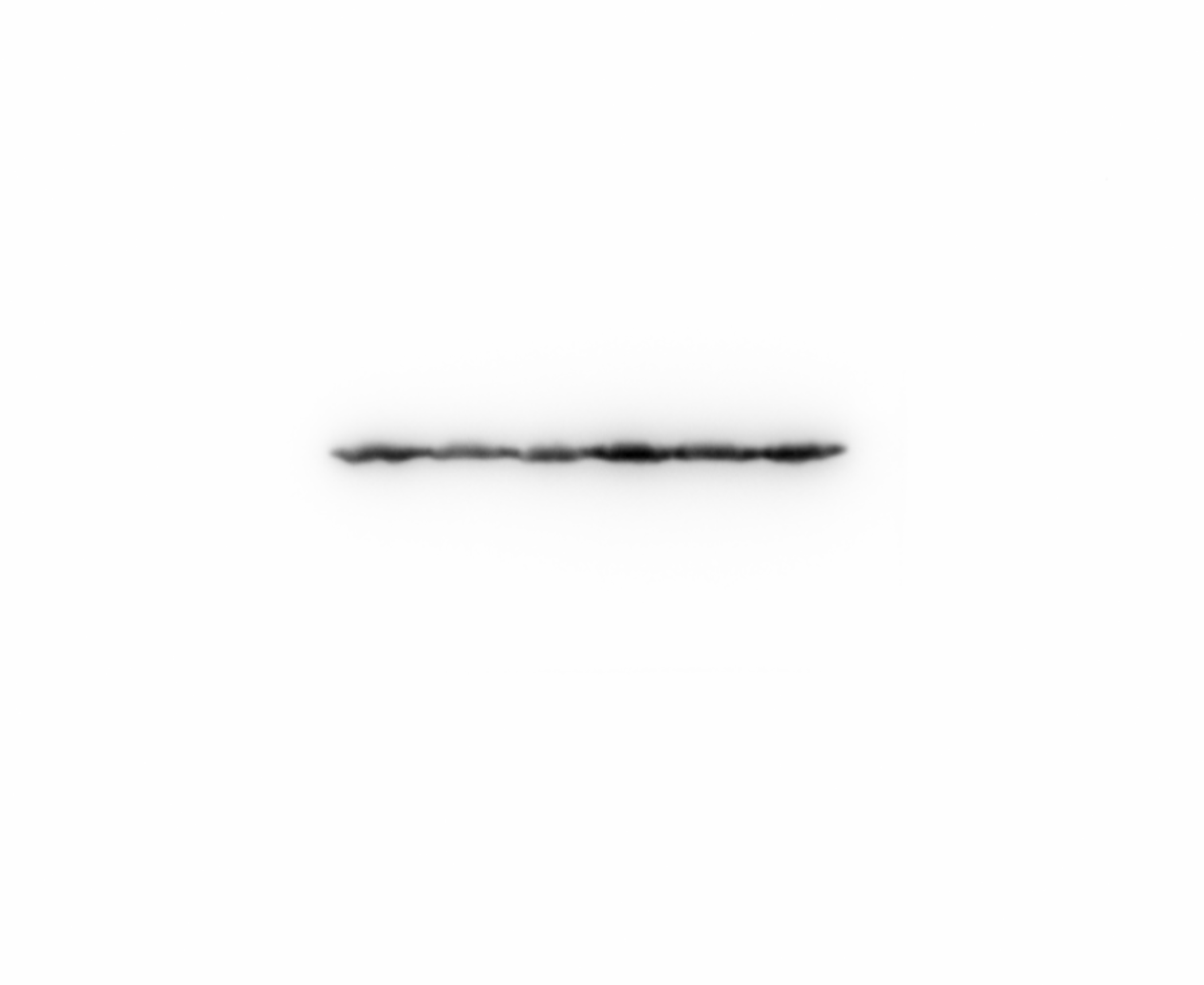

Supplement: Source data 2. [file elife-77755-data2.zip › Figure1-figure supplement1/Fig1-S1E alpha-tubulin.tif]

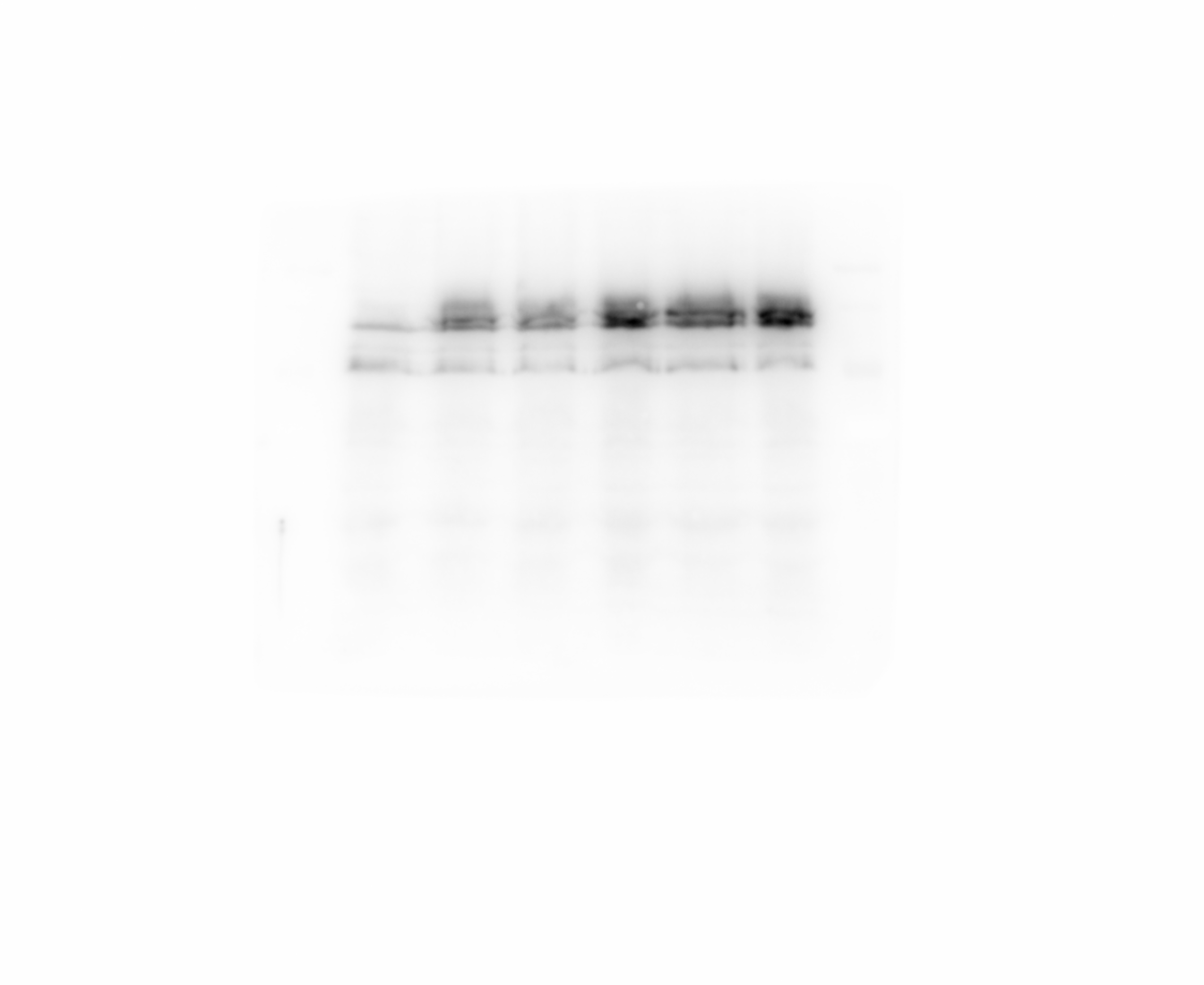

Supplement: Source data 2. [file elife-77755-data2.zip › Figure1-figure supplement1/Fig1-S1E Rai14.tif]

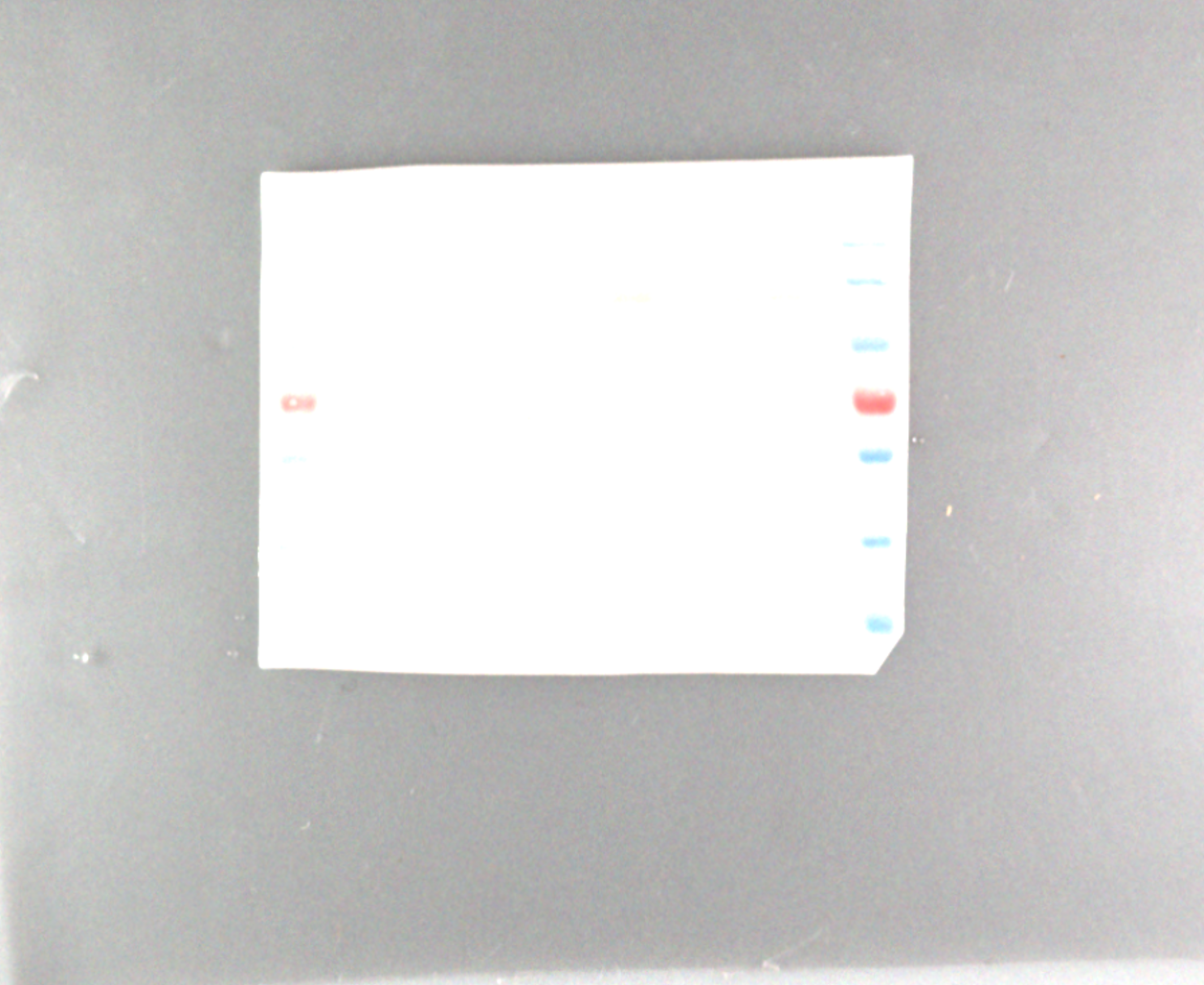

Supplement: Source data 2. [file elife-77755-data2.zip › Figure1-figure supplement1/Fig1-S1E-size marker for alpha-tubulin.tif]

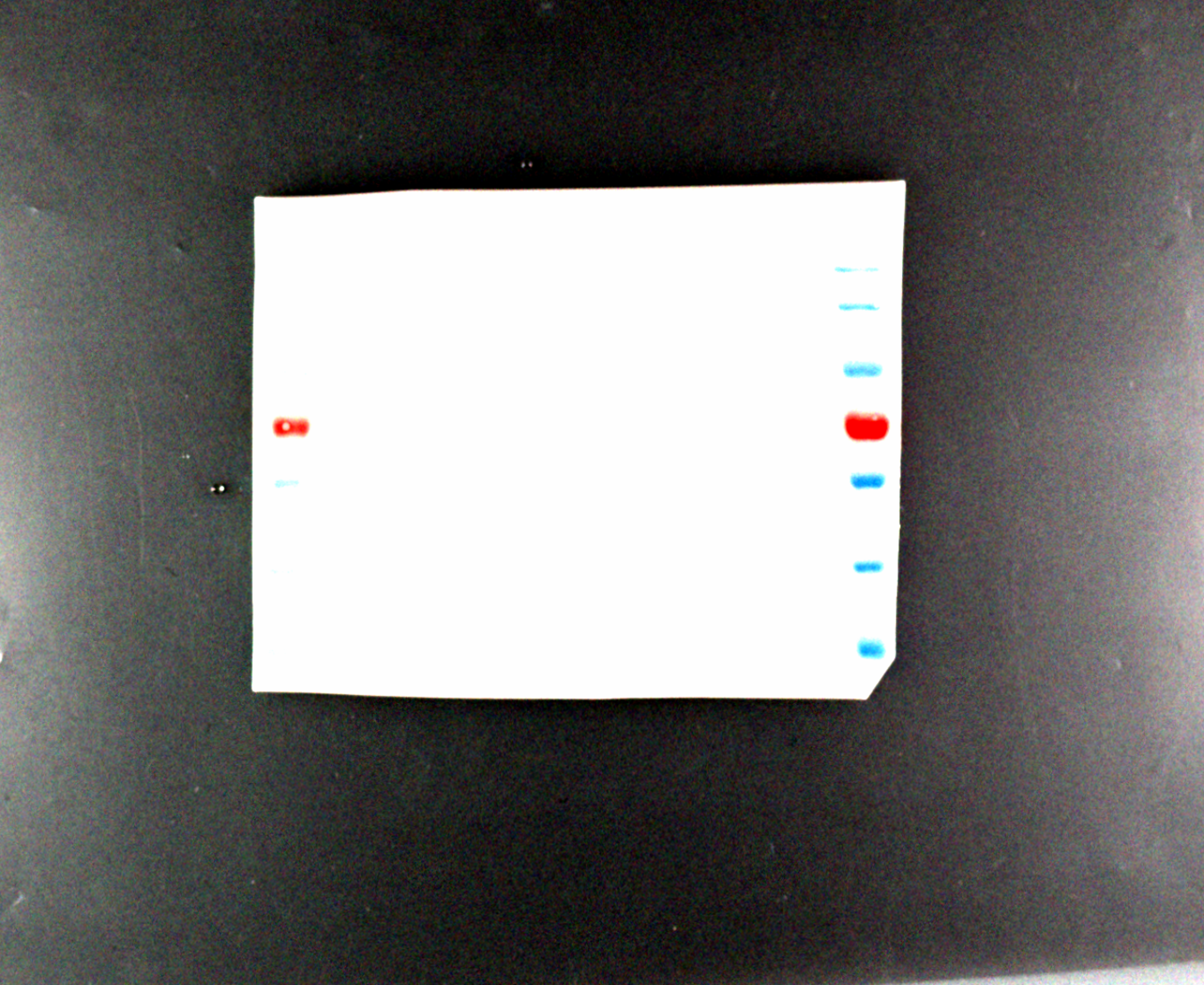

Supplement: Source data 2. [file elife-77755-data2.zip › Figure1-figure supplement1/Fig1-S1E-size marker for Rai14.tif]

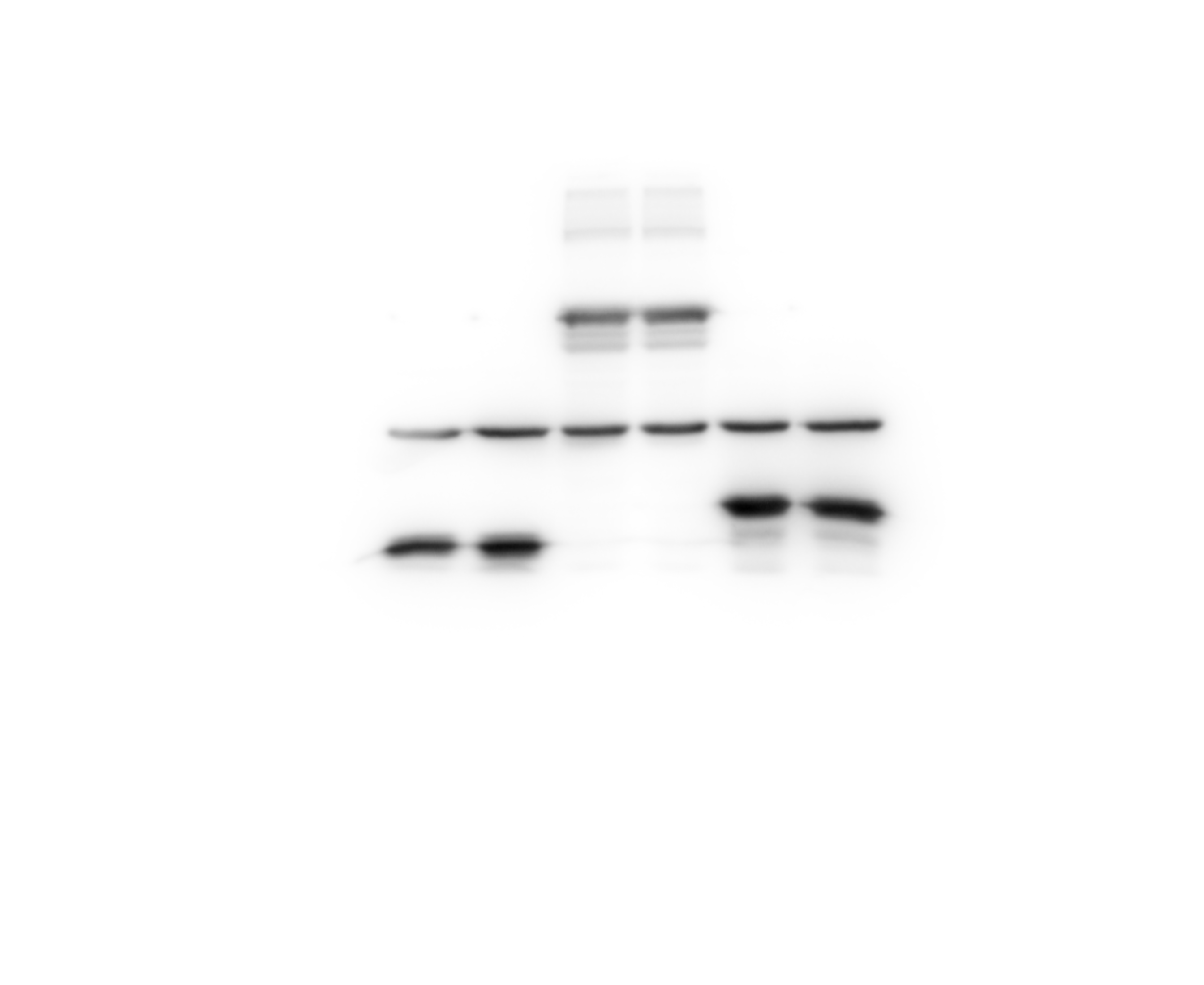

Supplement: Source data 2. [file elife-77755-data2.zip › Figure2-figure supplement1/Fig2-S1B alpha-tubulin.tif]

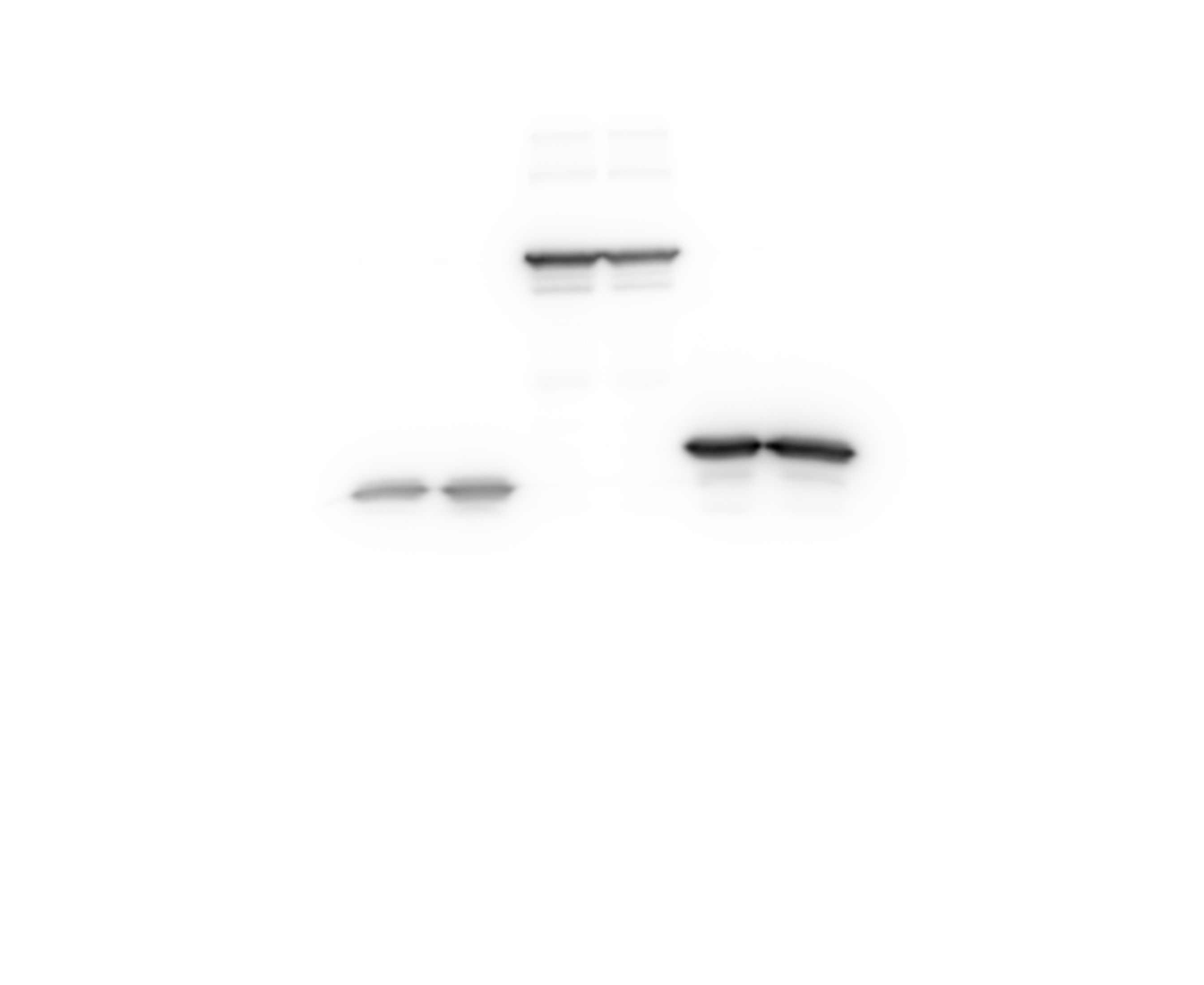

Supplement: Source data 2. [file elife-77755-data2.zip › Figure2-figure supplement1/Fig2-S1B GFP (Tara).tif]

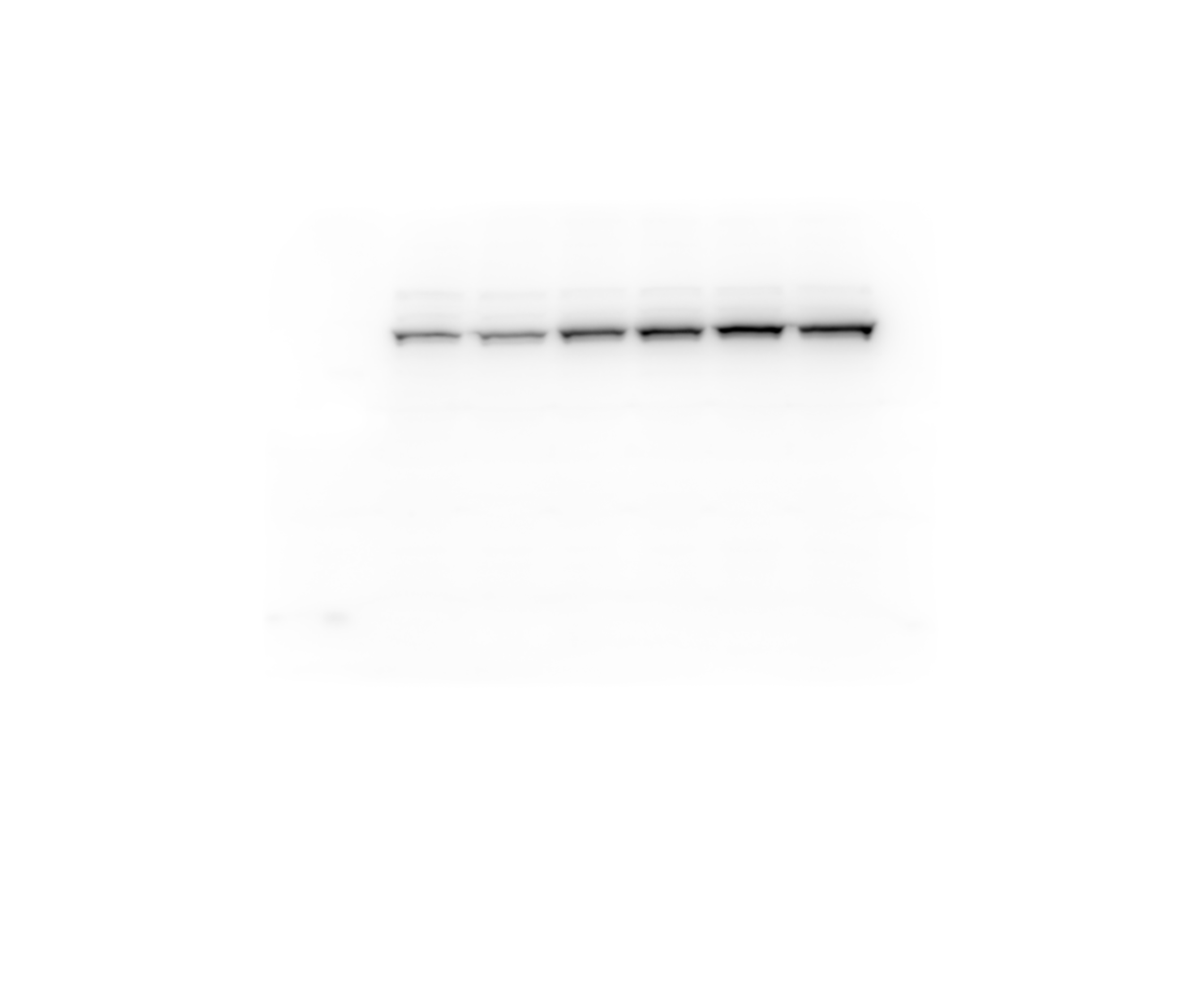

Supplement: Source data 2. [file elife-77755-data2.zip › Figure2-figure supplement1/Fig2-S1B Rai14.tif]

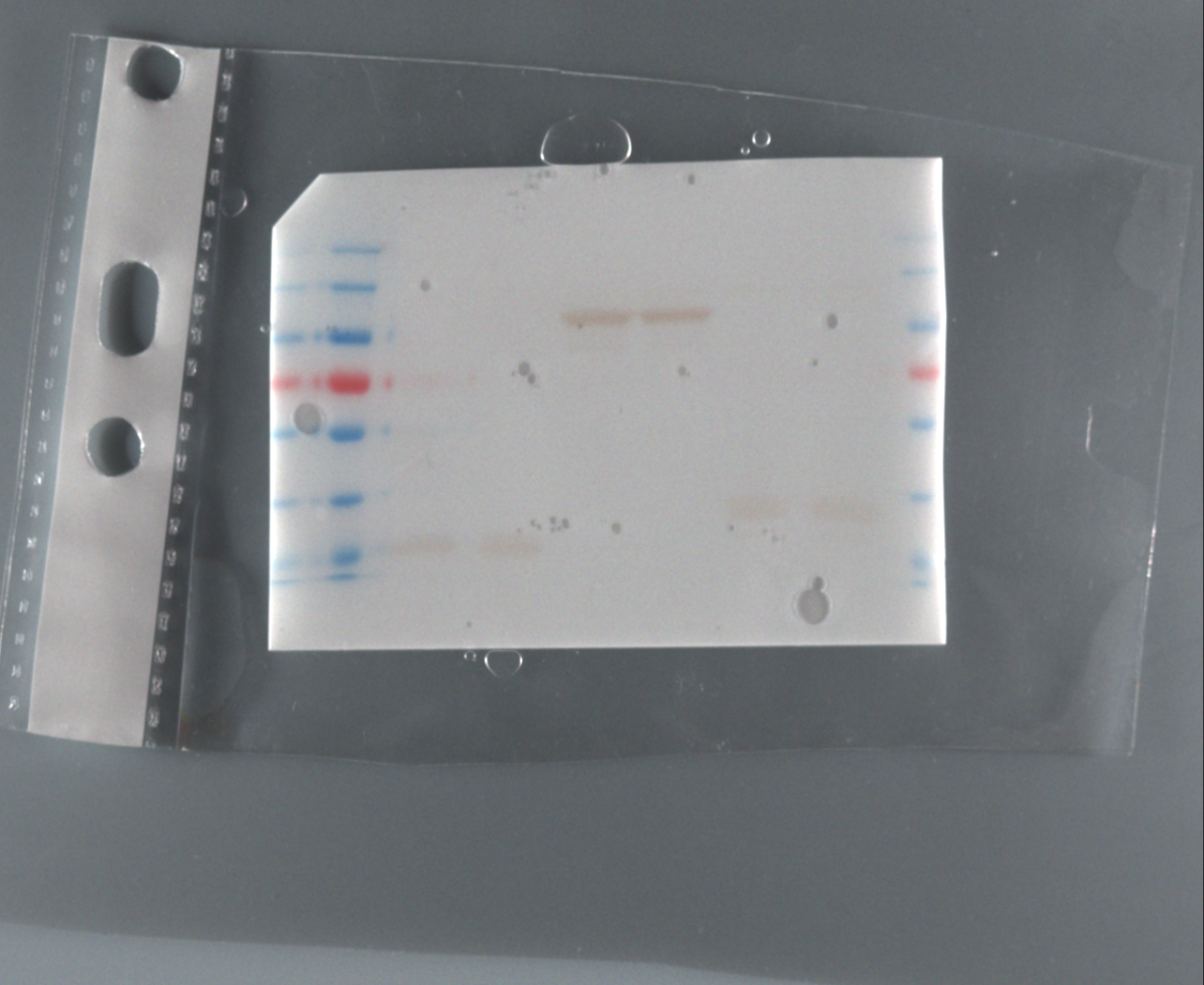

Supplement: Source data 2. [file elife-77755-data2.zip › Figure2-figure supplement1/Fig2-S1B-size marker for alpha-tubulin.tif]

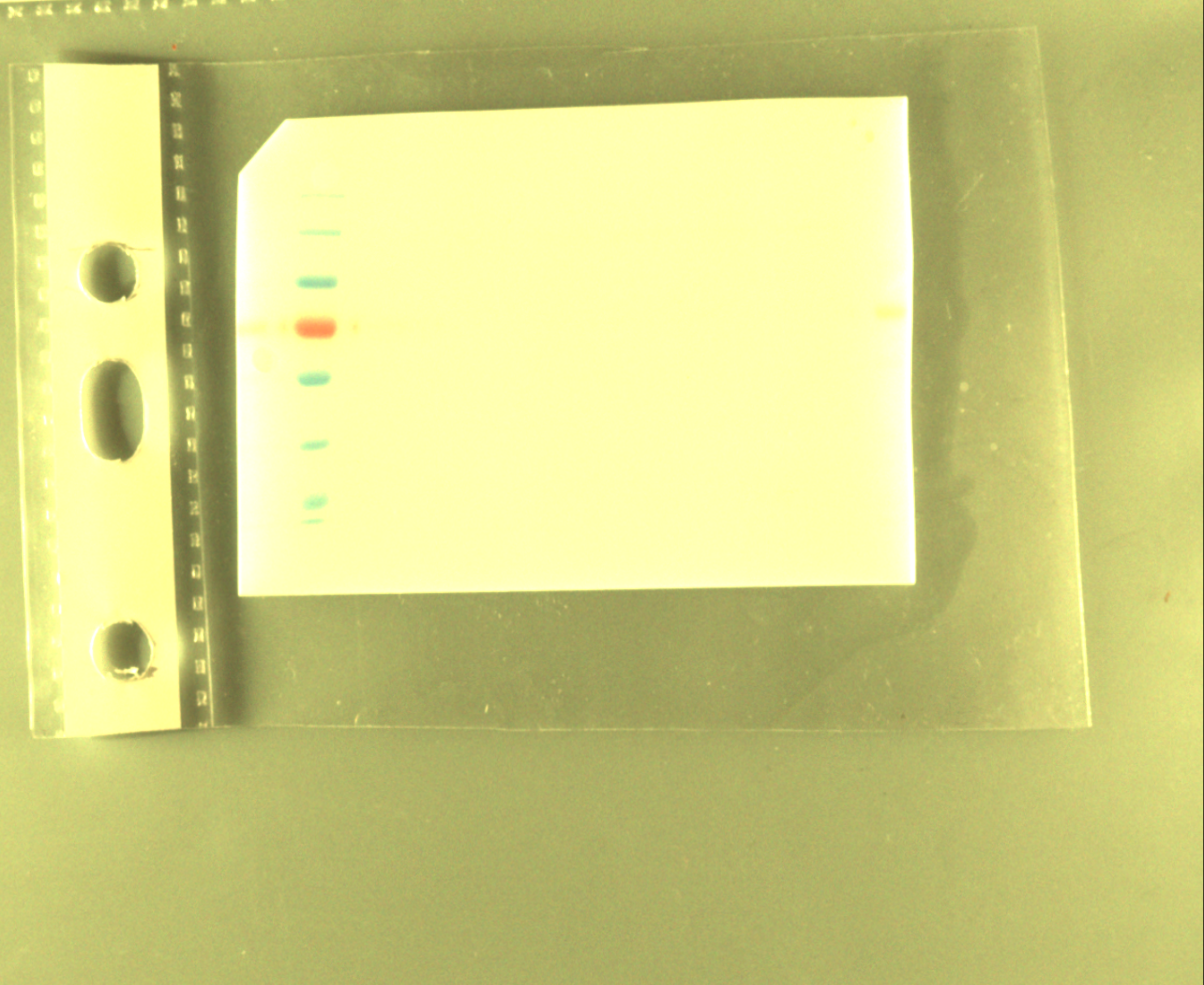

Supplement: Source data 2. [file elife-77755-data2.zip › Figure2-figure supplement1/Fig2-S1B-size marker for GFP (Tara).tif]

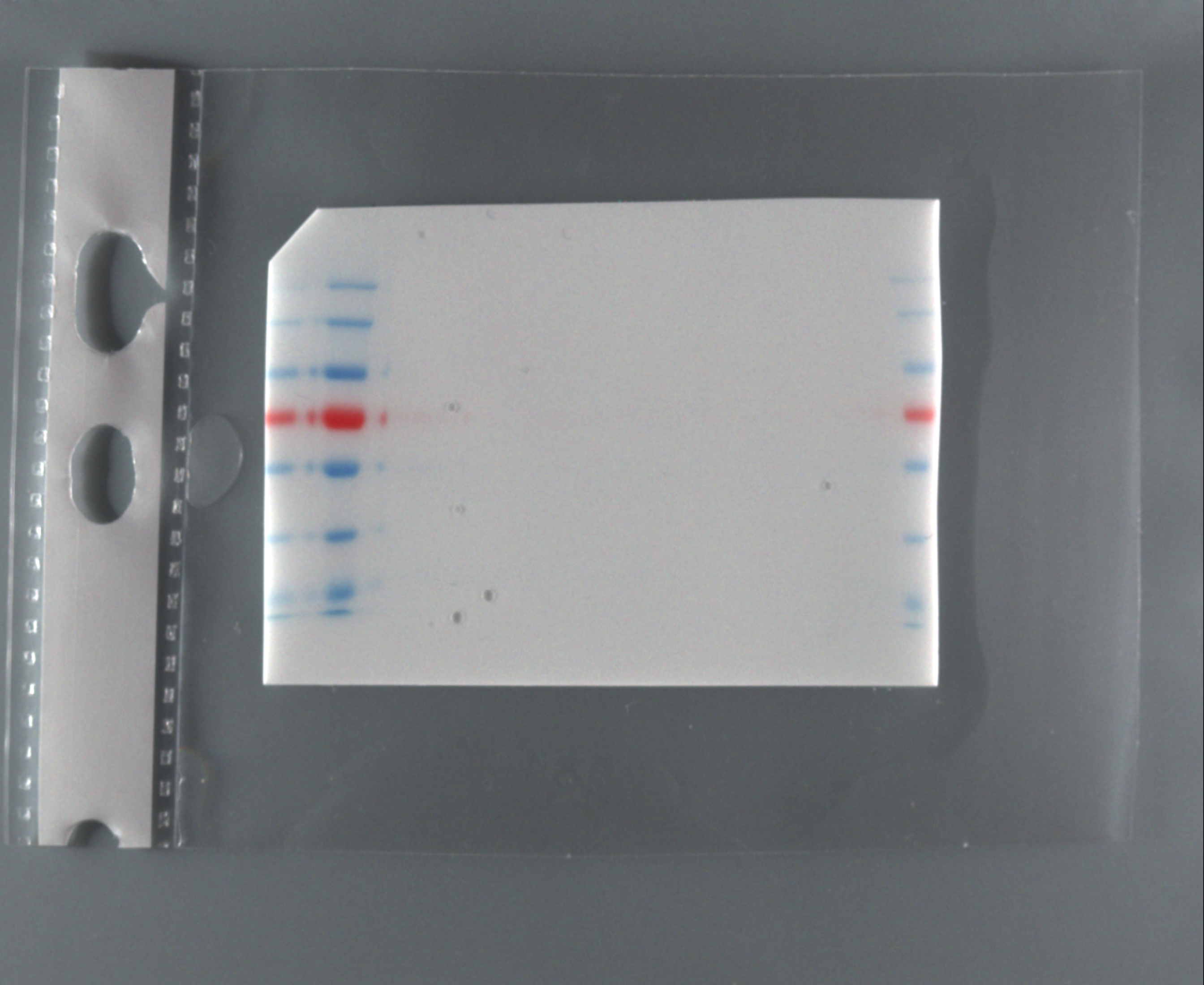

Supplement: Source data 2. [file elife-77755-data2.zip › Figure2-figure supplement1/Fig2-S1B-size marker for Rai14.tif]

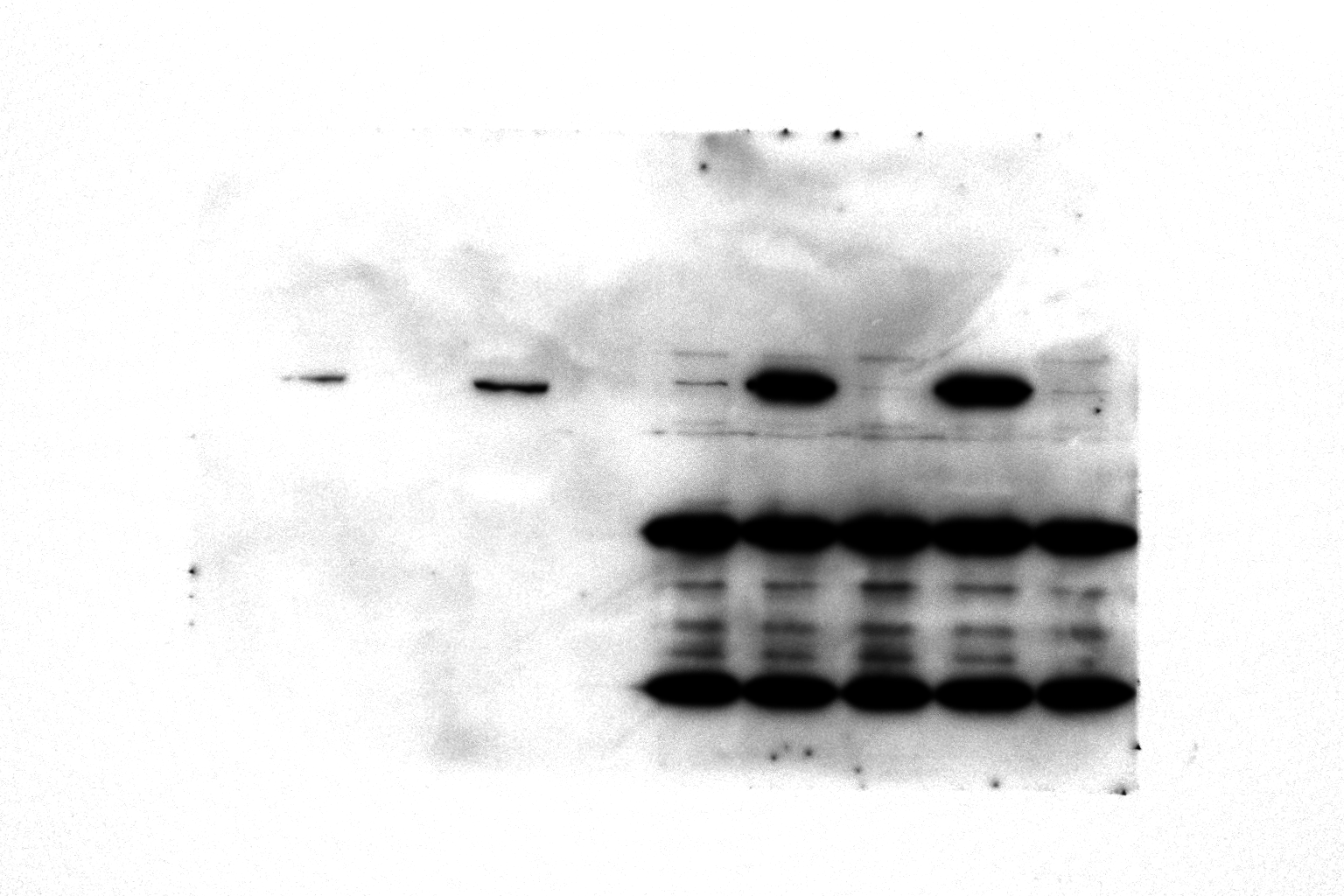

Supplement: Source data 2. [file elife-77755-data2.zip › Figure2-figure supplement2/Fig2-S2A FLAG (Rai14).tif]

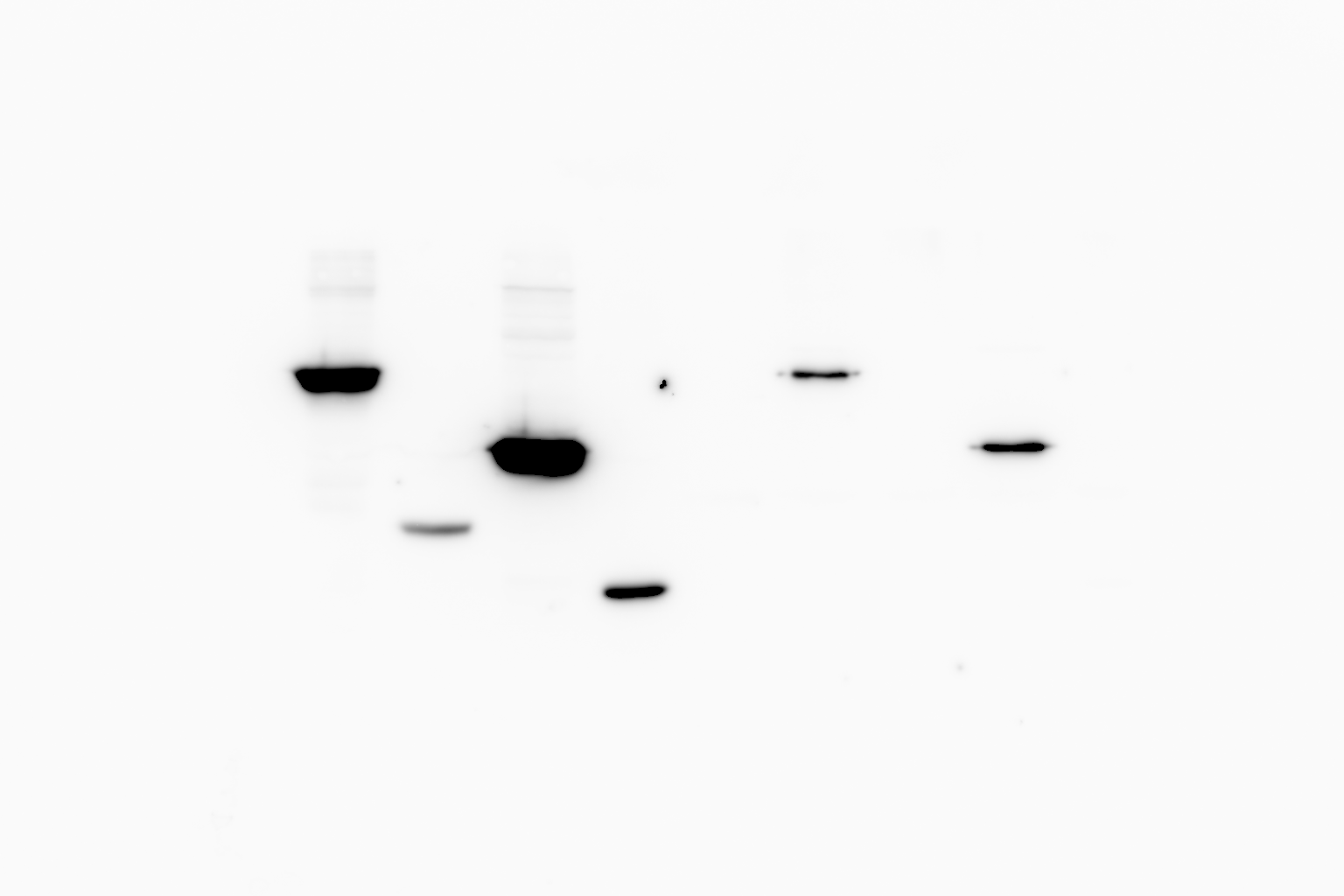

Supplement: Source data 2. [file elife-77755-data2.zip › Figure2-figure supplement2/Fig2-S2A GFP (Tara).tif]

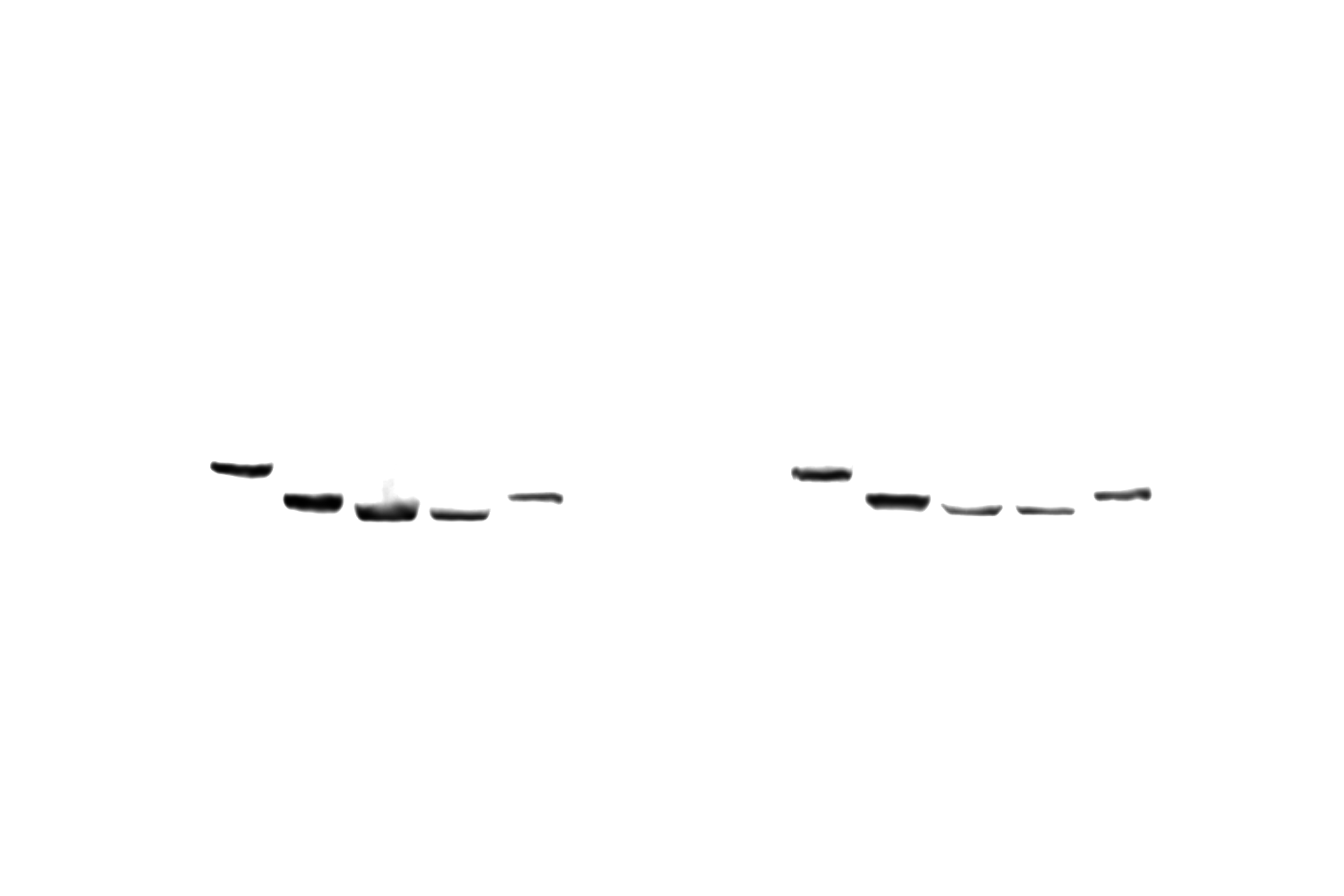

Supplement: Source data 2. [file elife-77755-data2.zip › Figure2-figure supplement2/Fig2-S2B GFP (Tara).tif]

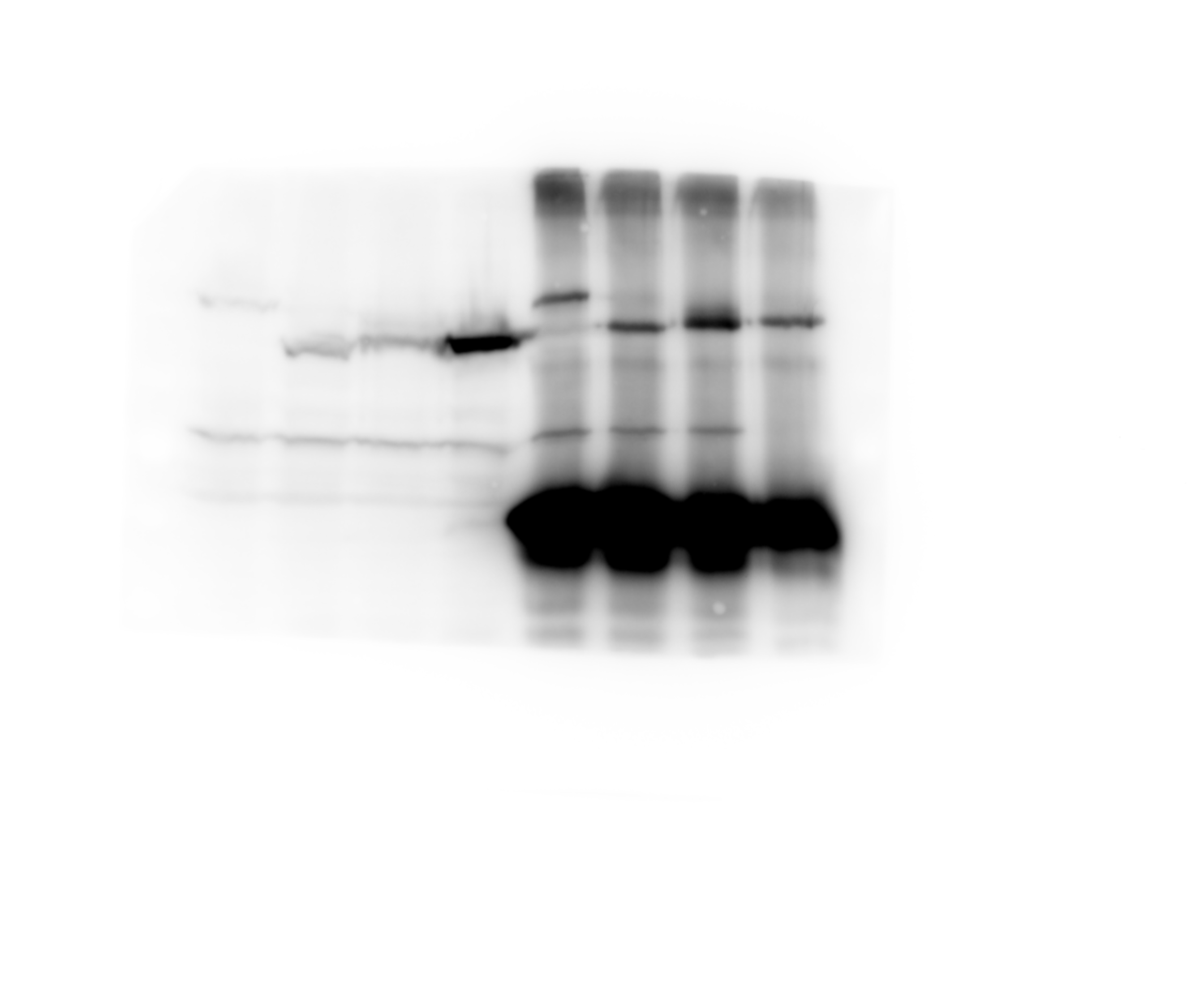

Supplement: Source data 3. [file elife-77755-data3.zip › Figure2-figure supplement3/Fig2-S3A GFP (Rai14).tif]

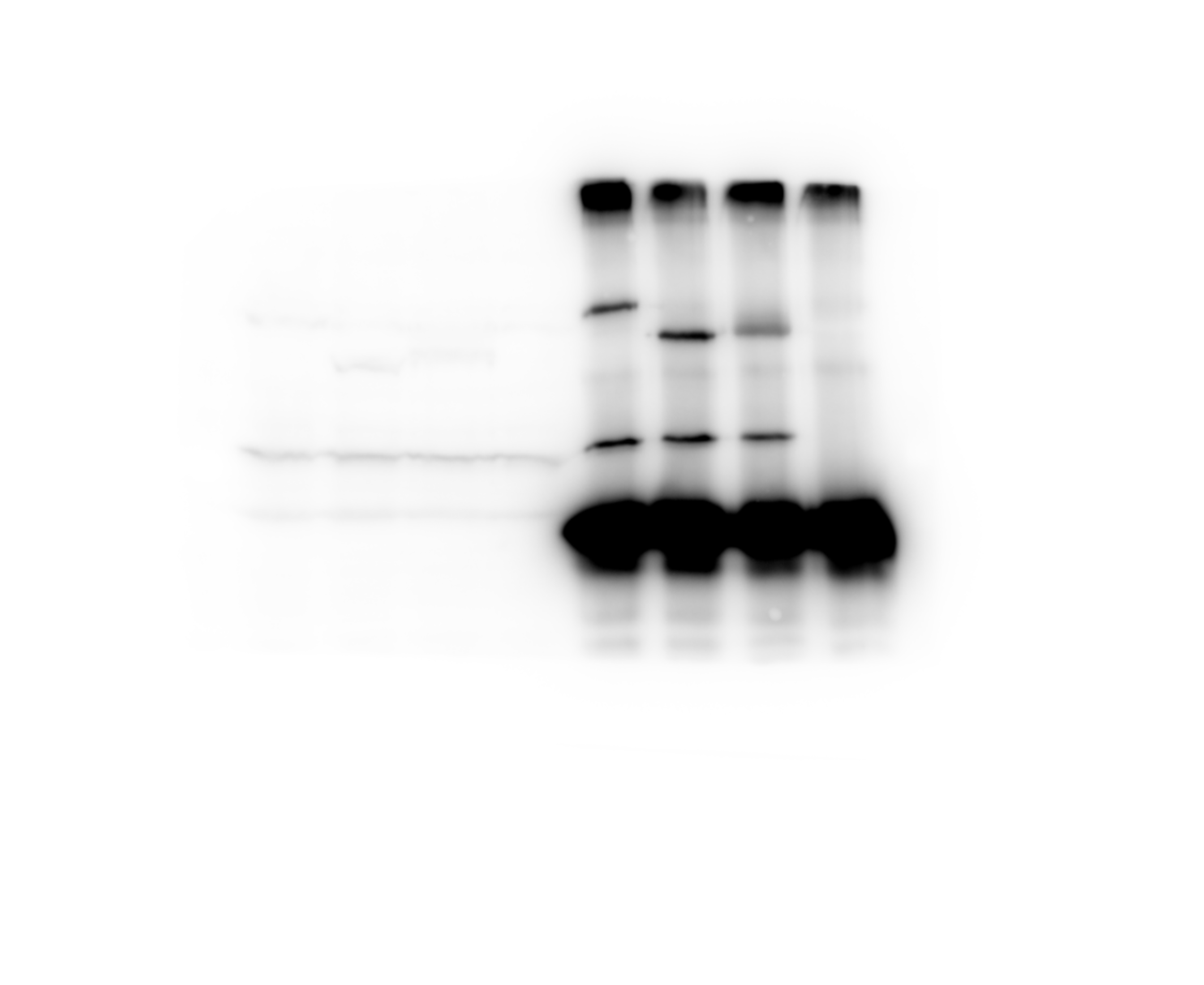

Supplement: Source data 3. [file elife-77755-data3.zip › Figure2-figure supplement3/Fig2-S3A Tara.tif]

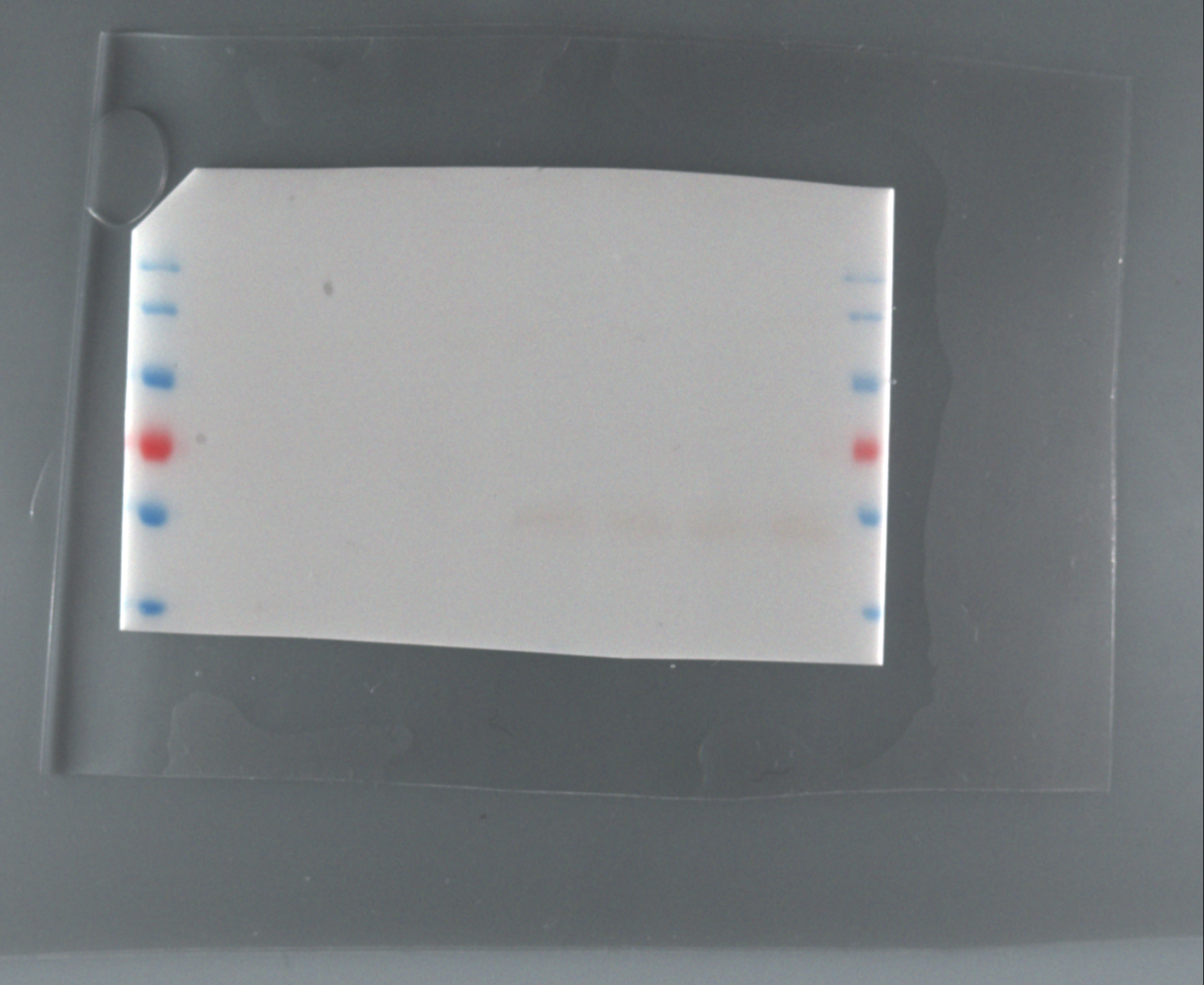

Supplement: Source data 3. [file elife-77755-data3.zip › Figure2-figure supplement3/Fig2-S3A-size marker for GFP (Rai14).tif]

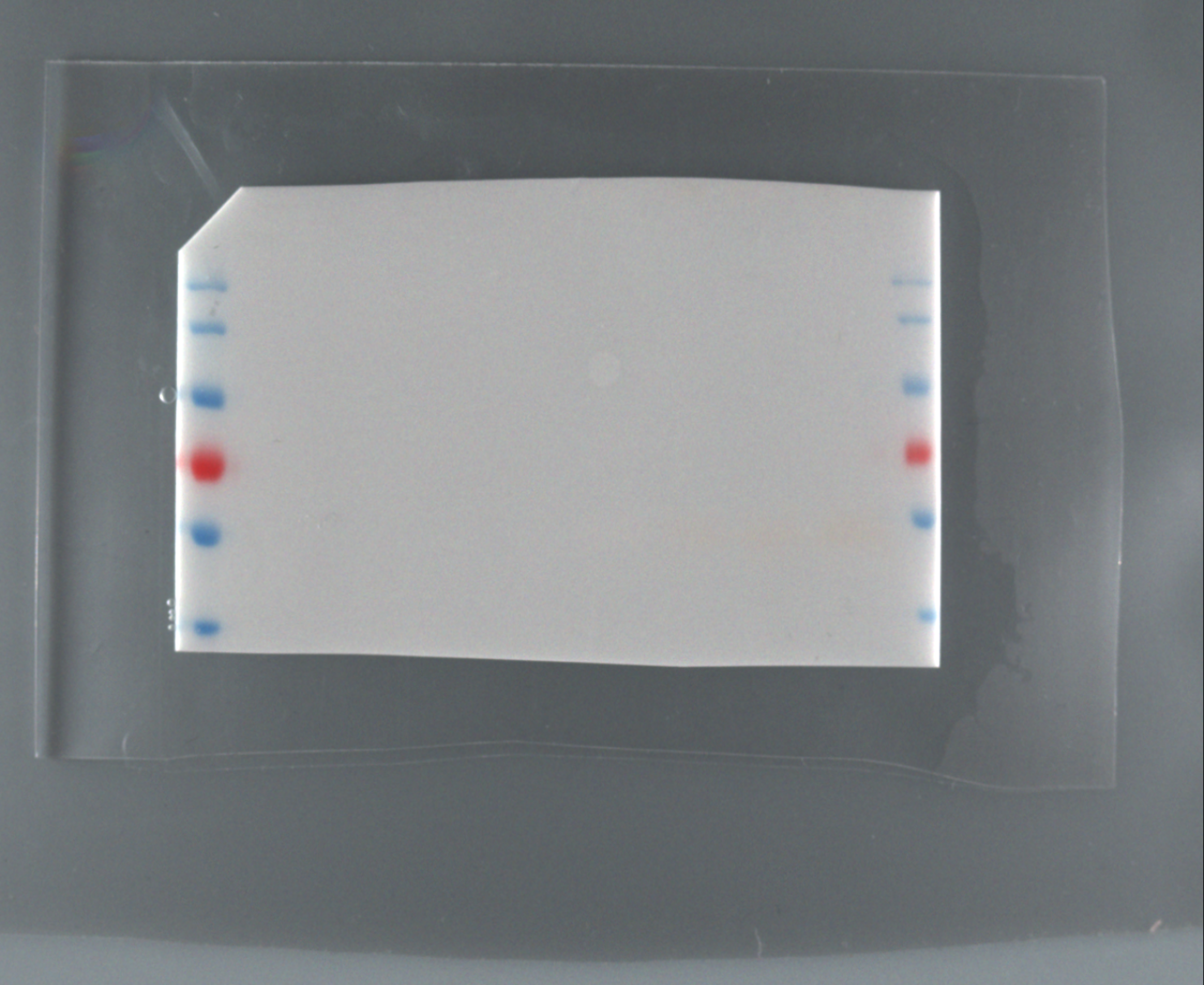

Supplement: Source data 3. [file elife-77755-data3.zip › Figure2-figure supplement3/Fig2-S3A-size marker for Tara.tif]

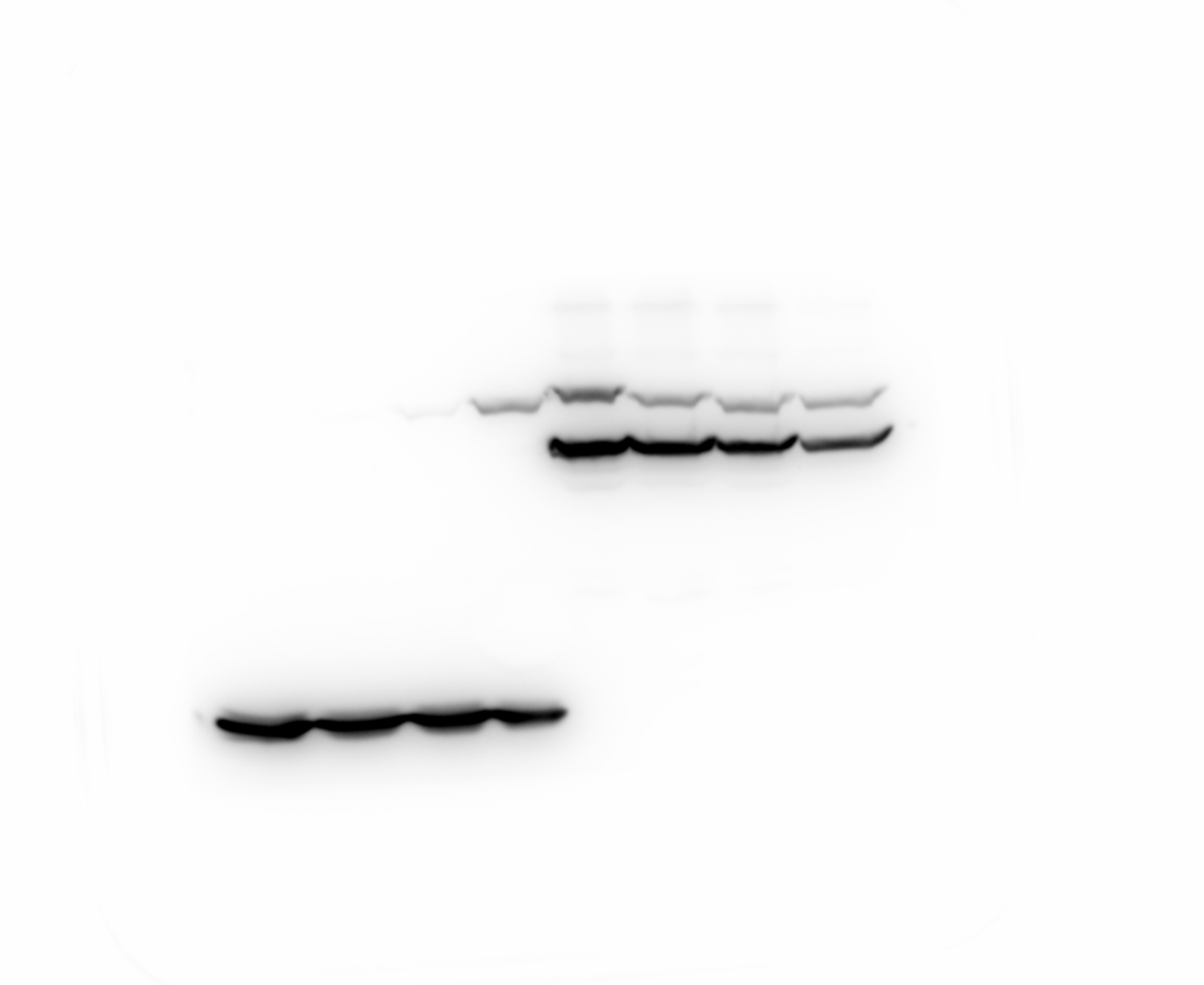

Supplement: Source data 3. [file elife-77755-data3.zip › Figure2-figure supplement3/Fig2-S3B GFP (Rai14).tif]

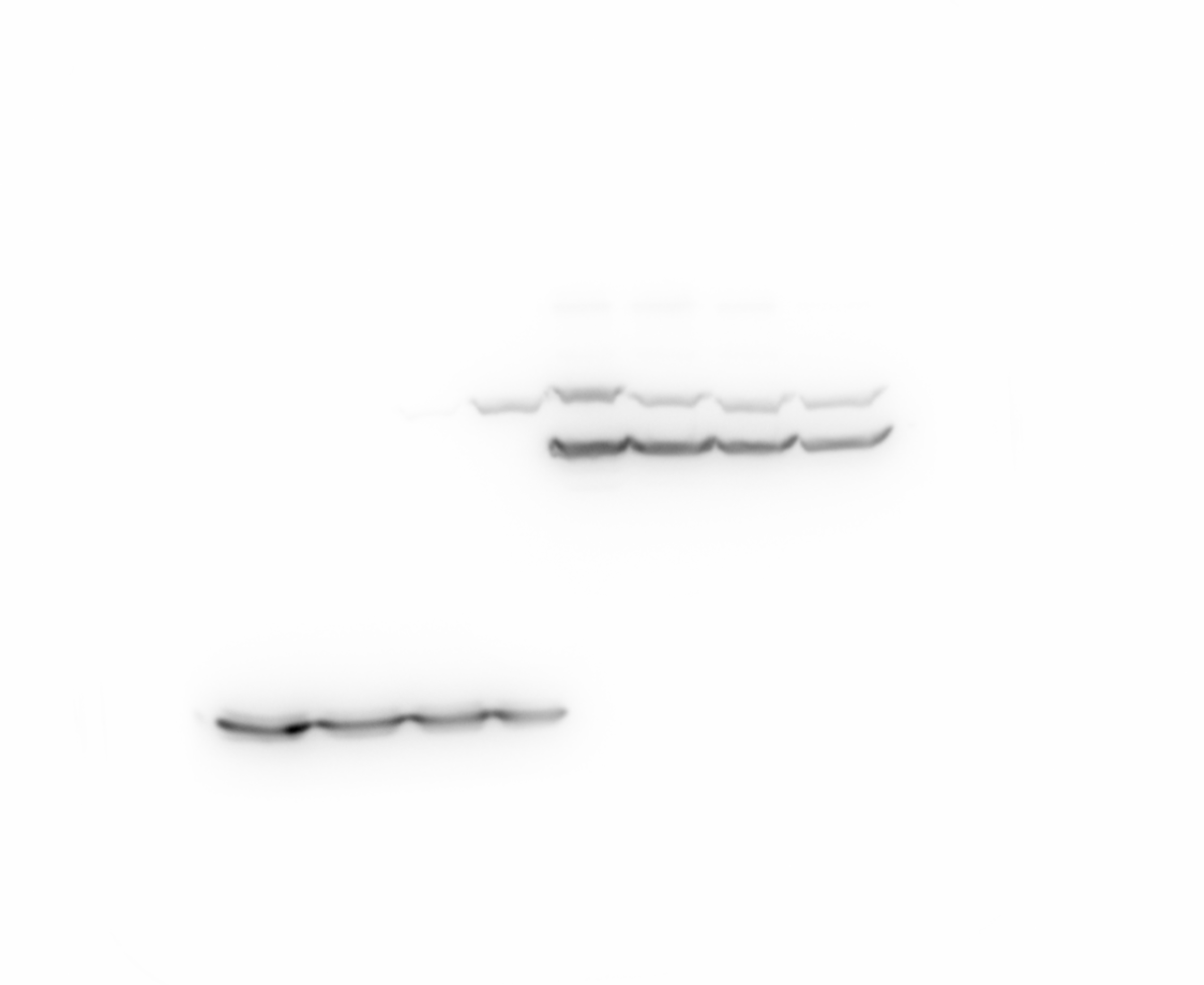

Supplement: Source data 3. [file elife-77755-data3.zip › Figure2-figure supplement3/Fig2-S3B GFP (Tara, GFP-c3).tif]

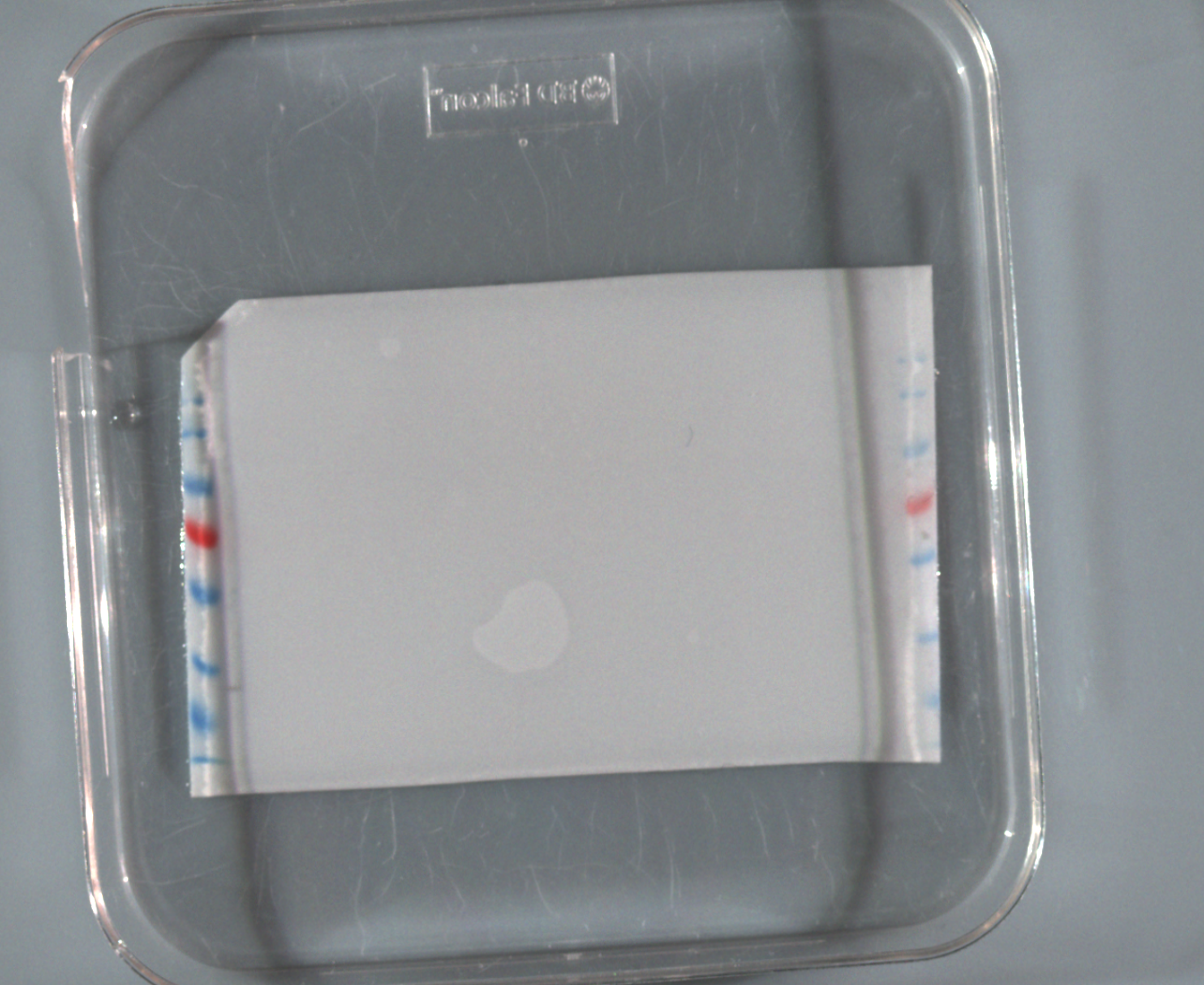

Supplement: Source data 3. [file elife-77755-data3.zip › Figure2-figure supplement3/Fig2-S3B-size marker for GFP (Rai14 and Tara).tif]

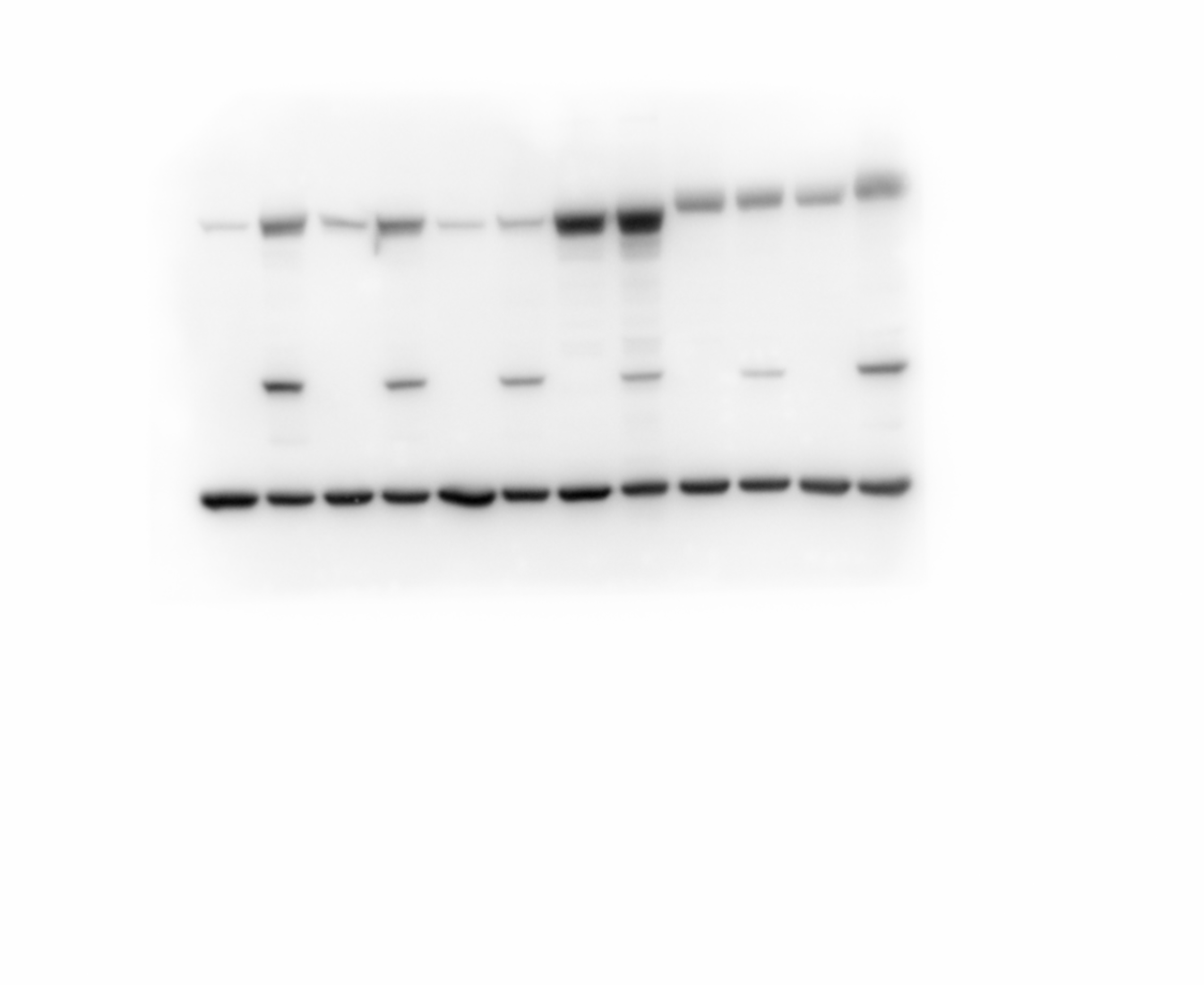

Supplement: Source data 3. [file elife-77755-data3.zip › Figure2-figure supplement3/Fig2-S3C alpha-tubulin.tif]

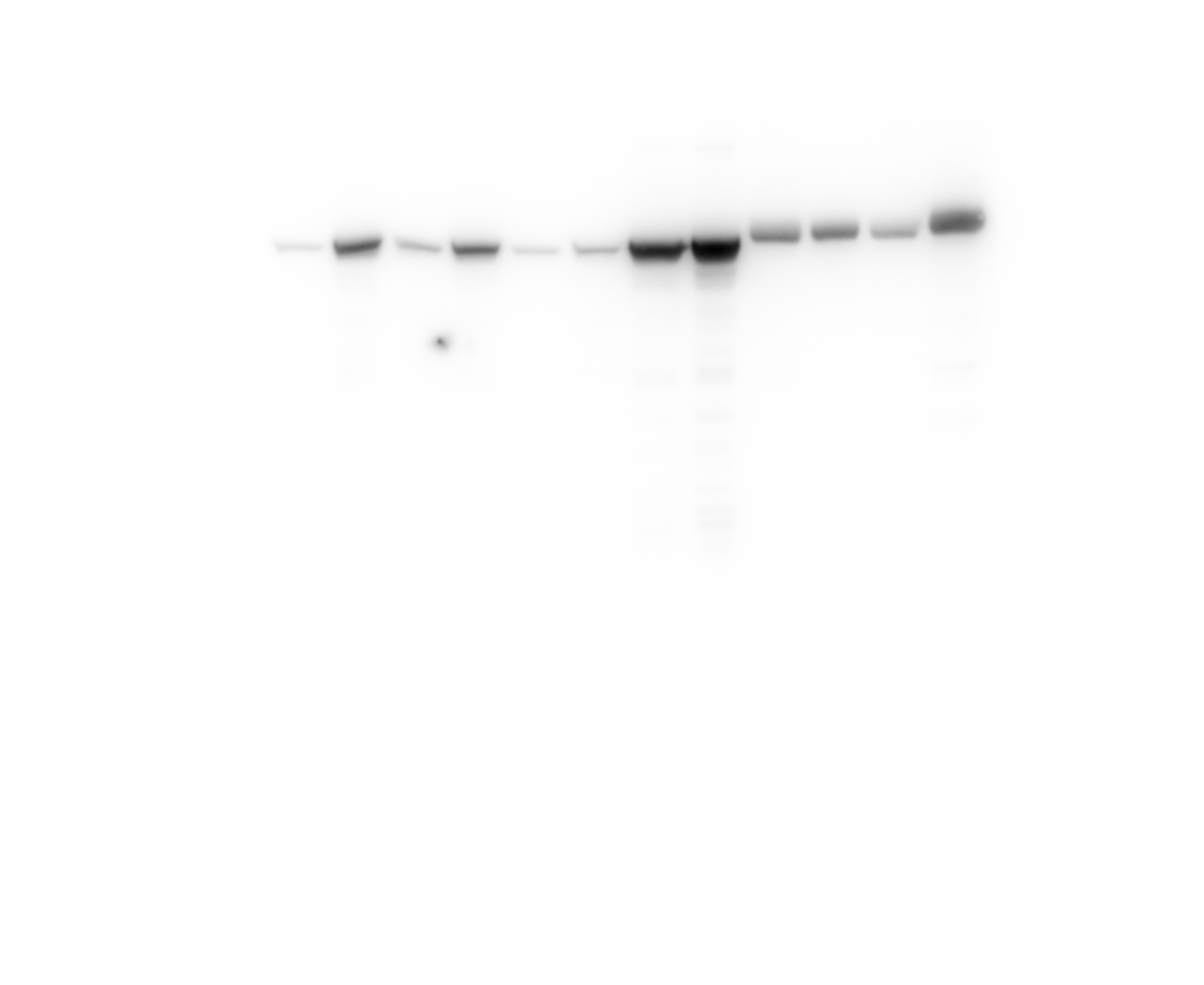

Supplement: Source data 3. [file elife-77755-data3.zip › Figure2-figure supplement3/Fig2-S3C GFP (Rai14).tif]

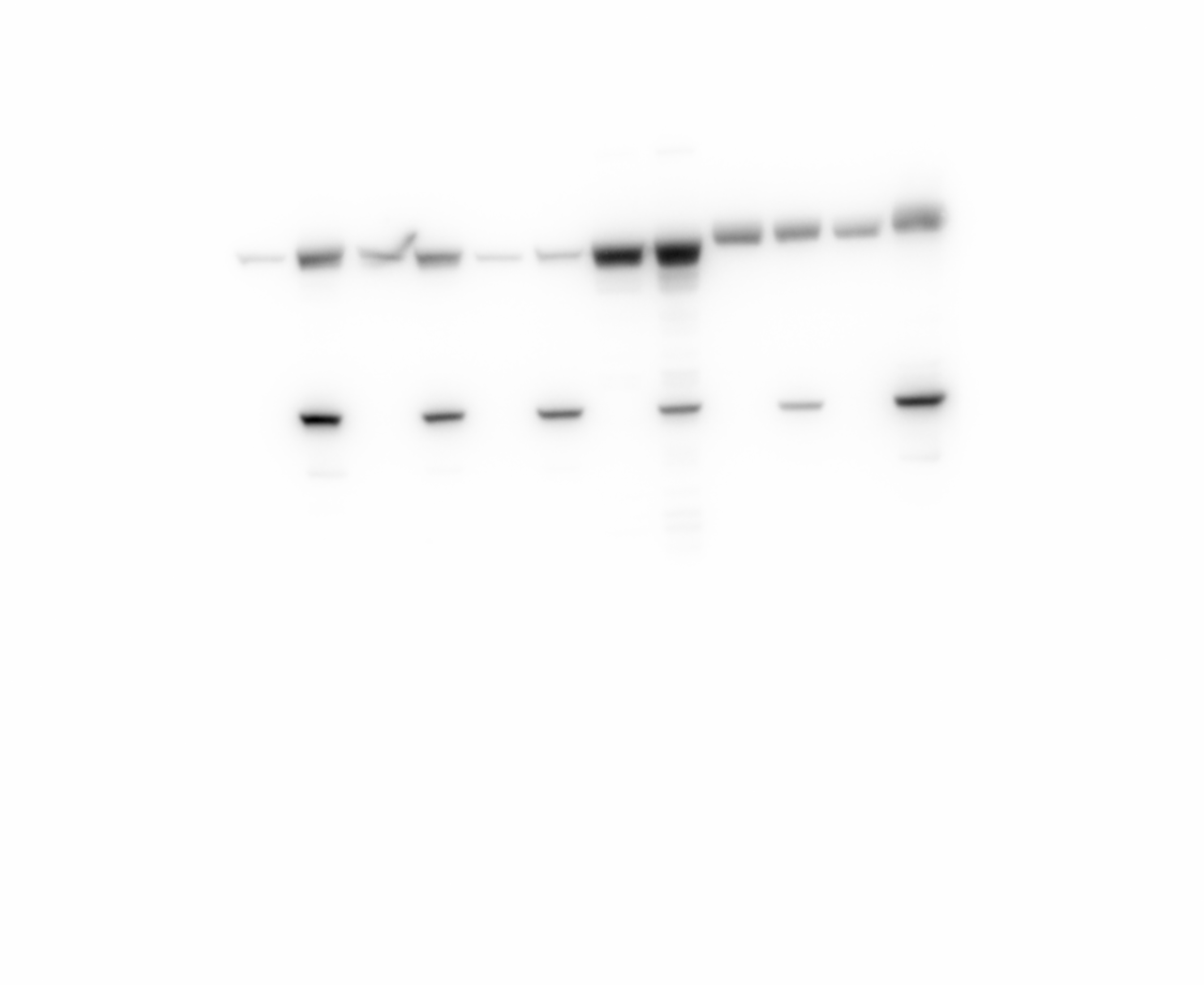

Supplement: Source data 3. [file elife-77755-data3.zip › Figure2-figure supplement3/Fig2-S3C Myc (Tara).tif]

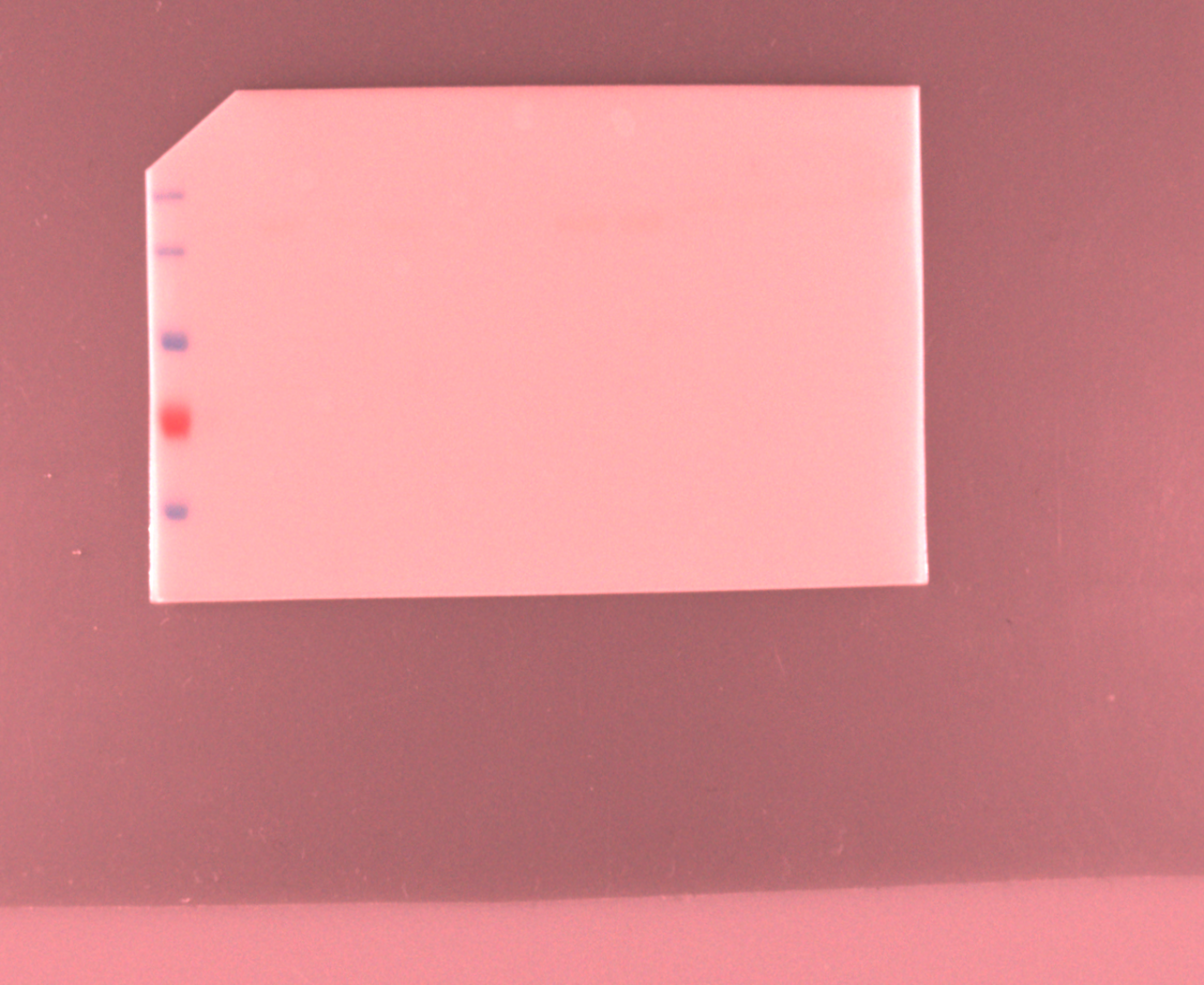

Supplement: Source data 3. [file elife-77755-data3.zip › Figure2-figure supplement3/Fig2-S3C-size marker for alpha-tubulin.tif]

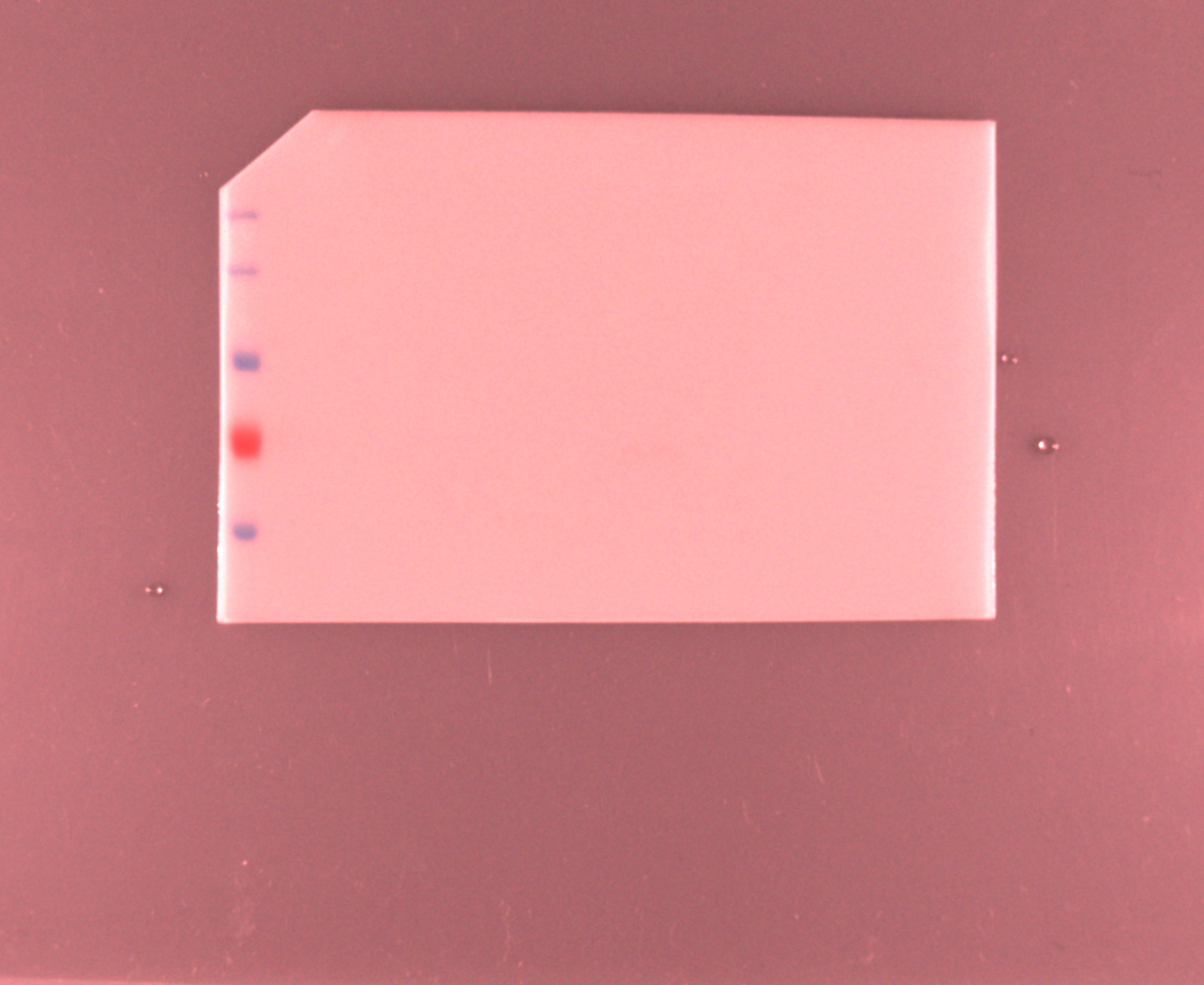

Supplement: Source data 3. [file elife-77755-data3.zip › Figure2-figure supplement3/Fig2-S3C-size marker for GFP (Rai14).tif]

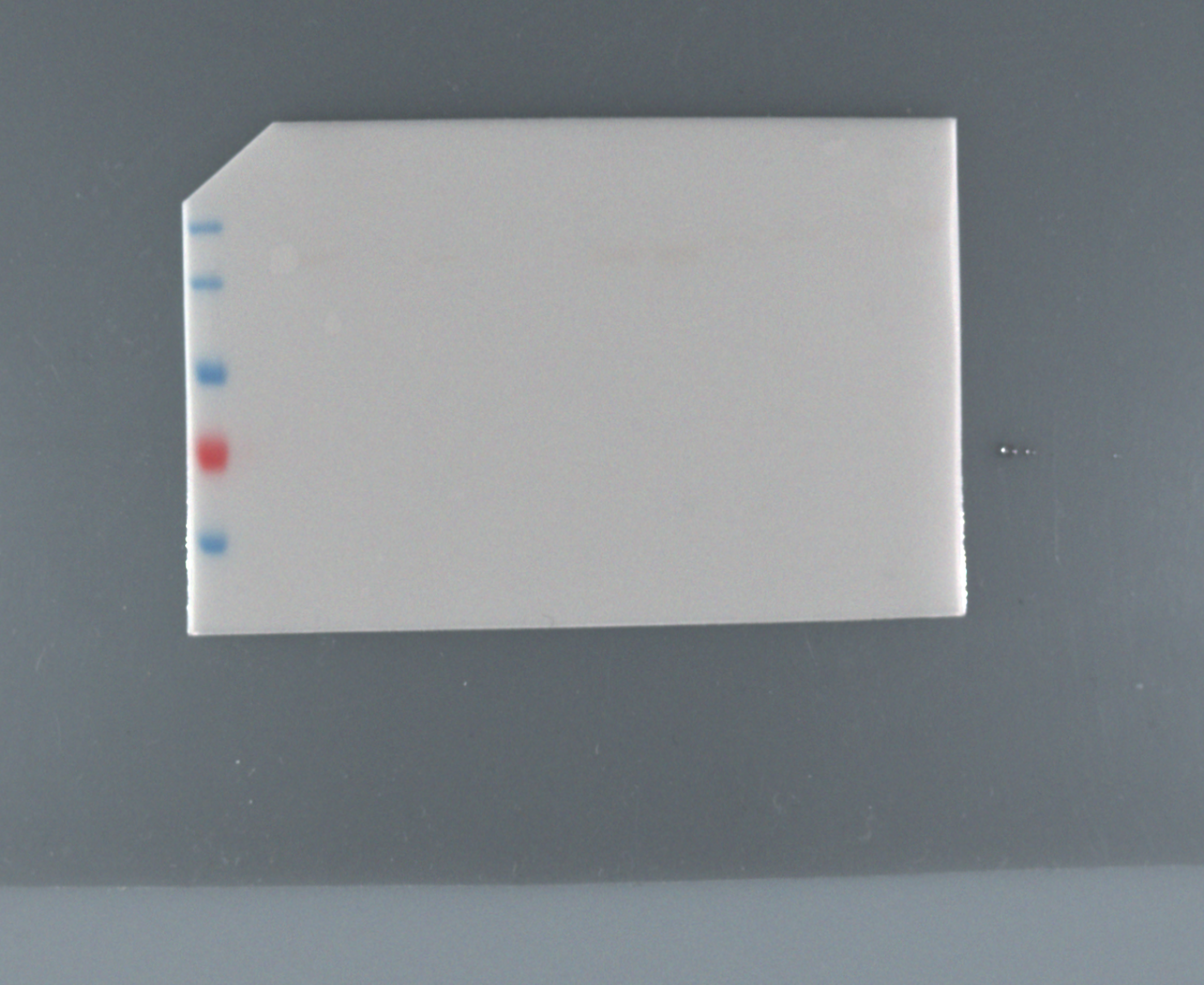

Supplement: Source data 3. [file elife-77755-data3.zip › Figure2-figure supplement3/Fig2-S3C-size marker for Myc (Tara).tif]

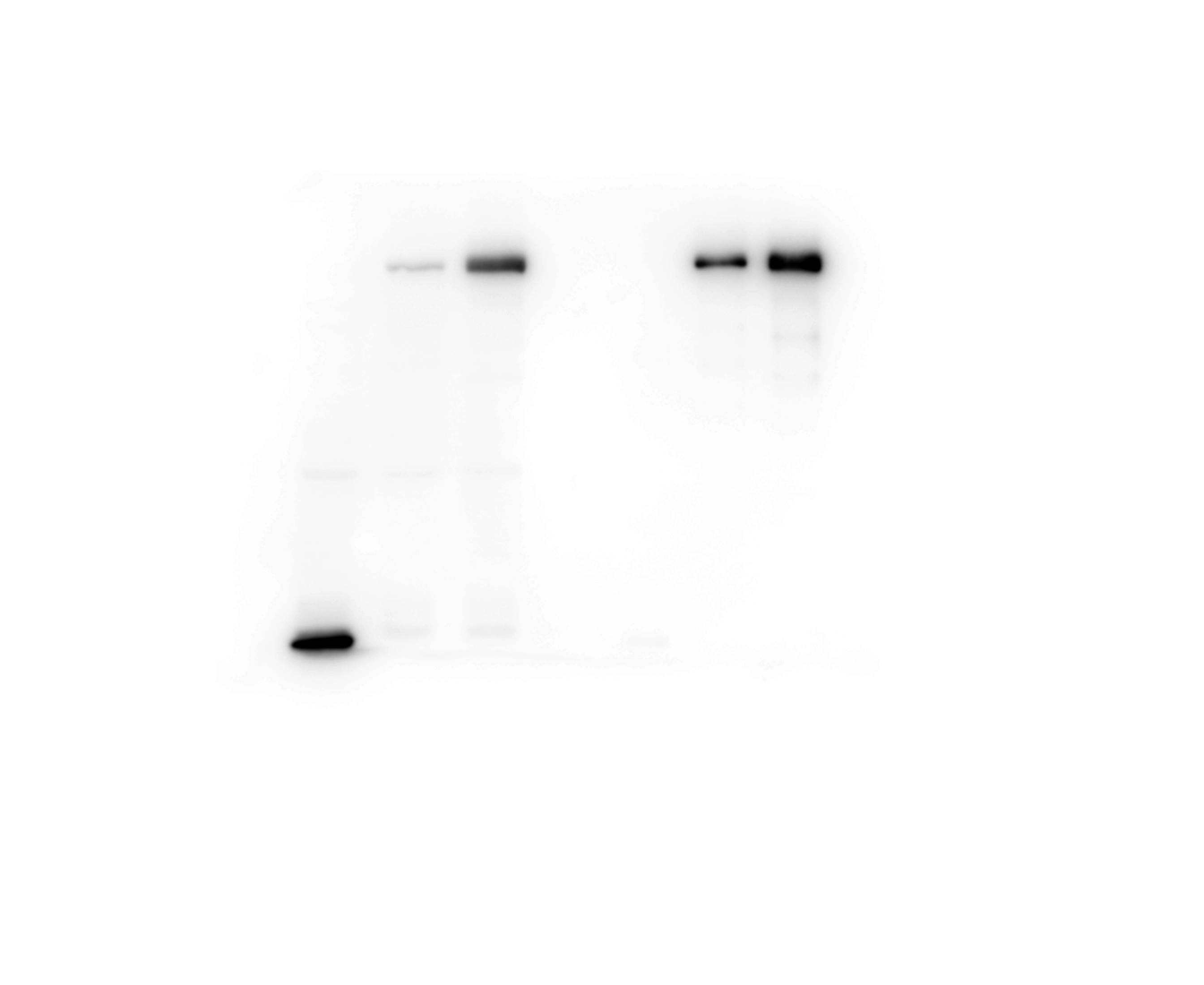

Supplement: Source data 3. [file elife-77755-data3.zip › Figure2-figure supplement3/Fig2-S3D GFP (Rai14).tif]

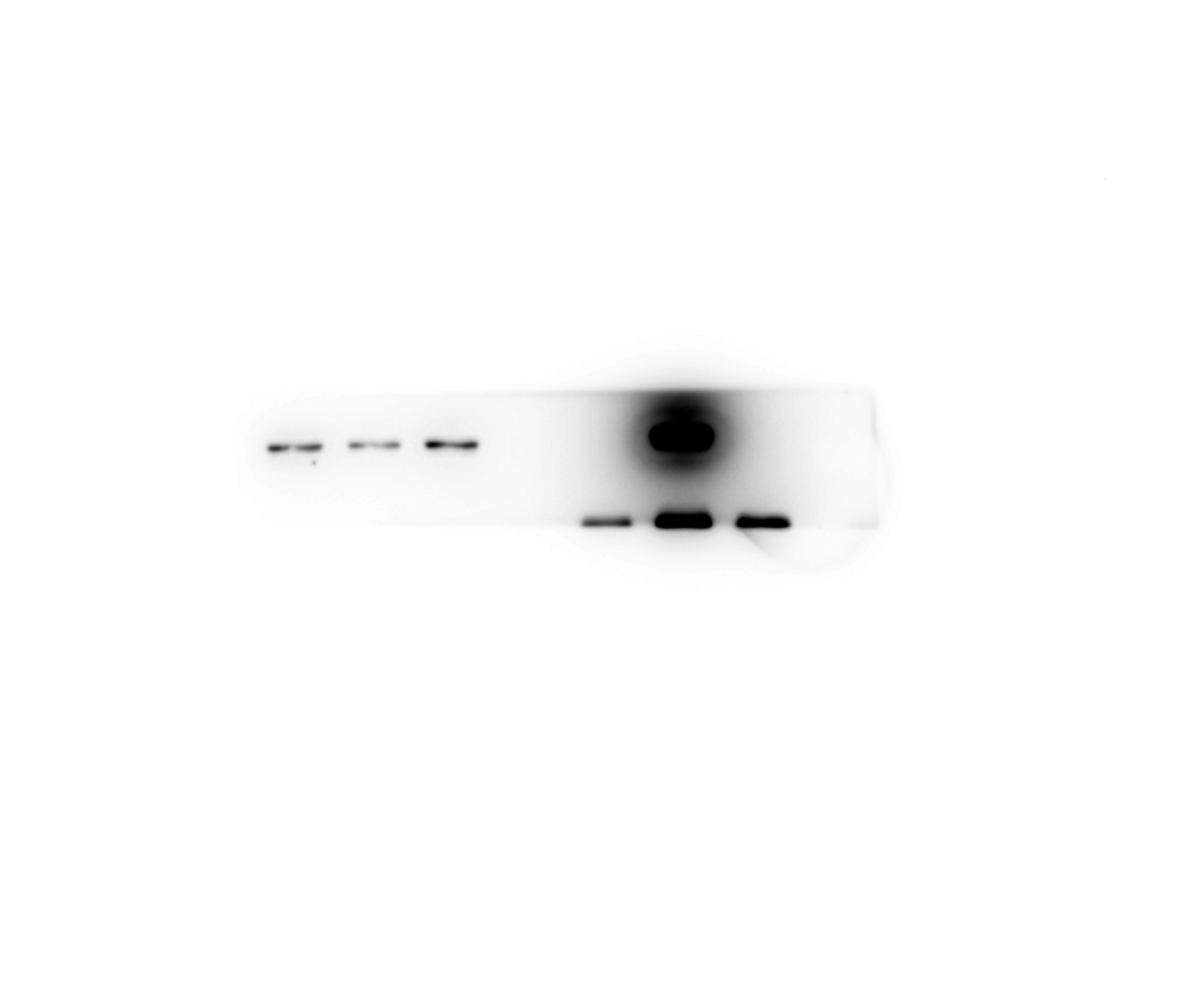

Supplement: Source data 3. [file elife-77755-data3.zip › Figure2-figure supplement3/Fig2-S3D Tara (input, long exposure).tif]

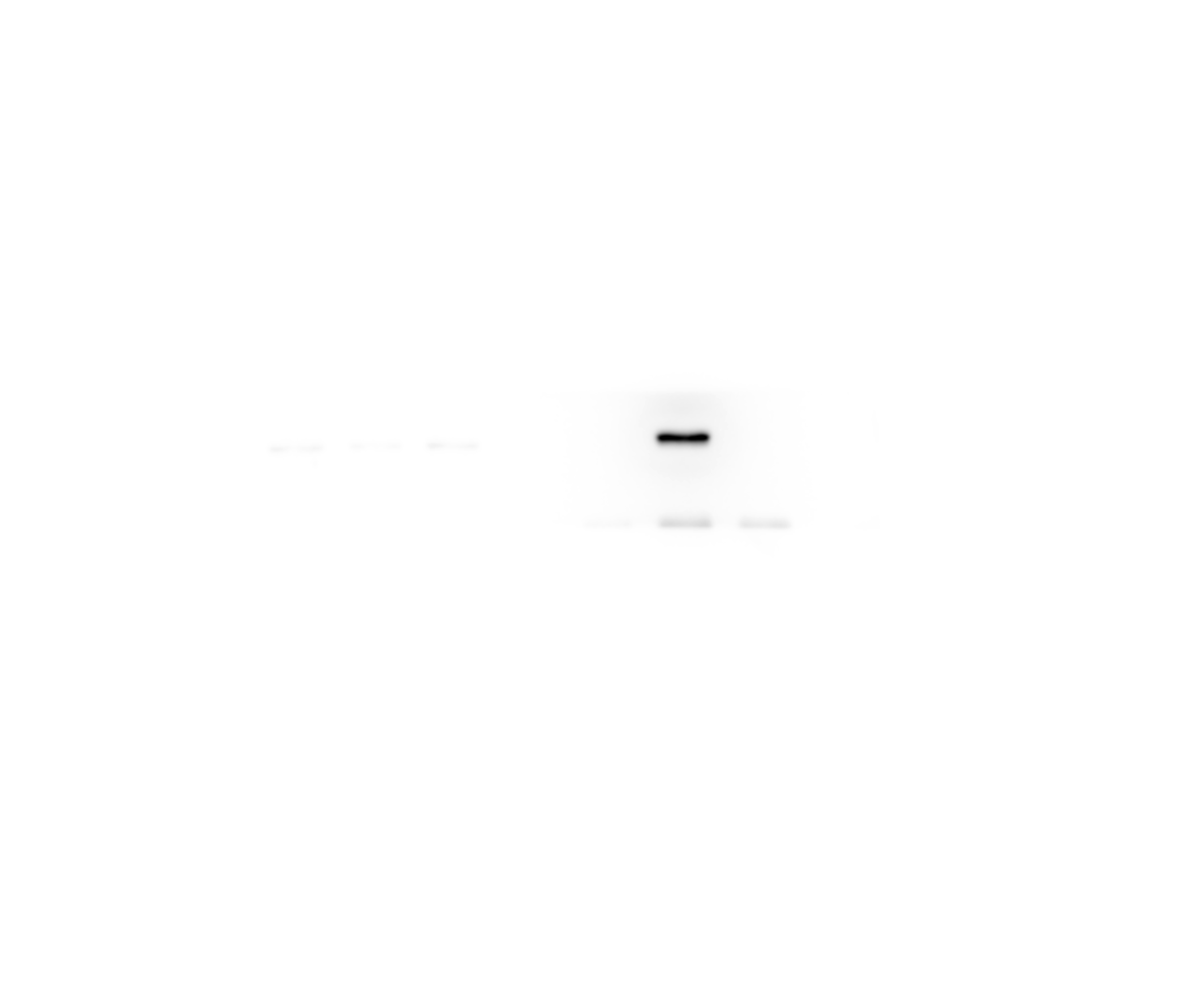

Supplement: Source data 3. [file elife-77755-data3.zip › Figure2-figure supplement3/Fig2-S3D Tara (IP, short exposure).tif]

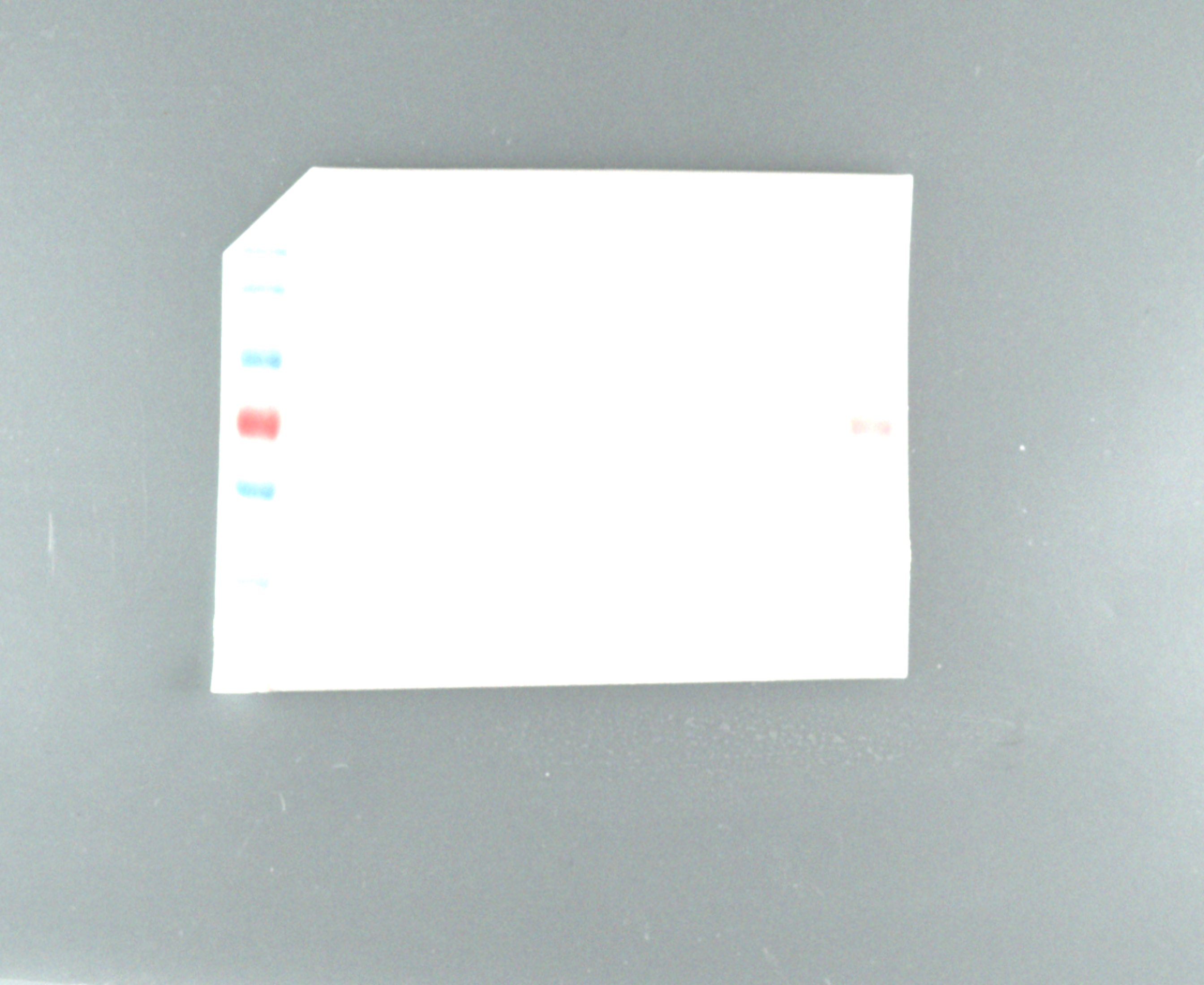

Supplement: Source data 3. [file elife-77755-data3.zip › Figure2-figure supplement3/Fig2-S3D-size marker for GFP (Rai14).tif]

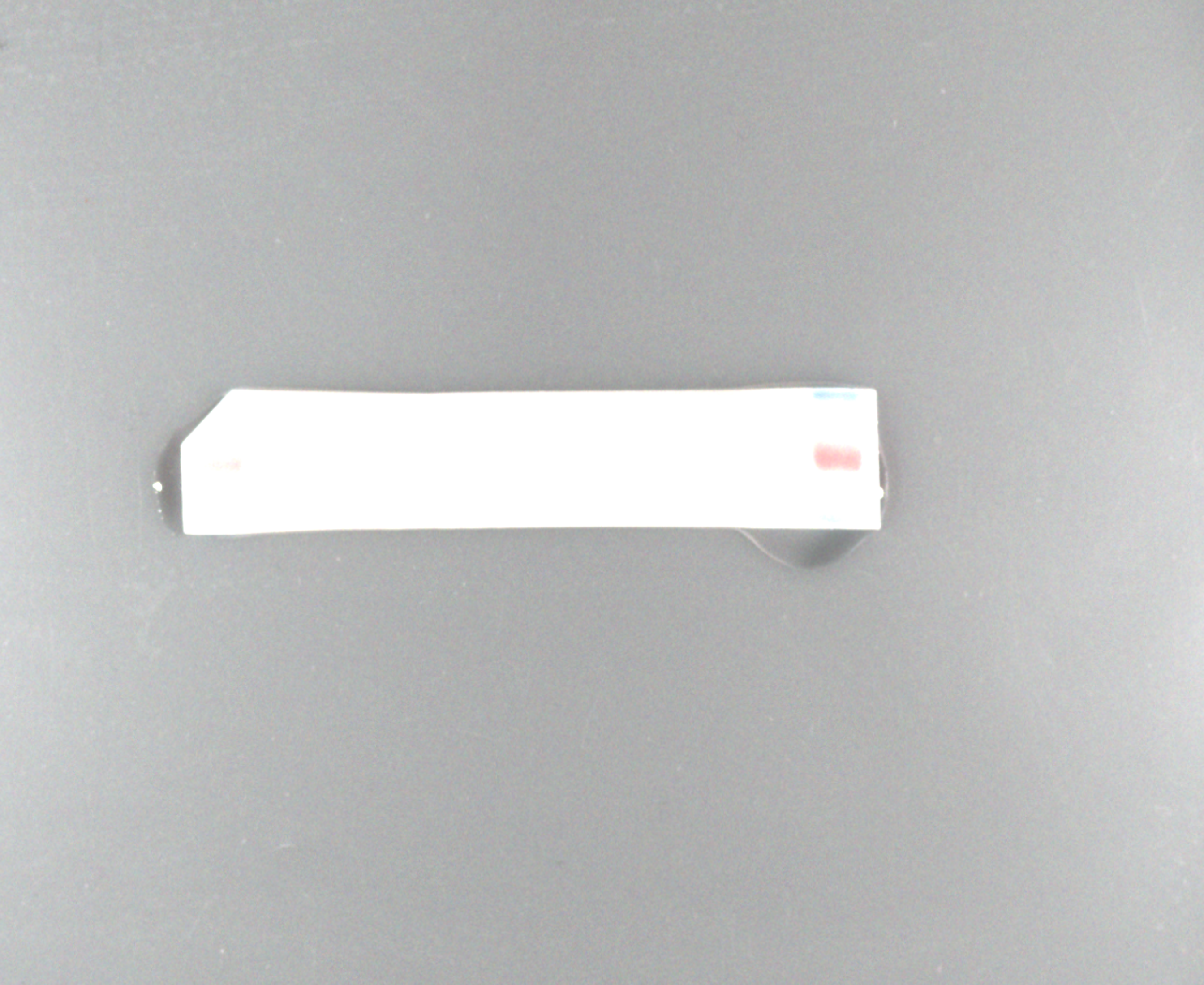

Supplement: Source data 3. [file elife-77755-data3.zip › Figure2-figure supplement3/Fig2-S3D-size marker for Tara.tif]

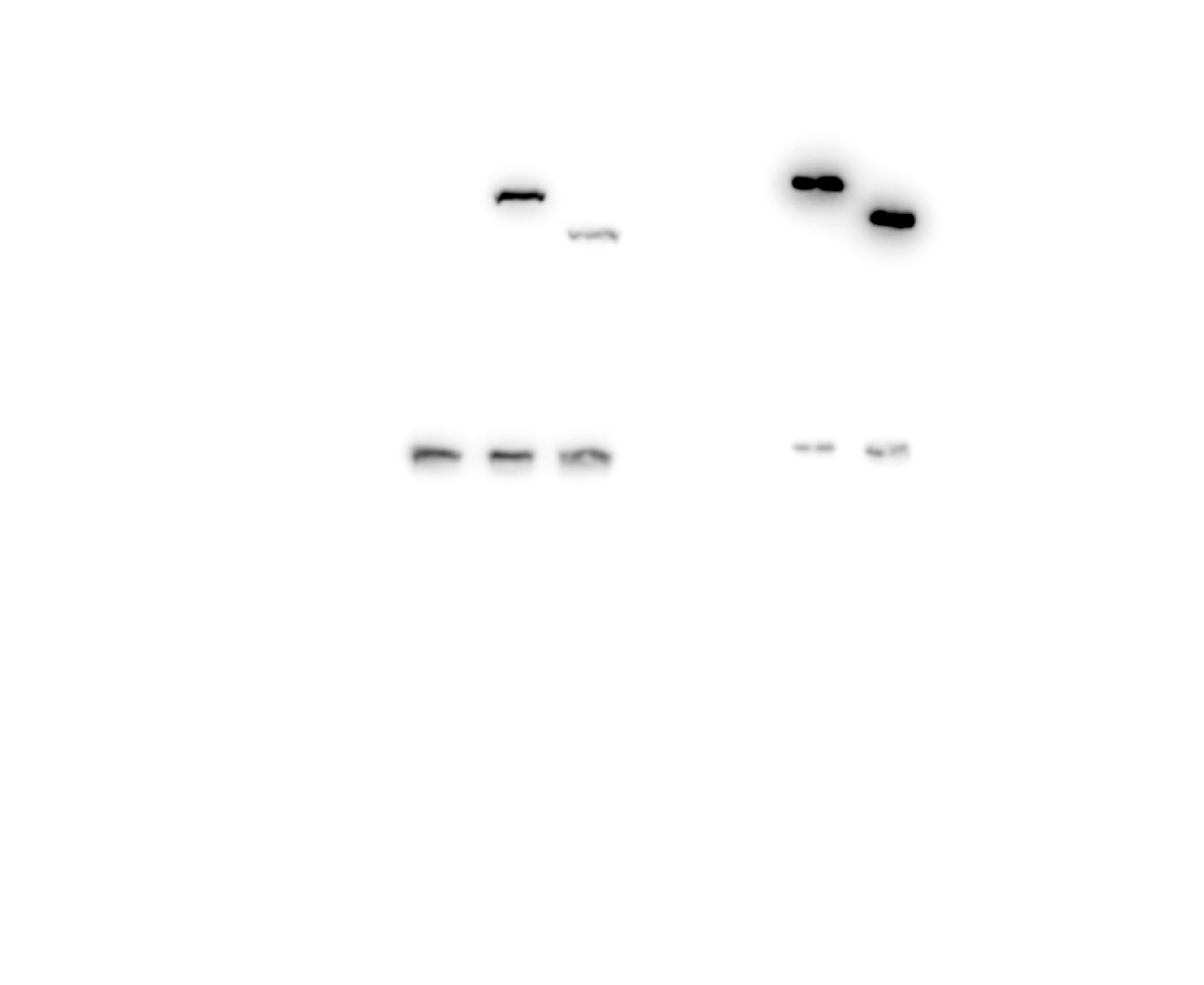

Supplement: Source data 3. [file elife-77755-data3.zip › Figure3-figure supplement1/Fig3-S1B GFP (Rai14).tif]

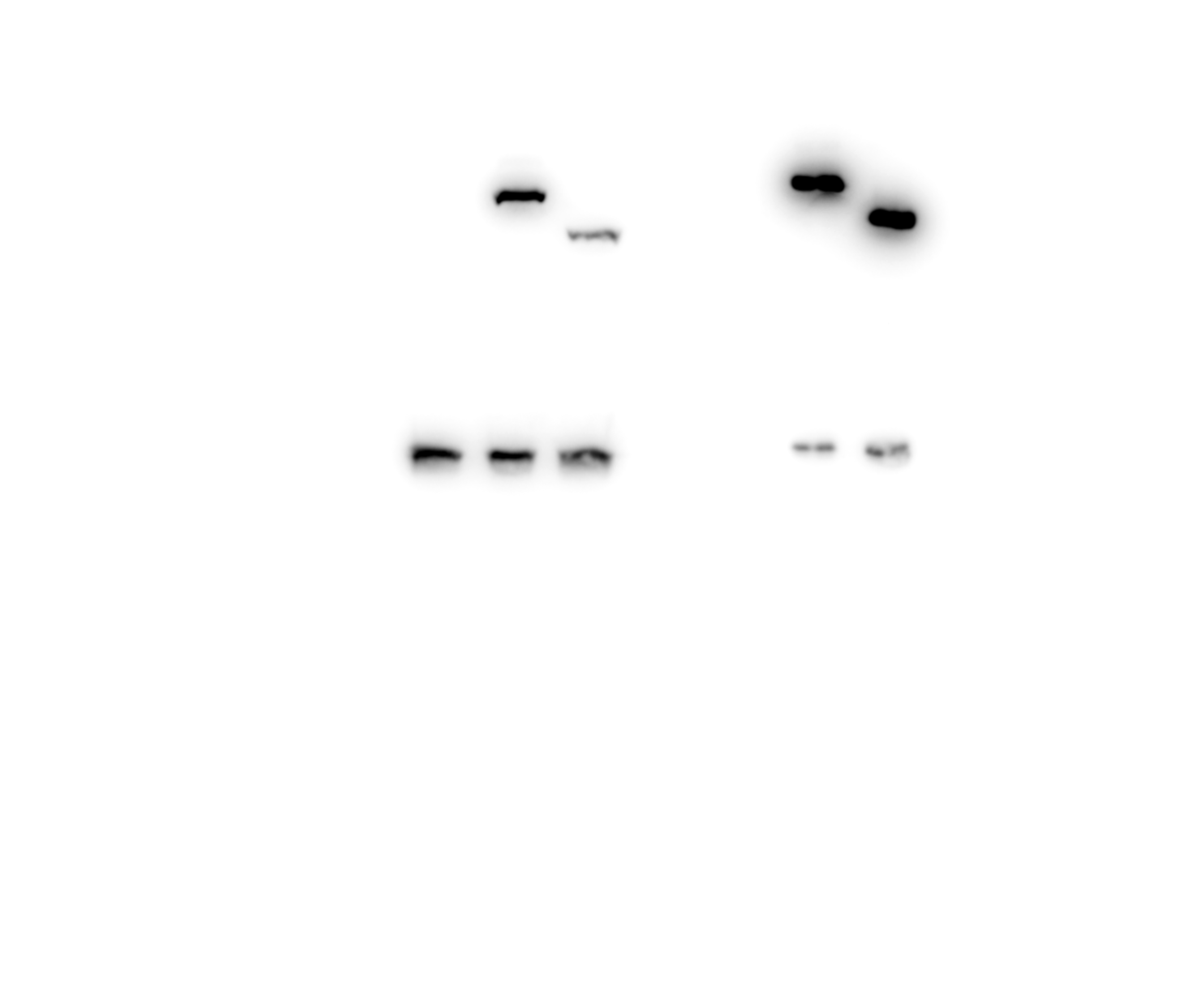

Supplement: Source data 3. [file elife-77755-data3.zip › Figure3-figure supplement1/Fig3-S1B Tara.tif]

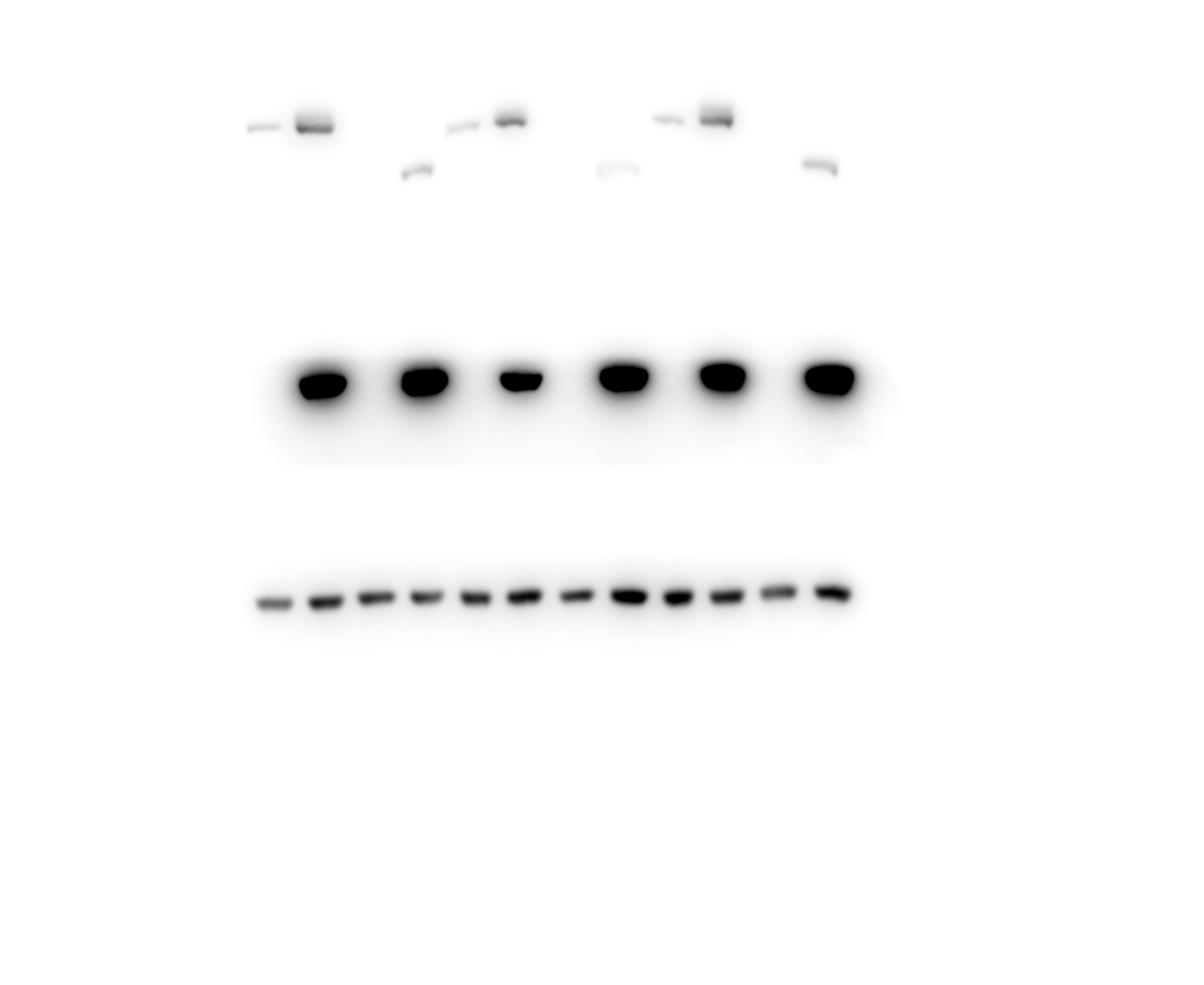

Supplement: Source data 3. [file elife-77755-data3.zip › Figure3-figure supplement1/Fig3-S1C alpha-tubulin.tif]

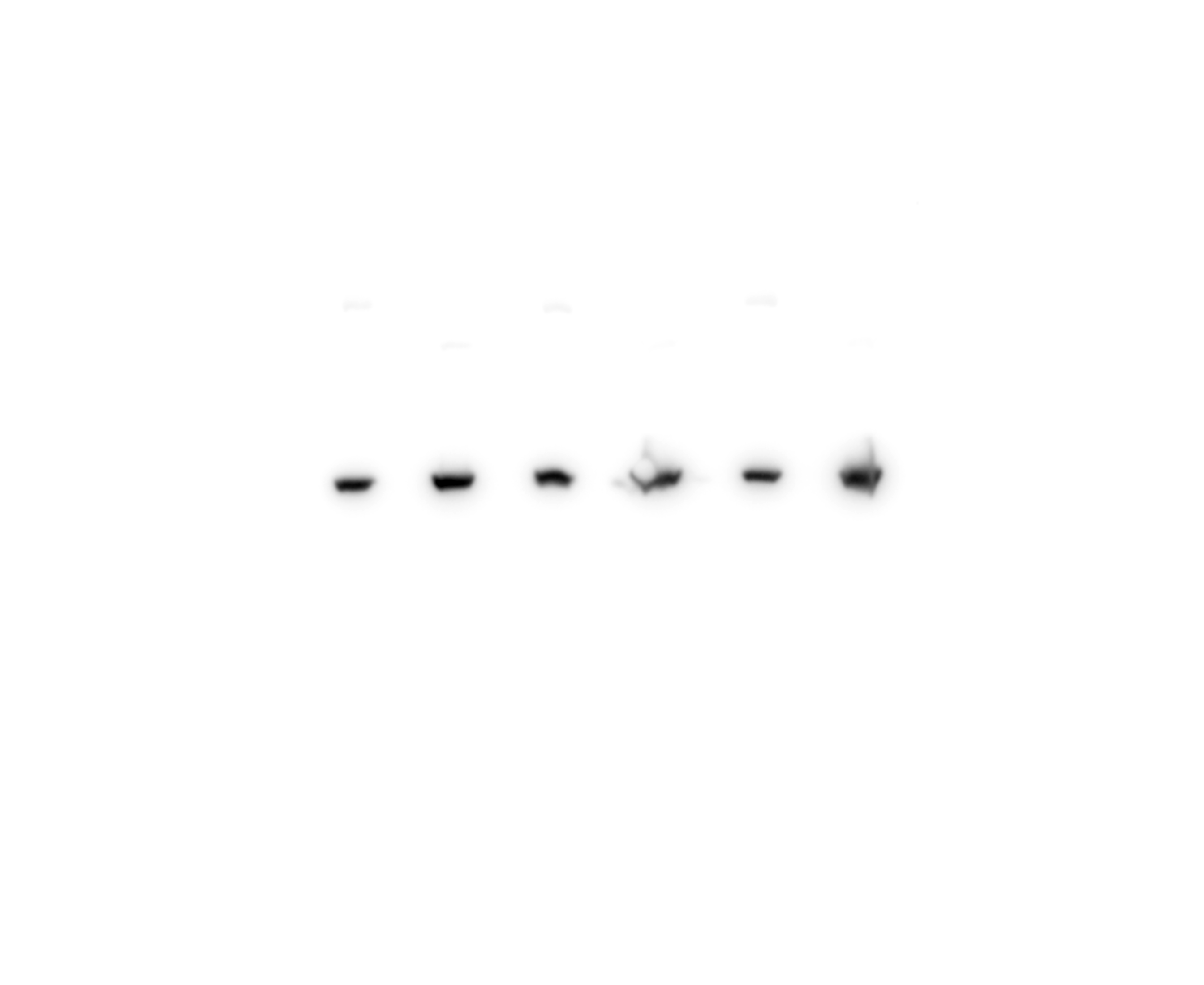

Supplement: Source data 3. [file elife-77755-data3.zip › Figure3-figure supplement1/Fig3-S1C FLAG (Tara).tif]

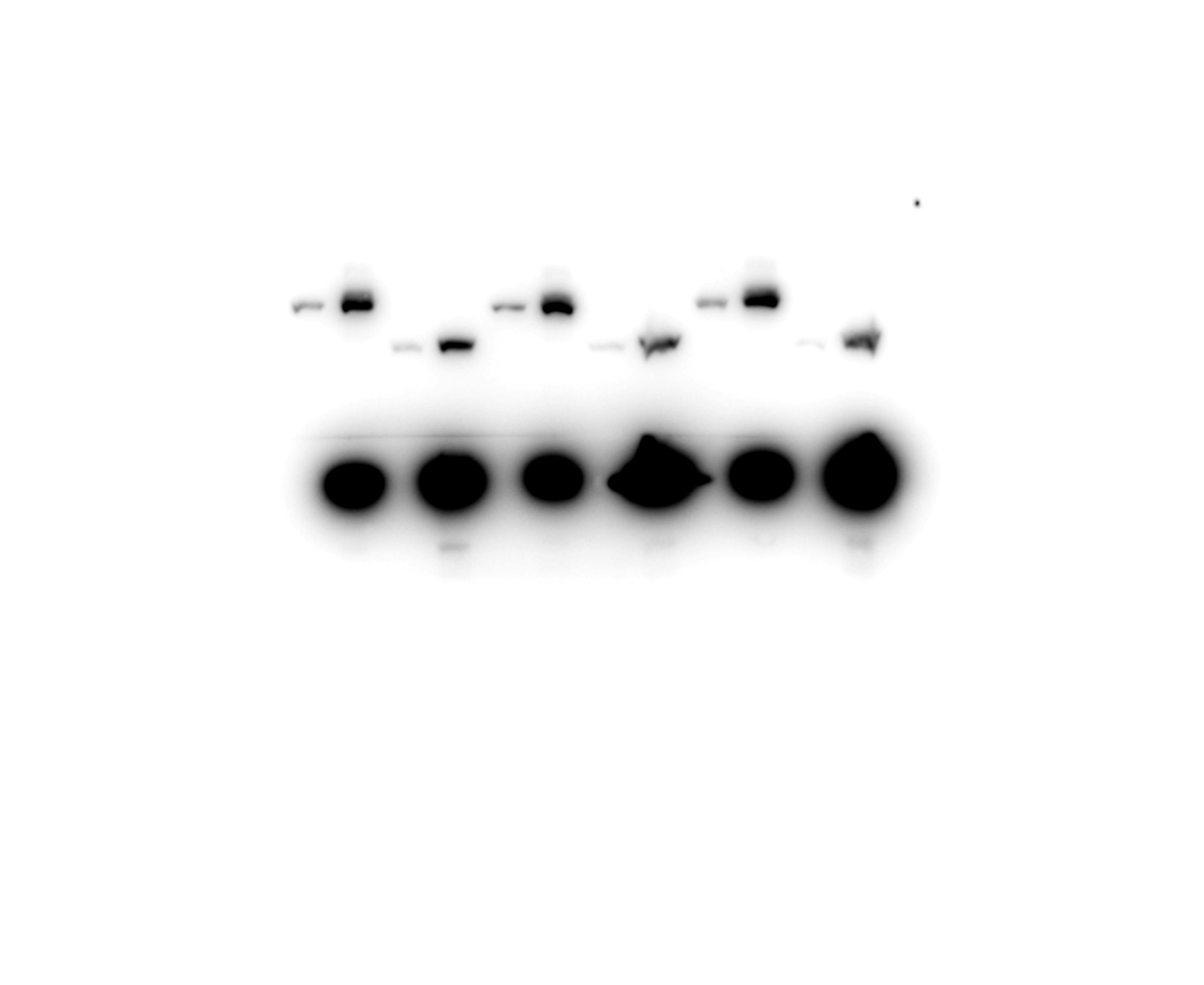

Supplement: Source data 3. [file elife-77755-data3.zip › Figure3-figure supplement1/Fig3-S1C GFP (Rai14).tif]

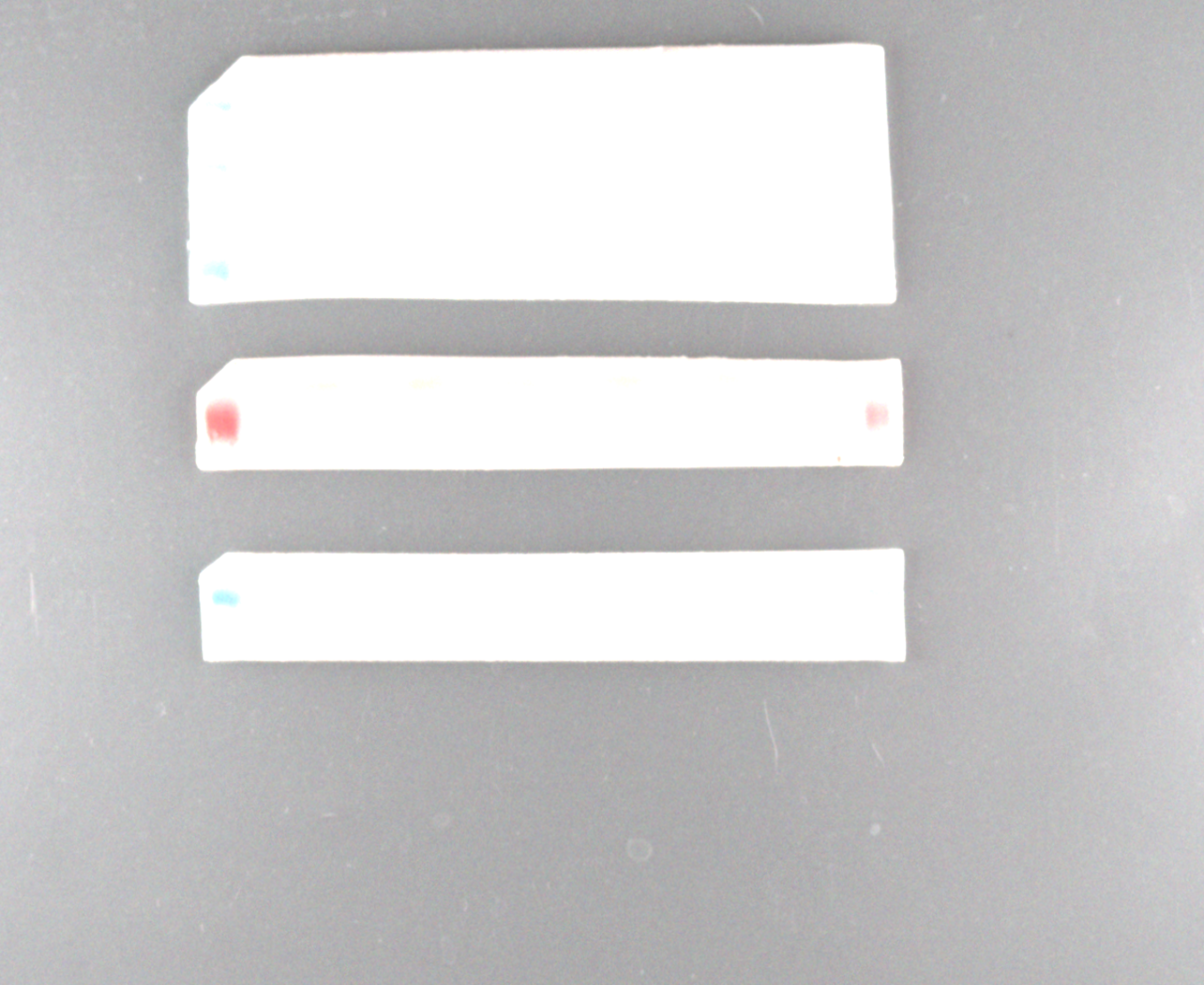

Supplement: Source data 3. [file elife-77755-data3.zip › Figure3-figure supplement1/Fig3-S1C-size marker for alpha-tubulin.tif]

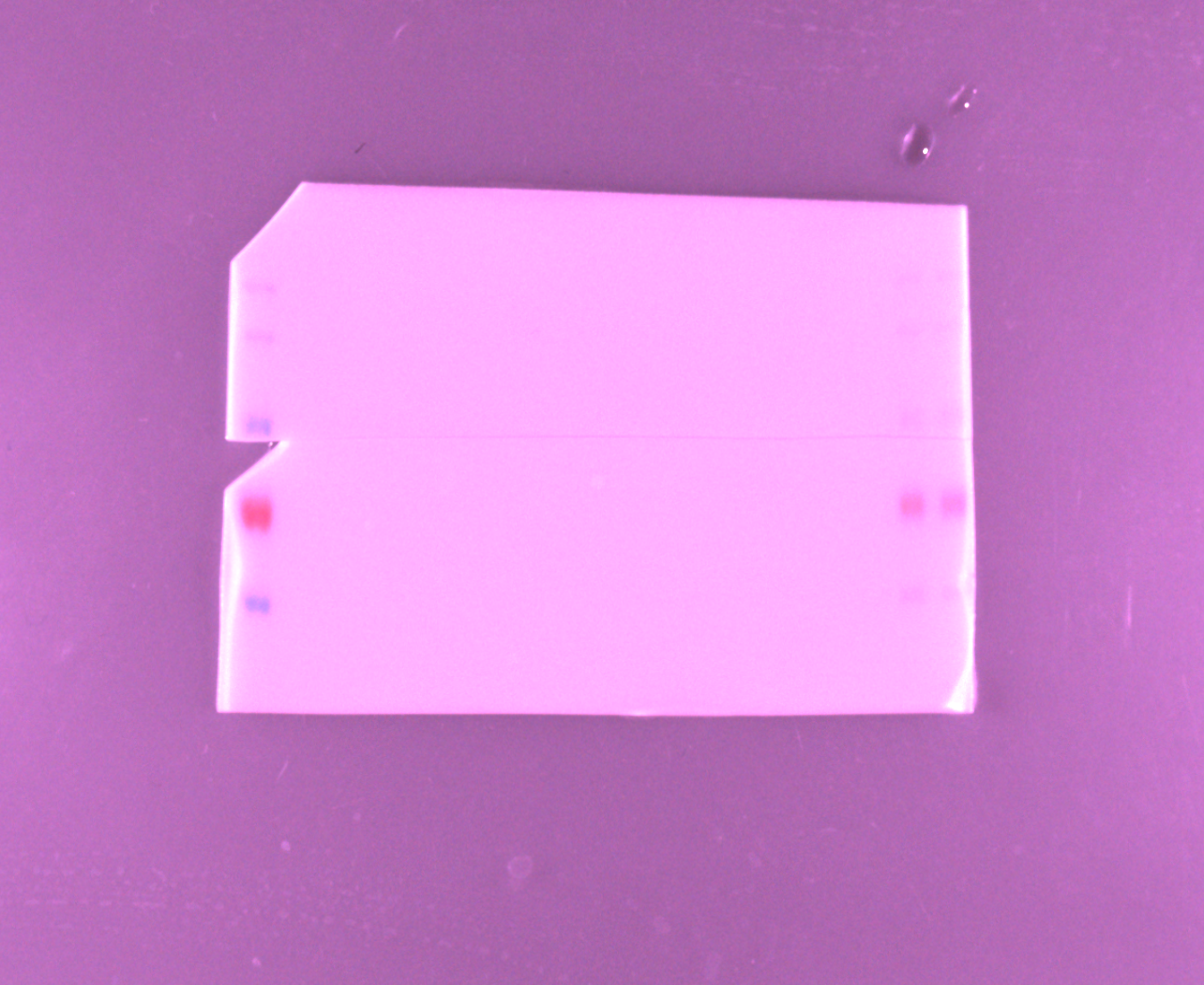

Supplement: Source data 3. [file elife-77755-data3.zip › Figure3-figure supplement1/Fig3-S1C-size marker for GFP (Rai14) and FLAG (Tara).tif]
