# Supplementary material for: Retinoic acid-induced protein 14 controls dendritic spine dynamics associated with depressive-like behaviors
Source: eLife. 2022 Apr 25;11:e77755. doi: 10.7554/eLife.77755 (PMC9068211; doi:10.7554/eLife.77755)
Supplement: Figure 3—figure supplement 1—source data 1. [file elife-77755-fig3-figsupp1-data1.pdf]

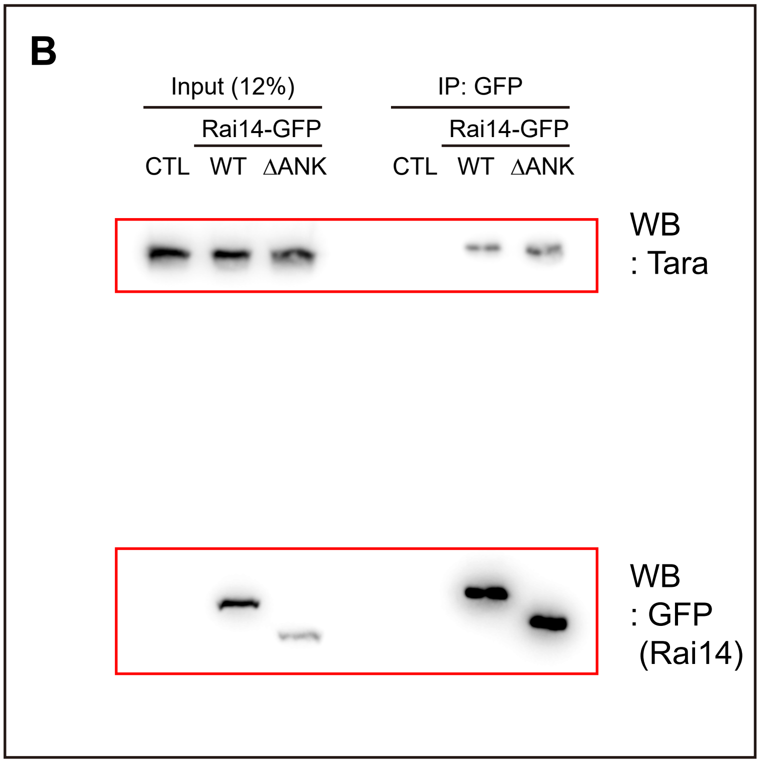

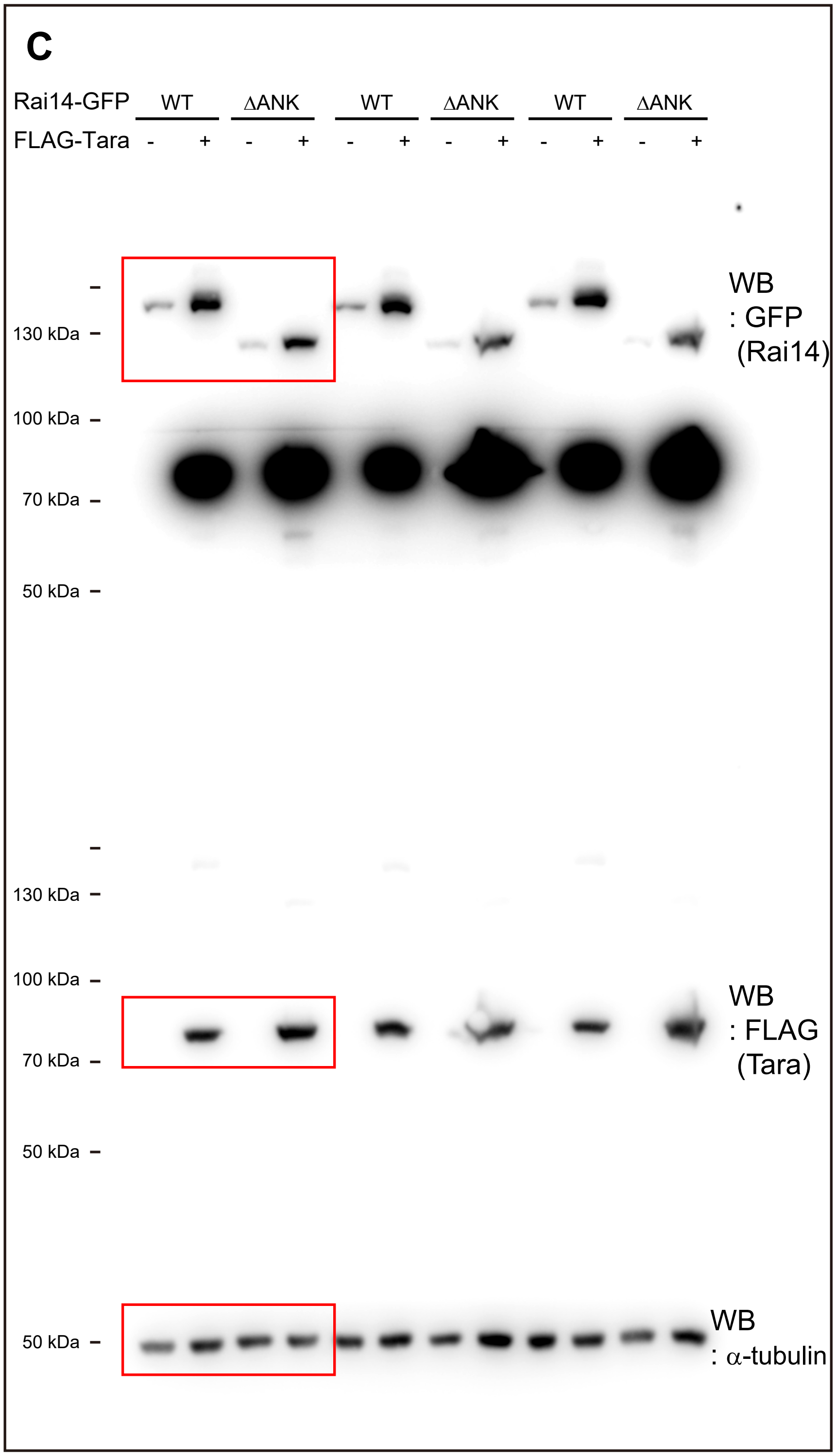

Figure3–figure supplement 1
