# Supplementary material for: Retinoic acid-induced protein 14 controls dendritic spine dynamics associated with depressive-like behaviors
Source: eLife. 2022 Apr 25;11:e77755. doi: 10.7554/eLife.77755 (PMC9068211; doi:10.7554/eLife.77755)
Supplement: Transparent reporting form [file elife-77755-transrepform1.docx]

***eLife’s* transparent reporting form**

We encourage authors to provide detailed information *within their submission* to facilitate the interpretation and replication of experiments. Authors can upload supporting documentation to indicate the use of appropriate reporting guidelines for health-related research (see [EQUATOR Network](http://www.equator-network.org/%20)), life science research (see the [BioSharing Information Resource](https://biosharing.org/)), or the [ARRIVE guidelines](http://www.plosbiology.org/article/info:doi/10.1371/journal.pbio.1000412) for reporting work involving animal research. Where applicable, authors should refer to any relevant reporting standards documents in this form.

If you have any questions, please consult our Journal Policies and/or contact us: [editorial@elifesciences.org](mailto:editorial@elifesciences.org).

**Sample-size estimation**

- You should state whether an appropriate sample size was computed when the study was being designed
- You should state the statistical method of sample size computation and any required assumptions
- If no explicit power analysis was used, you should describe how you decided what sample (replicate) size (number) to use

Please outline where this information can be found within the submission (e.g., sections or figure legends), or explain why this information doesn’t apply to your submission:

For all experiments, sample size was chosen taking in consideration the means of the target values between the experimental group and the control group. The minimum number of animals necessary to achieve the scientific objectives was used following guidelines of IACUC (Institutional Animal Care and Use Committee) of POSTECH.

**Replicates**

- You should report how often each experiment was performed
- You should include a definition of biological versus technical replication
- The data obtained should be provided and sufficient information should be provided to indicate the number of independent biological and/or technical replicates
- If you encountered any outliers, you should describe how these were handled
- Criteria for exclusion/inclusion of data should be clearly stated
- High-throughput sequence data should be uploaded before submission, with a private link for reviewers provided (these are available from both GEO and ArrayExpress)

Please outline where this information can be found within the submission (e.g., sections or figure legends), or explain why this information doesn’t apply to your submission:

The numbers of biological replicates (e.g. number of neurons or mice) and technical replicates (number of experiment repetition) are stated in the figure legends wherever applicable.

No data was excluded except for the fear conditioning test. A significant outlier was detected from the WT group in the context B of fear conditioning test by performing Grubb’s test. Data of the mouse corresponding to the outlier of context B were excluded from both context A and context B of the fear conditioning test.

**Statistical reporting**

- Statistical analysis methods should be described and justified
- Raw data should be presented in figures whenever informative to do so (typically when N per group is less than 10)
- For each experiment, you should identify the statistical tests used, exact values of N, definitions of center, methods of multiple test correction, and dispersion and precision measures (e.g., mean, median, SD, SEM, confidence intervals; and, for the major substantive results, a measure of effect size (e.g., Pearson's r, Cohen's d)
- Report exact p-values wherever possible alongside the summary statistics and 95% confidence intervals. These should be reported for all key questions and not only when the p-value is less than 0.05.

Please outline where this information can be found within the submission (e.g., sections or figure legends), or explain why this information doesn’t apply to your submission:

Statistical analysis methods are presented in the figure legends and the Materials and Methods (Statistical Analysis section).

(For large datasets, or papers with a very large number of statistical tests, you may upload a single table file with tests, Ns, etc., with reference to sections in the manuscript.)

**Group allocation**

- Indicate how samples were allocated into experimental groups (in the case of clinical studies, please specify allocation to treatment method); if randomization was used, please also state if restricted randomization was applied
- Indicate if masking was used during group allocation, data collection and/or data analysis

Please outline where this information can be found within the submission (e.g., sections or figure legends), or explain why this information doesn’t apply to your submission:

For dendritic spine analysis, pyramidal neurons were randomly chosen for imaging as stated in Materials and Methods (Microscopy section). For *in vitro* dendritic spine analysis (Fig 1 F–G, Fig 2 D, H–I, Fig 3, Fig 4A–C), dissociated neurons from multiple embryonic brains from single pregnant mouse were seeded on multiple coverslips and each coverslip was randomly allocated for each transfection group. For dendritic spine analysis from Golgi-stained neurons (Fig 1 A–B, Fig 5H), mice were randomly allocated for each genotype groups with partial restriction (within littermates). For chronic restraint stress model (Fig 5 I–K), mice were randomly allocated for each experimental groups.

**Additional data files (“source data”)**

- We encourage you to upload relevant additional data files, such as numerical data that are represented as a graph in a figure, or as a summary table
- Where provided, these should be in the most useful format, and they can be uploaded as “Source data” files linked to a main figure or table
- Include model definition files including the full list of parameters used
- Include code used for data analysis (e.g., R, MatLab)
- Avoid stating that data files are “available upon request”

Please indicate the figures or tables for which source data files have been provided:

Source data files including the numerical data associated with the figures are provided (for figures 1, 2, 3, 4, and 5). The source data files with original uncropped western blot images are also provided as a PDF file (figures with the uncropped gels with relevant band labelled) and a zipped folder (the original files of the raw unedited gels).

Sequencing data have been deposited at Dryad (doi:10.5061/dryad.1rn8pk0w9); (https://datadryad.org/stash/share/E85RkoQgrEMPk9ER8Xdtbx3Y0gdJcHinRl6rtlmfXek).
